# iSNO-AAPair: incorporating amino acid pairwise coupling into PseAAC for predicting cysteine *S*-nitrosylation sites in proteins

**DOI:** 10.7717/peerj.171

**Published:** 2013-10-03

**Authors:** Yan Xu, Xiao-Jian Shao, Ling-Yun Wu, Nai-Yang Deng, Kuo-Chen Chou

**Affiliations:** 1 Department of Information and Computer Science, University of Science and Technology Beijing, Beijing, China; 2 Department of Mathematics and Information Science, Bin-Zhou University, Bin-Zhou, China; 3 Institute of Applied Mathematics, Academy of Mathematics and Systems Science, Chinese Academy of Sciences, Beijing, China; 4 College of Science, China Agricultural University, Beijing, China; 5 Center of Excellence in Genomic Medicine Research (CEGMR), King Abdulaziz University, Jeddah, Saudi Arabia; 6 Gordon Life Science Institute, Belmont, MA, USA

**Keywords:** Pseudo amino acid composition, Position-specific amino acid propensity, Post-translational modification, Nearest neighbor pair, *S*-nitrosylation, Next nearest neighbor pair

## Abstract

As one of the most important and universal posttranslational modifications (PTMs) of proteins, *S*-nitrosylation (SNO) plays crucial roles in a variety of biological processes, including the regulation of cellular dynamics and many signaling events. Knowledge of SNO sites in proteins is very useful for drug development and basic research as well. Unfortunately, it is both time-consuming and costly to determine the SNO sites purely based on biological experiments. Facing the explosive protein sequence data generated in the post-genomic era, we are challenged to develop automated vehicles for timely and effectively determining the SNO sites for uncharacterized proteins. To address the challenge, a new predictor called iSNO-AAPair was developed by taking into account the coupling effects for all the pairs formed by the nearest residues and the pairs by the next nearest residues along protein chains. The cross-validation results on a state-of-the-art benchmark have shown that the new predictor outperformed the existing predictors. The same was true when tested by the independent proteins whose experimental SNO sites were known. A user-friendly web-server for iSNO-AAPair was established at http://app.aporc.org/iSNO-AAPair/, by which users can easily obtain their desired results without the need to follow the mathematical equations involved during its development.

## Introduction

**Figure 1:**
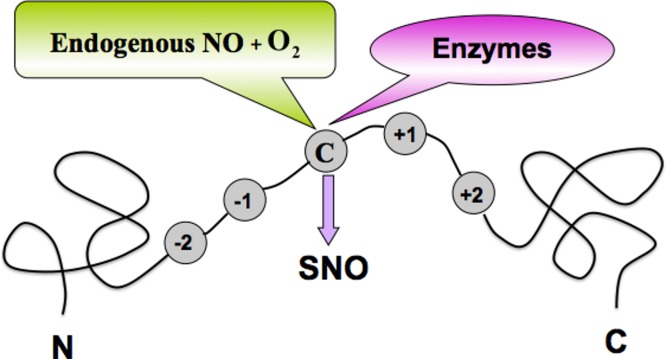
A schematic drawing to show the *S*-nitrosylation (SNO) site of a protein.

Regulating the stability and the functions of proteins (Mann & Jensen, 2003; Walsh & Jefferis, 2006), the post-translational modifications (PTMs) play important roles in a variety of biological processes, including transcriptional regulation (Li et al., 2007), cell signaling (Whalen et al., 2007) and apoptosis (Lugovskoy et al., 1999; Tsang et al., 2009). The aberrances of the PTMs are closely associated with devastating diseases such as cancers (Lahiry et al., 2010), Parkinson’s (Uehara et al., 2006; Yao et al., 2004), and Alzheimer’s (Carter & Chou, 1998; Cho et al., 2009). One of the most important and universal PTMs is *S*-nitrosylation (SNO). Therefore, identifying the SNO sites in proteins (Fig. 1) is crucially important for both biomedical research and drug development.

Actually, many efforts have been made to identify the SNO sites with experimental approaches, such as BST (biotin switch assay) (Jaffrey et al., 2001), SNOSID (Derakhshan, Wille & Gross, 2007; Greco et al., 2006), and SNO-RAC (Forrester et al., 2009). Although considerable knowledge about the SNO sites could be obtained by these methods, it is both time-consuming and laborious by means of the experimental approaches alone. Facing the explosion of protein sequences generated in the post genomic era, we are challenged to develop computational method for fast and reliably identifying the SNO sites in proteins.

Recently, several computational methods have been proposed in this regard (Li et al., 2011; Li et al., 2012; Xue et al., 2010; Xu et al., 2013). Each of these methods has merit and did play a role in stimulating the development of this area. However, they also each have their own limits. For example, by incorporating the position specific amino acid propensity into the general form of pseudo amino acid composition (Chou, 2001a) or Chou’s PseAAC (Lin & Lapointe, 2013), the authors in a recent article (Xu et al., 2013) presented a predictor called iSNO-PseAAC, which can yield higher success rates than the other existing methods for predicting SNO sites. However, in the iSNO-PseAAC predictor, only the position propensity of each of the constituent amino acids was considered without taking into account any of their correlation. In other words, all the amino acids in the proteins were treated independently. However, in the real world, they are not independent of each other but bear some sort of correlation. And incorporating the correlation effects could really improve the prediction quality accordingly, such as in identifying the peptide cleavage sites by signal peptidase (Chou, 2001d), investigating the specificity of GalNAc-transferase (Chou, 1995), predicting the protein cleavage sites by HIV-protease (Chou, 1993), as well as using the information thus obtained to develop peptide-drugs against HIV/AIDS and SARS (Du, Sun & Chou, 2007; Du et al., 2005; Gan et al., 2006; Shen & Chou, 2008) based on Chou’s distorted key theory (Chou, 1996). Motivated and encouraged by these studies, here we are to develop a new method for identifying the protein SNO sites by incorporating some sequence correlation effects.

As shown by a series of recent publications (Chen et al., 2013; Chen et al., 2012b; Xiao et al., 2013) and summarized in a comprehensive review (Chou, 2011), to establish a really useful statistical predictor for a sequence-based system, one needs to engage the following procedures: (i) construct or select a valid benchmark dataset to train and test the predictor; (ii) formulate the sequence samples with an effective mathematical expression that can truly reflect their intrinsic correlation with the target to be predicted; (iii) introduce or develop a powerful algorithm (or engine) to operate the prediction; (iv) properly perform cross-validation tests to objectively evaluate the anticipated accuracy of the predictor; (v) establish a user-friendly web-server for the predictor that is accessible to the public. Below, let us describe how to engage these procedures one by one.

## Materials and Methods

### Benchmark dataset

In this study the benchmark dataset was derived from the *S*-nitrosylated database (version 1.0) (Chen et al., 2010) at http://dbsno.mbc.nctu.edu.tw/, from which 1,530 proteins in human and mouse species and their SNO sites were downloaded. The corresponding peptide fragments for these SNO sites were derived from UniProt database (release 2012_08). To facilitate description later, let us adopt Chou’s formulation for peptides here that was used for studying signal peptide cleavage sites (Chou, 2001c; Chou, 2001d). According to the formulation, a peptide with cysteine located at its center (Fig. 1) can be written as

**Figure 2:**
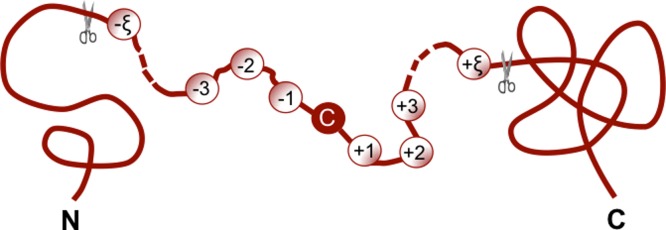
A schematic illustration to show a peptide generated from a protein sequence by the sliding window (Chou, 2001d) with cysteine (C) located at its center. Adapted from Chou (Chou, 2001b) with permission.

(1) \begin{eqnarray*} \displaystyle \mathbf{P}={\mathrm{R}}_{-\xi }{\mathrm{R}}_{-(\xi -1)}\ldots {\mathrm{R}}_{-2}{\mathrm{R}}_{-1}\mathbf{C}~{\mathrm{R}}_{+1}{\mathrm{R}}_{+2}\ldots {\mathrm{R}}_{+(\xi -1)}{\mathrm{R}}_{+\xi }&&\displaystyle \end{eqnarray*}

where the subscript ξ is an integer, R_−ξ_ represents the ξ-th downstream amino acid residue from cysteine (C), R_ξ_ the ξ-th upstream amino acid residue, and so forth (Fig. 2). Peptides with the profile of Eq. (1) can be further classified into the following two categories: (1) SNO peptide if its center is a SNO site; (2) non-SNO peptide if its center is a non-SNO site, as can be formulated by

(2) \begin{eqnarray*} \displaystyle \mathbf{P}\in \left\{\begin{array}{@{}l@{}} \displaystyle \text{SNO peptide},\quad \text{if C is a SNO site }\\ \displaystyle \text{non-SNO peptide},\quad \text{otherwise } \end{array}\right.&&\displaystyle \end{eqnarray*}

where ∈ represents “a member of” in the set theory. After some preliminary trials and also considering the practice of previous investigators (Li et al., 2011; Li et al., 2012; Xue et al., 2010; Xu et al., 2013), we choose ξ = 10 to construct the benchmark dataset for **P** of Eq. (1). If the upstream or downstream in a protein was less than 10, the lacking residues were filled with the dummy code Z. The peptides thus obtained are subject to a screening procedure to winnow those that have ≥40% sequence identity to any other. Finally, we obtained 2,381 SNO peptides and 11,755 non-SNO peptides. Now let us construct the training or learning dataset 𝕊_L_ as defined by

(3) \begin{eqnarray*} \displaystyle {\mathbb{S}}_{\mathrm{L}}={\mathbb{S}}_{\mathrm{L}}^{+}\cup {\mathbb{S}}_{\mathrm{L}}^{-}&&\displaystyle \end{eqnarray*}

where ∪ represents the “union” in the set theory, ${\mathbb{S}}_{\mathrm{L}}^{+}$ contains 2,300 samples randomly picked from the aforementioned 2,381 SNO peptides, while ${\mathbb{S}}_{\mathrm{L}}^{-}$ 2,300 samples randomly picked from the 11,755 non-SNO peptides. For readers’ convenience, the 2,300 peptide sequences in the positive learning dataset ${\mathbb{S}}_{\mathrm{L}}^{+}$ and 2,300 peptide sequences in the negative learning dataset ${\mathbb{S}}_{\mathrm{L}}^{-}$, along with their sequence positions (sites) in the parent proteins coded in “UniProt IDs”, are given in Supplemental Information S1.

Moreover, for the purpose of demonstration later, let us also construct an independent dataset 𝕊_T_ given by

(4) \begin{eqnarray*} \displaystyle {\mathbb{S}}_{\mathrm{T}}={\mathbb{S}}_{\mathrm{T}}^{+}\cup {\mathbb{S}}_{\mathrm{T}}^{-}&&\displaystyle \end{eqnarray*}

where ${\mathbb{S}}_{\mathrm{T}}^{+}$ contains the remaining 81 samples in the aforementioned 2,381 SNO peptides, while ${\mathbb{S}}_{\mathrm{T}}^{-}$ contains 100 samples randomly picked from the 11,755 non-SNO peptides but none of them occurs in ${\mathbb{S}}_{\mathrm{L}}^{-}$. Likewise, the 81 peptide sequences in the positive testing dataset ${\mathbb{S}}_{\mathrm{T}}^{+}$ and 100 peptide sequences in the negative testing dataset ${\mathbb{S}}_{\mathrm{T}}^{-}$ are given in Supplemental Information S2.

### Feature vector construction

In developing a statistical method for predicting the cleavage sites (Chou, 1993) in proteins or their attributes (Chou, 1995), one of the important procedures was to formulate the protein or peptide samples with an effective mathematical expression that could truly reflect the intrinsic correlation with the desired target. To realize this, various different vectors were proposed (see, Cao, Xu & Liang, 2013; Chen & Li, 2013; Du et al., 2012; Esmaeili, Mohabatkar & Mohsenzadeh, 2010; Fan & Li, 2012; Khosravian et al., 2013; Liu et al., 2012; Mohabatkar et al., 2013; Mohabatkar, Mohammad Beigi & Esmaeili, 2011; Nanni et al., 2010; Wan, Mak & Kung, 2013; Yu et al., 2010; Zhang et al., 2008a; Zhou et al., 2007) to formulate proteins or peptides by extracting their different features into the pseudo amino acid composition (Chou, 2001a) or Chou’s PseAAC (Lin & Lapointe, 2013).

According to a recent review (Chou, 2011), the general form of Chou’s PseAAC for a protein or peptide **P** can be formulated by

(5) \begin{eqnarray*} \displaystyle \mathbf{P}={\left[\begin{array}{@{}cccccc@{}} \displaystyle {\psi }_{1}&\displaystyle {\psi }_{2}&\displaystyle \ldots &\displaystyle {\psi }_{u}&\displaystyle \ldots &\displaystyle {\psi }_{\Omega } \end{array}\right]}^{\mathbf{T}}&&\displaystyle \end{eqnarray*}

where **T** is the transpose operator, while Ω is an integer to reflect the vector’s dimension. The value of Ω as well as the components ψ_*u*_ (*u* = 1, 2, …, Ω) in Eq. (5) will depend on how to extract the desired information from the protein or peptide sequence. Below, let us describe how to extract the useful information from the learning dataset 𝕊_L_ to define the peptide samples via Eq. (5) for the current study.

Since the length of each peptide in the training dataset 𝕊_L_ is 21 (cf. Supplemental Information S1), Eq. (1) for **P** can be simplified to a more convenient form given by

(6) \begin{eqnarray*} \displaystyle \mathbf{P}={\mathrm{R}}_{1}{\mathrm{R}}_{2}\ldots {\mathrm{R}}_{9}{\mathrm{R}}_{10}{\mathrm{R}}_{11}{\mathrm{R}}_{12}\ldots {\mathrm{R}}_{20}{\mathrm{R}}_{21}&&\displaystyle \end{eqnarray*}

where R_11_ = C and R_*i*_ (*i* = 1, 2, …, 21; *i* ≠ 11) can be any of the 20 native amino acids or the dummy code Z as defined above. Hereafter, let us use the numerical codes 1, 2, 3, …, 20 to represent the 20 native amino acids according to the alphabetic order of their single letter codes, and use 21 to represent the dummy amino acid Z. Accordingly, the number of possible different dipeptides will be 21 × 21 = 441, and the number of dipeptide subsite positions on the sequence of Eq. (6) will be (21−2 + 1) = 20.

Now, let us introduce the following 441 × 20 matrix ℤ^0^, the so-called PSDP (position-specific dipeptide propensity) matrix to define the component of Eq. (5)

(7) \begin{eqnarray*} \displaystyle {\mathbb{Z}}^{0}=\left[\begin{array}{@{}cccc@{}} \displaystyle {z}_{1,1}^{0}&\displaystyle {z}_{1,2}^{0}&\displaystyle \ldots &\displaystyle {z}_{1,20}^{0}\\ \displaystyle {z}_{2,1}^{0}&\displaystyle {z}_{2,2}^{0}&\displaystyle \ldots &\displaystyle {z}_{2,20}^{0}\\ \displaystyle \vdots &\displaystyle \vdots &\displaystyle \ddots &\displaystyle \vdots \\ \displaystyle {z}_{441,1}^{0}&\displaystyle {z}_{441,2}^{0}&\displaystyle \ldots &\displaystyle {z}_{441,20}^{0} \end{array}\right]&&\displaystyle \end{eqnarray*}

where the element

(8) \begin{eqnarray*} \displaystyle {z}_{i,j}^{0}={F}_{0}^{+}({\mathrm{D}}_{i}^{0}\vert j)-{F}_{0}^{-}({\mathrm{D}}_{i}^{0}\vert j)\quad (i=1,2,\ldots ,441;j=1,2,\ldots ,20)&&\displaystyle \end{eqnarray*}

and

(9) \begin{eqnarray*} \displaystyle {\mathrm{D}}_{1}^{0}=\mathrm{AA},{\mathrm{D}}_{2}^{0}=\mathrm{AC},{\mathrm{D}}_{3}^{0}=\mathrm{AD},\ldots ,{\mathrm{D}}_{440}^{0}=\mathrm{ZY},{\mathrm{D}}_{441}^{0}=\mathrm{ZZ}.&&\displaystyle \end{eqnarray*}

In Eq. (8), ${F}_{0}^{+}({\mathrm{D}}_{i}^{0}\vert j)$ is the occurrence frequency of the *i*-th dipeptide (*i* = 1, 2, …, 441) at the *j*-th subsite on the sequence of Eq. (6) (or the *j*-th column in the positive learning dataset ${\mathbb{S}}_{\mathrm{L}}^{+})$ that can be easily derived using the method described in (Chou, 2001d) from the sequences in Supplemental Information S1; while ${F}_{0}^{-}({\mathrm{D}}_{i}^{0}\vert j)$ is the corresponding occurrence frequency but derived from the negative learning dataset ${\mathbb{S}}_{\mathrm{L}}^{-}$.

**Figure 3:**
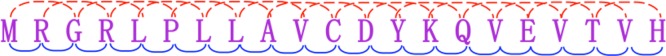
A schematic drawing to show the pairwise coupling between nearest residues (blue solid line) and that between the next nearest residues (red dashed line).

In order to extract more information, let us expand the propensity matrix from the dipeptide (or the residue pair formed by the nearest residues) to the pair formed by the next nearest amino acid residues (Fig. 3). Since the number of possible such amino acid pairs is still 21 × 21 = 441, but the number of their subsite positions on the sequence of Eq. (6) is reduced to (21−3 + 1) = 19, the corresponding position-specific propensity matrix should be given by

(10) \begin{eqnarray*} \displaystyle {\mathbb{Z}}^{1}=\left[\begin{array}{@{}cccc@{}} \displaystyle {z}_{1,1}^{1}&\displaystyle {z}_{1,2}^{1}&\displaystyle \ldots &\displaystyle {z}_{1,19}^{1}\\ \displaystyle {z}_{2,1}^{1}&\displaystyle {z}_{2,2}^{1}&\displaystyle \ldots &\displaystyle {z}_{2,19}^{1}\\ \displaystyle \vdots &\displaystyle \vdots &\displaystyle \ddots &\displaystyle \vdots \\ \displaystyle {z}_{441,1}^{1}&\displaystyle {z}_{441,2}^{1}&\displaystyle \ldots &\displaystyle {z}_{441,19}^{1} \end{array}\right]&&\displaystyle \end{eqnarray*}

where the element

(11) \begin{eqnarray*} \displaystyle {z}_{i,j}^{1}={F}_{1}^{+}({\mathrm{D}}_{i}^{1}\vert j)-{F}_{1}^{-}({\mathrm{D}}_{i}^{1}\vert j)\quad (i=1,2,\ldots ,441;j=1,2,\ldots ,19)&&\displaystyle \end{eqnarray*}

where ${\mathrm{D}}_{i}^{1}$ has the same meaning as ${\mathrm{D}}_{i}^{0}$ in Eq. (9) but instead of dipeptide it represents the pairs of amino acids separated by one residue between them along a protein sequence. Likewise, ${F}_{1}^{+}({\mathrm{D}}_{i}^{1}\vert j)$ and ${F}_{1}^{-}({\mathrm{D}}_{i}^{1}\vert j)$ also have the similar meaning as ${F}_{0}^{+}({\mathrm{D}}_{i}\vert j)$ and ${F}_{0}^{-}({\mathrm{D}}_{i}\vert j)$ in Eq. (8), and can be easily derived from the sequences in Supplemental Information S1 as well.

Now, let us define a new matrix ℤ by merging ℤ^0^ and ℤ^1^; i.e.,

(12) \begin{eqnarray*} \displaystyle \mathbb{Z}={\mathbb{Z}}^{0}\oplus {\mathbb{Z}}^{1}=\left(\begin{array}{@{}cccccccc@{}} \displaystyle {z}_{1,1}^{0}&\displaystyle {z}_{1,2}^{0}&\displaystyle \ldots &\displaystyle {z}_{1,20}^{0}&\displaystyle {z}_{1,1}^{1}&\displaystyle {z}_{1,2}^{1}&\displaystyle \ldots &\displaystyle {z}_{1,19}^{1}\\ \displaystyle {z}_{2,1}^{0}&\displaystyle {z}_{2,2}^{0}&\displaystyle \ldots &\displaystyle {z}_{2,20}^{0}&\displaystyle {z}_{2,1}^{1}&\displaystyle {z}_{2,2}^{1}&\displaystyle \ldots &\displaystyle {z}_{2,19}^{1}\\ \displaystyle \vdots &\displaystyle \vdots &\displaystyle \ddots &\displaystyle \vdots &\displaystyle \vdots &\displaystyle \vdots &\displaystyle \ddots &\displaystyle \vdots \\ \displaystyle {z}_{441,1}^{0}&\displaystyle {z}_{441,2}^{0}&\displaystyle \ldots &\displaystyle {z}_{441,20}^{0}&\displaystyle {z}_{441,1}^{1}&\displaystyle {z}_{441,2}^{1}&\displaystyle \ldots &\displaystyle {z}_{441,19}^{1} \end{array}\right)&&\displaystyle \end{eqnarray*}

where the symbol ⊕ represents the orthogonal sum (Chou & Shen, 2007). Thus, the peptide **P** of Eq. (6) can be uniquely defined via the general form of PseAAC (cf. Eq. (5)) with its dimension Ω = 20 + 19 = 39 and its *u*-th component given by

(13) \begin{eqnarray*} \displaystyle {\psi }_{u}=\left\{\begin{array}{@{}ll@{}} \displaystyle {z}_{1,u}^{0}&\displaystyle \text{when }{\mathrm{R}}_{u}{\mathrm{R}}_{u+1}=\mathrm{AA}\text{ and }1\leq u\leq 20\\ \displaystyle {z}_{2,u}^{0}&\displaystyle \text{when }{\mathrm{R}}_{u}{\mathrm{R}}_{u+1}=\mathrm{AC}\text{ and }1\leq u\leq 20\\ \displaystyle &\displaystyle \hspace{5.5pc} \vdots \\ \displaystyle {z}_{441,u}^{0}&\displaystyle \text{when }{\mathrm{R}}_{u}{\mathrm{R}}_{u+1}=\mathrm{ZZ}\text{ and }1\leq u\leq 20\\ \displaystyle {z}_{1,u}^{1}&\displaystyle \text{when }{\mathrm{R}}_{u}{\mathrm{R}}_{u+2}=\mathrm{AA}\text{ and }21\leq u\leq 39\\ \displaystyle {z}_{2,u}^{1}&\displaystyle \text{when }{\mathrm{R}}_{u}{\mathrm{R}}_{u+2}=\mathrm{AC}\text{ and }21\leq u\leq 39\\ \displaystyle &\displaystyle \hspace{5.5pc} \vdots \\ \displaystyle {z}_{441,u}^{1}&\displaystyle \text{when }{\mathrm{R}}_{u}{\mathrm{R}}_{u+2}=\mathrm{ZZ}\text{ and }21\leq u\leq 39 \end{array}\right.&&\displaystyle \end{eqnarray*}

where R_*u*_ is any residue in the *u*-th position of the peptide **P** (cf. Eq. (6)).

## Prediction Algorithm

Suppose ℙ^+^ and ℙ^−^ are the standard vectors or norms for the peptide sequences in ${\mathbb{S}}_{\mathrm{L}}^{+}$ and ${\mathbb{S}}_{\mathrm{L}}^{-}$, respectively. And they are defined by

(14) \begin{eqnarray*} \displaystyle \left\{\begin{array}{@{}l@{}} \displaystyle {\mathbb{P}}^{+}={\left[\begin{array}{@{}cccccc@{}} \displaystyle {\bar {\psi }}_{1}^{+}&\displaystyle {\bar {\psi }}_{2}^{+}&\displaystyle \ldots &\displaystyle {\bar {\psi }}_{u}^{+}&\displaystyle \ldots &\displaystyle {\bar {\psi }}_{\Omega }^{+} \end{array}\right]}^{T}\\ \\ \displaystyle {\mathbb{P}}^{-}={\left[\begin{array}{@{}cccccc@{}} \displaystyle {\bar {\psi }}_{1}^{-}&\displaystyle {\bar {\psi }}_{2}^{-}&\displaystyle \ldots &\displaystyle {\bar {\psi }}_{u}^{-}&\displaystyle \ldots &\displaystyle {\bar {\psi }}_{\Omega }^{-} \end{array}\right]}^{T} \end{array}\right.&&\displaystyle \end{eqnarray*}

where

(15) \begin{eqnarray*} \displaystyle \left\{\begin{array}{@{}l@{}} \displaystyle {\bar {\psi }}_{u}^{+}=\frac{1}{{N}^{+}}\sum _{k=1}^{{N}^{+}}\psi _{u,k}^{+}\\ \\ \displaystyle {\bar {\psi }}_{u}^{-}=\frac{1}{{N}^{-}}\sum _{k=1}^{{N}^{-}}\psi _{u,k}^{-} \end{array}\right.\quad (u=1,2,\ldots ,\Omega )&&\displaystyle \end{eqnarray*}

where *N*^+^ is the total number of SNO peptides in the learning dataset, and ${\psi }_{u,k}^{+}$ the *u*-th component for the *k*-th SNO peptide in the PseAAC space (cf. Eqs. (5) and (13)); whereas *N*^−^ and ${\psi }_{u,k}^{-}$ have the same meanings but are for the non-SNO peptides.

For a query peptide **P** as formulated by Eq. (5), suppose 𝔻 (**P**, ℙ^+^) is its similarity to the norm of SNO peptides, and 𝔻 (**P**, ℙ^−^) its similarity to the norm of non-SNO peptides, as formulated by

(16) \begin{eqnarray*} \displaystyle \left\{\begin{array}{@{}l@{}} \displaystyle \mathbb{D}(\mathbf{P},{\mathbb{P}}^{+})=\sqrt{\sum _{u=1}^{\Omega }({\psi }_{u}-{\bar {\psi }}_{u}^{+})^{2}}\\ \\ \displaystyle \mathbb{D}(\mathbf{P},{\mathbb{P}}^{-})=\sqrt{\sum _{u=1}^{\Omega }{\left({\psi }_{u}-{\bar {\psi }}_{u}^{-}\right)}^{2}}\; . \end{array}\right.&&\displaystyle \end{eqnarray*}

Thus, the prediction rule for the query peptide **P** can be formulated as

(17) \begin{eqnarray*} \displaystyle \mathbf{P}\in \left\{\begin{array}{@{}l@{}} \displaystyle \text{SNO peptide},\quad \text{if }\mathbb{D}(\mathbf{P},{\mathbb{P}}^{+})\gt \mathbb{D}(\mathbf{P},{\mathbb{P}}^{-})\\ \displaystyle \text{non-SNO peptide},\quad \text{otherwise}. \end{array}\right.&&\displaystyle \end{eqnarray*}

If there was a tie between 𝔻 (**P**, ℙ^+^) and 𝔻 (**P**, ℙ^−^), the query peptide would be randomly assigned between the SNO peptide and non-SNO peptide categories. However, this kind of tie case rarely happened and actually never happened in our study.

The predictor established via the above procedures is called iSNO-AAPair, where “i” stands for the 1st character of “identify”, while “AAPair” means that the amino acid coupling effects were taken into account within the pairs formed by the nearest residues as well as the pairs formed by the next nearest residues along the peptide sequence.

**Figure 4:**
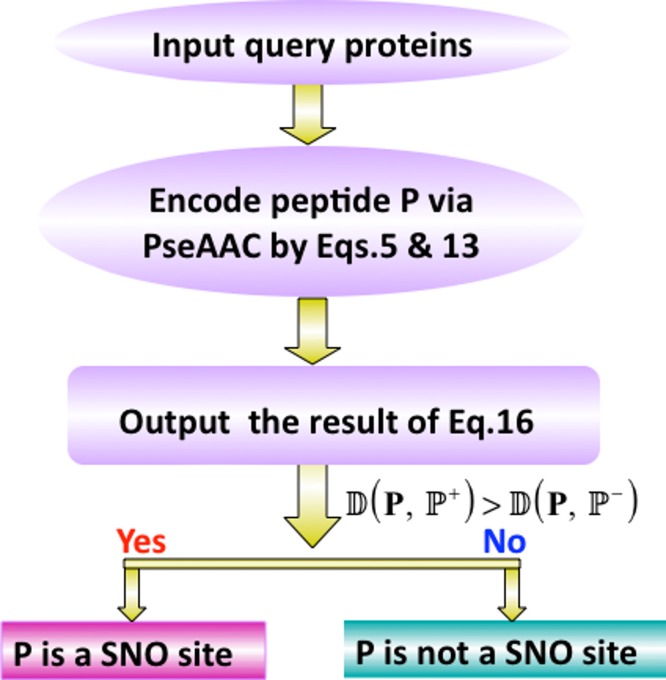
A flowchart showing the prediction process of iSNO-AAPair.

A flowchart of the predictor is given in Fig. 4 to illustrate how iSNO-AAPair was working during the process of prediction.

## Results and Discussion

How to objectively evaluate the performance of a predictor and how to make it easy to access by public (Chou & Shen, 2009) are two important factors that are directly associated with its application value. Below, let us address these problems.

### Four different metrics for measuring the prediction quality

In literature the following metrics are often used for examining the performance quality of a predictor

(18) \begin{eqnarray*} \displaystyle \left\{\extrarowheight =1.2pc \begin{array}{@{}l@{}} \displaystyle \text{Sn}=\frac{\text{TP}}{\text{TP}+\text{FN}}\\ \displaystyle \text{Sp}=\frac{\text{TN}}{\text{TN}+\text{FP}}\\ \displaystyle \text{Acc}=\frac{\text{TP + TN}}{\text{TP}+\text{TN}+\text{FP}+\text{FN}}\\ \displaystyle \text{MCC}=\frac{(\text{TP}\times \text{TN})-(\text{FP}\times \text{FN})}{\sqrt{\text{(TP + FP)(TP + FN)(TN + FP)(TN + FN)}}} \end{array}\right.&&\displaystyle \end{eqnarray*}

where TP represents the number of the true positive; TN, the number of the true negative; FP, the number of the false positive; FN, the number of the false negative; Sn, the sensitivity; Sp, the specificity; Acc, the accuracy; MCC, the Mathew’s correlation coefficient. To most biologists, however, the four metrics as formulated in Eq. (18) are not quite intuitive and easier-to-understand, particularly for the Mathew’s correlation coefficient. Here let us adopt the formulation proposed recently (Chen et al., 2013; Xu et al., 2013) in terms of the Chou’s symbol (Chou, 2001d); i.e.,

(19) \begin{eqnarray*} \displaystyle \left\{\extrarowheight =1.5pc \begin{array}{@{}l@{}} \displaystyle \text{Sn}=1-\frac{{N}_{-}^{+}}{{N}^{+}}\\ \displaystyle \text{Sp}=1-\frac{{N}_{+}^{-}}{{N}^{-}}\\ \displaystyle \text{Acc}=1-\frac{{N}_{-}^{+}+{N}_{+}^{-}}{{N}^{+}+{N}^{-}}\\ \displaystyle \text{MCC }=\frac{1-\left(\frac{{N}_{-}^{+}}{{N}_{+}}+\frac{{N}_{+}^{-}}{{N}^{-}}\right)}{\sqrt{\left(1+\frac{{N}_{+}^{-}-{N}_{-}^{+}}{{N}^{+}}\right)\left(1+\frac{{N}_{-}^{+}-{N}_{+}^{-}}{{N}^{-}}\right)}} \end{array}\right.&&\displaystyle \end{eqnarray*}

where *N*^+^ is the total number of the SNO peptides investigated while ${N}_{-}^{+}$ the number of the SNO peptides incorrectly predicted as the non-SNO peptides; *N*^−^ the total number of the non-SNO peptides investigated while ${N}_{+}^{-}$ the number of the non-SNO peptides incorrectly predicted as the SNO peptides (Chou, 2001b).

It can be clearly seen from Eq. (19) that when ${N}_{-}^{+}=0$ meaning none of the SNO peptides were incorrectly predicted to be a non-SNO peptide, we have sensitivity Sn = 1. When ${N}_{-}^{+}={N}^{+}$ meaning that all the SNO peptides were incorrectly predicted to be the non-SNO peptides, we have sensitivity Sn = 0. Likewise, when ${N}_{+}^{-}=0$ meaning none of the non-SNO peptides was incorrectly predicted to be the SNO peptide, we have specificity Sp = 1; whereas ${N}_{+}^{-}={N}^{-}$ meaning all the non-SNO peptides were incorrectly predicted as the SNO peptides, we have specificity Sp = 0. When ${N}_{-}^{+}={N}_{+}^{-}=0$ meaning that none of SNO peptides in the positive dataset and none of the non-SNO peptides in the negative dataset was incorrectly predicted, we have overall accuracy Acc = 1 and MCC = 1; when ${N}_{-}^{+}={N}^{+}$ and ${N}_{+}^{-}={N}^{-}$ meaning that all the SNO peptides in the positive dataset and all the non-SNO peptides in the negative dataset were incorrectly predicted, we have overall accuracy Acc = 0 and MCC = −1; whereas when ${N}_{-}^{+}={N}^{+}/2$ and ${N}_{+}^{-}={N}^{-}/2$ we have Acc = 0.5 and MCC = 0 meaning no better than random prediction. As we can see from the above discussion based on Eq. (19), the meanings of sensitivity, specificity, overall accuracy, and Mathew’s correlation coefficient have become much more intuitive and easier-to-understand.

It is instructive to point out that the set of metrics as given in Eq. (18) or Eq. (19) is valid only for the single-label systems as in the current case. For the multi-label systems whose emergence has become increasingly frequent in system biology (Chou, Wu & Xiao, 2011; Chou, Wu & Xiao, 2012) and system medicine (Chen et al., 2012a; Xiao et al., 2013), a different set of metrics as defined in Chou (2013) is needed.

### Cross-validation to evaluate the anticipated success rates

In statistical prediction, the following three cross-validation methods are often used to evaluate the anticipated accuracy of a predictor: independent dataset test, subsampling (K-fold cross-validation) test, and jackknife test (Chou & Zhang, 1995). However, as elucidated by a review article (Chou, 2011), among the three cross-validation methods, the jackknife test is deemed the least arbitrary and most objective because it can always yield a unique result for a given benchmark dataset, and hence has been increasingly used and widely recognized by investigators to examine the accuracy of various predictor (see, Chen & Li, 2013; Khosravian et al., 2013; Mei, 2012; Mohabatkar et al., 2013; Mohabatkar, Mohammad Beigi & Esmaeili, 2011; Wan, Mak & Kung, 2013; Zhang et al., 2008b). However, to reduce computational time, here let us adopt the 10-fold cross-validation to examine the prediction accuracy as done by many investigators for PTM sites prediction with SVM (Chang et al., 2009; Kim et al., 2004; Wong et al., 2007; Xu et al., 2013). The cross-validations were performed 50 times for different subsampling combinations, followed by averaging their outcomes. The outcomes thus obtained on the benchmark dataset 𝕊_L_ (cf. Supplemental Information S1) for the four metrics as defined in Eq. (19) are given below

(20) \begin{eqnarray*} \displaystyle \left\{\begin{array}{@{}l@{}} \displaystyle \text{Sn}=85.2\% \\ \displaystyle \text{Sp}=79.0\% \\ \displaystyle \text{Acc}=81.8\% \\ \displaystyle \text{MCC = 0.64} \end{array}\right.&&\displaystyle \end{eqnarray*}

indicating that the accuracy is quite high for all the four metrics.

### Independent dataset test

As a demonstration to show how the current predictor is used for practical application, let us use the iSNO-AAPair predictor trained by the data in 𝕊_L_ (Eq. (3)) to predict the peptides in 𝕊_T_ (cf. Eq. (4)). As mentioned in the Materials and Methods section, the independent dataset 𝕊_T_ contain 81 SNO and 100 non-SNO peptides (cf. Supplemental Information S2). To avoid the memory bias, none of the peptide in 𝕊_T_ occurs in 𝕊_L_; i.e., 𝕊_L_∩𝕊_T_ = 0̸, where the symbols ∩ and 0̸ represent “intersection” and “empty set” in the set theory, respectively. The results thus obtained are given below

(21) \begin{eqnarray*} \displaystyle \left\{\begin{array}{@{}l@{}} \displaystyle \text{Sn}=79.6\% \\ \displaystyle \text{Sp}=84.1\% \\ \displaystyle \text{Acc}=81.7\% \\ \displaystyle \text{MCC = 0.63} \end{array}\right.&&\displaystyle \end{eqnarray*}

indicating that the results obtained by the independent dataset test are quite consistent with those by the 10-fold cross-validation, particularly for the overall accuracy Acc and the Mathew’s correlation coefficient MCC.

### Comparison with the other methods

Among the existing methods for identifying the SNO sites in proteins, the web server for the method proposed in Li et al. (2011) did not work, and the method in Li et al. (2012) had no web-server at all. Therefore, the comparison was made among the following three methods: GPS-SNO (Xue et al., 2010), iSNO-PseAAC (Xu et al., 2013), and the current iSNAO-PseAAPair.

Listed in Table 1 are the corresponding results obtained by the aforementioned three methods on the independent dataset test 𝕊_T_ (cf. Supplemental Information S2), respectively. As we can see from Table 1, the overall accuracy (Acc) achieved by iSNO-AAPair was remarkably (about 30%–35%) higher than those by its counterparts GPS-SNO (Xue et al., 2010) and iSNO-PseAAC (Xu et al., 2013). Furthermore, iSNO-AAPair was also superior to its counterparts in the other three metrics (Sn, Sp, and MCC). Particularly for MCC, the rate achieved by iSNO-AAPair was significantly (about 30%–55%) higher than those by its counterparts, indicating that the high accuracy achieved by iSNO-AAPair was not an artifact but a true result, and hence it would be much more stable, consistent, and reliable in practical applications.

**Table 1:** A comparison of iSNO-AAPair with the existing prediction methods^a^ via the independent dataset test for the four different metrics (cf. Eq. (19)).

Predictor	Sn (%)	Sp (%)	Acc (%)	MCC
GPS-SNO^b^	44.5	81.0	64.7	0.28
iSNO-PseAAC^c^	50.2	75.2	62.8	0.30
iSNO-AAPair	**79.6**	**84.1**	**81.7**	**0.63**

**Notes.**

a The results for the method proposed in Li et al. (2012) and that in Li et al. (2011) were not listed because the former had no web-server and latter’s web-server did not work.

b The method proposed in Xue et al. (2010) where the threshold parameter was set at “medium” to get its highest overall accuracy.

c The method proposed in Xu et al. (2013).

Also, in practical applications, the input should be entire protein sequences. To avoid memory bias, let us randomly pick 14 protein sequences whose experimental SNO sites are known but none of them occurs in the training dataset 𝕊_L_. The sequences of such 14 proteins as well as SNO site (red) and non-SNO site (blue) are given in Supplemental Information S3. The detailed results by the three methods in identifying the SNO sites for the 14 independent proteins are given in Supplemental Information S4. For clarity, these results are summarized in Table 2 from which we can see that iSNO-AAPair outperformed iSNO-PseAAC and GPS-SNO not only in the overall accuracy Acc, but also in MCC, indicating iSNO-AAPair not only performed better but also more stable than its counterparts.

**Table 2:** A comparison of iSNO-AAPair with the existing prediction methods^a^ on the 14 independent proteins (cf. Supplemental Information S3).

Predictor	Sn (%)	Sp (%)	Acc (%)	MCC
GPS-SNO^b^	37.50	62.79	55.93	0.10
iSNO-PseAAC^c^	75.00	55.81	61.02	0.27
iSNO-AAPair	75.00	60.47	**64.41**	**0.31**

**Notes.**

a See footnote a of Table 1.

b The method proposed in Xue et al. (2010) where the threshold parameter was set at “medium” to get its highest overall accuracy.

c See footnote c of Table 1.

It is anticipated that iSNO-AAPair may become a useful vehicle for identifying the SNO sites in proteins, or at the very least play an important complementary role to the existing predictors in this area.

### Web server

For the convenience of the vast majority of biological scientists, a web-server for iSNO-AAPair was established. Here, let us give a step-by-step guide on how to use the web-server to get the desired results without the need to follow the mathematic equations that were presented just for the integrity in developing the predictor.

**Figure 5:**
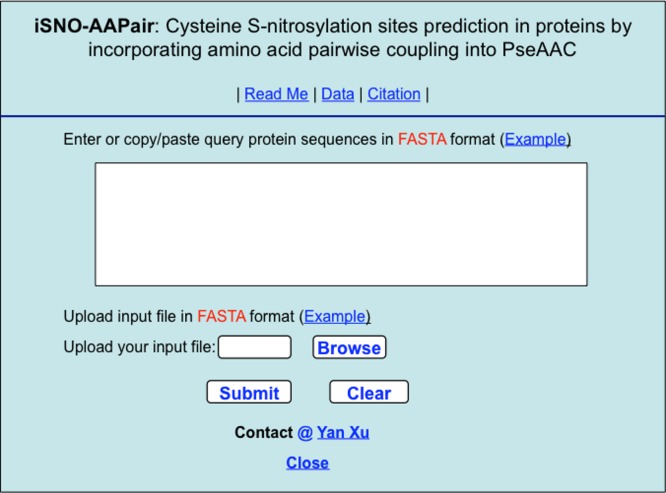
A semi-screenshot to show the top page of the iSNO-AAPair web-server. Available at http://app.aporc.org/iSNO-AAPair/.

**Step 1.** Open the web server at http://app.aporc.org/iSNO-AAPair/ and you will see the top page of the predictor on your computer screen, as shown in Fig. 5. Click on the Read Me button to see a brief introduction about iSNO-AAPair predictor and the caveat when using it.

**Step 2.** Either type or copy/paste the query protein sequences into the input box shown at the center of Fig. 5. The input sequence should be in the FASTA format. Example sequences in FASTA format can be seen by clicking on the Example button right above the input box. For more information about FASTA format, visit http://en.wikipedia.org/wiki/Fasta_format.

**Step 3.** Click on the Submit button to see the predicted result. For example, if you use the query protein sequences in the Example window as the input, after clicking the Submit button, you will see on your screen the predicted SNO site positions and the corresponding sequences segments with the form as formulated by Eq. (1). All these results are fully consistent with the experimentally verified results. It takes about a few seconds for the above computation before the predicted results appear on the computer screen; the greater number of query proteins and the longer each sequence, the more time is usually needed.

**Step 4.** As shown on the lower panel of Fig. 5, you may also choose the prediction by entering your desired input file via the “Browse” button. The input file should also be in FASTA format but can contain as many protein sequences as you want.

**Step 5.** Click on the Citation button to find the relevant papers that document the detailed development and algorithm of iSNO-AAPair.

**Step 6.** Click on the Data button to download the benchmark datasets used to train and test the iSNO-AAPair predictor.

**Caveats.** To obtain the predicted result with the anticipated success rate, the entire sequence of the query protein rather than its fragment should be used as an input. A sequence with less than 50 amino acid residues is generally deemed as a fragment.

## Supplemental Information

10.7717/peerj.171/supp-1Supplemental Information S1The learning dataset 𝕊_L_ consists of a positive dataset ${\mathbb{S}}_{\mathrm{L}}^{+}$ and a negative dataset ${\mathbb{S}}_{\mathrm{L}}^{-}$.They each contain 2300 SNO and 2300 non-SNO sites and peptide fragments derived from the 1,530 proteins. See the text of the paper for further explanation.

10.7717/peerj.171/supp-2Supplemental Information S2The independent testing dataset 𝕊_T_ consists of a positive dataset ${\mathbb{S}}_{\mathrm{T}}^{+}$ and a negative dataset ${\mathbb{S}}_{\mathrm{T}}^{-}$.The former contains 81 SNO sites and the latter 100 non-SNO sites. None of the sites and peptide fragments included here occurs in the learning dataset 𝕊_L_. See the text of the paper for further explanation.

10.7717/peerj.171/supp-3 Supplemental Information S3 The sequences of 14 independent proteins whose experimental SNO sites are known but none of them occurs in 𝕊_L_ used to train iSNO-AAPair. The SNO site is marked with red, while non-SNO site with blue.

10.7717/peerj.171/supp-4 Supplemental Information S4 Detailed results predicted by various predictors on the 14 independent proteins in Supplemental Information S3.
