# Supplementary material for: iSNO-AAPair: incorporating amino acid pairwise coupling into PseAAC for predicting cysteine S-nitrosylation sites in proteins
Source: PeerJ. 2013 Oct 3;1:e171. doi: 10.7717/peerj.171 (PMC3792191; doi:10.7717/peerj.171)
Supplement: Supplemental Information S1 — They each contain 2300 SNO and 2300 non-SNO sites and peptide fragments derived from the 1,530 proteins. See the text of the paper for further explanation. [file peerj-01-171-s001.pdf]

Online Supporting Information S1. The learning dataset $\mathbb{S}_L$ consists of a positive dataset $\mathbb{S}_L^+$ and a negative dataset $\mathbb{S}_L^-$. They each contain 2300 SNO and 2300 non-SNO sites and peptide fragments derived from the 1,530 proteins. See the text of the paper for further explanation.

Positive dataset $\mathbb{S}_L^+$ contains 2300 SNO sites and peptide fragments

Uniprot ID	Site	Sequence
Q8R164	234	ICRHLLPLVQCPTLIVHGEKD
P61979	132	QLPLESDAVECLNYQHYKGSD
P02730	201	QHSSLETQLFCEQGDGGTEGH
Q9EQ80	147	NSWLAKGLGTCTTRPIHPSRA
Q96EK6	157	KLNCYKITLECLPQNVGFYKK
Q9JK42	392	YQTIQEAGDWCVPSTEPKNTS
O70325	55	SAKDIDGHMVCLDKYRGFVCI
P27695	310	IRSKALGSDHCPITLYLALXX
P09382	43	LNLGKDSNNLCLHFNPRFNAH
Q8CHP8	193	KAVRYLQQPDCLLVGTNMDNR
P54822	266	ASLGASVHKICTDIRLLANLK
Q9BY44	119	LQLYDVKTGTCLKSFIQKKMQ
Q29RF7	1079	SKTNEKLYTVCDVALCVINSK
Q8BMF4	581	FSAIINPPQACILAIGASEDK
Q60936	265	ANAERIVSTLCKVRGAALKLG
P62245	72	VVNLTGRLNKCGVISPRFDVQ
Q8BIJ6	1005	SETADALCPRCAEVIGAKXXX
Q8R5G7	831	GLLRGDHLFLCPAPGPGPPAP
P17182	357	IGSVTESLQACKLAQSNGWGV
P78347	475	KLRKMVDQLFCKKFAEALGST
Q9D6J5	130	SPTPVSWDVMCKHLFGFVAFM
P62281	116	HKNMSVHLSPCFRDVQIGDIV
Q80UU9	75	LWVRWGRRGLCSGPGAGEESP
Q99LB2	210	ELAPKNIRVNCLAPGLIKTRF
Q9CZB0	107	ESYLMFVKSLCLGPTLIYSAK
P10605	211	EGDTPRCNKSCEAGYSPSYKE
P17405	120	ARVGSVAIKLCNLLKIAPPAV
Q9D1H8	49	SEKVRATNLNCSVIADVRHDG
Q8R404	60	PAMYQFSQYVCQQTGLEMPQL
Q9CQ75	24	LGLREIRVHLCQRSPGSQGVR
Q9D7J4	28	LGILDVENTPCARESILYGSL
A2ASS6	34176	TLRMRSHRVPCGQNTRFILNV
Q99LC3	183	MYNQGYIRKQCVDHYNEIKRL
P38647	66	VGIDLGTTNSCVAVMEGKQAK
Q924X2	448	LYGKALLHGNCYNRWFDKSFT
Q15149	1136	LKDIRLQLEACETRTVHRLRL
P18760	80	TFVKMLPDKDCRYALYDATYE
Q9D1E6	83	LLGSYPVDDGCRIHVIDHSGV
Q7TPV4	1028	PSRPRHQAQACLMLQKTLSAR
P97311	301	DLSYRLVFLACHVAPTNPRFG
Q8VCT3	151	TYRVGEGPGVCWLAPEQTAGK
Q9R112	127	RVAELNPDENCIRTDSGKEIS

Q8VDN2	459	GDASESALLKCIEVCCGSVME
O55126	88	PECLDAYNKICQEVLPKIHEG
Q9QVP4	43	QIQEFKEAFSCIDQNRDGIIC
P07310	283	WNEHLGYVLTCPSNLGTGLRG
P36536	178	LNARPMEVFMCSVLKRQGYGE
Q9H0C8	190	VISVEKTVKRCLLDTFKHTDE
Q9UDY4	175	RVSLEEIYSGCTKRMKISRKR
P62717	109	DLTTAGAVTQCYRDMGARHRA
Q924X2	608	REGRTETVRSCTNESAAFVQA
Q9D1P4	59	DFSDFLSIVGCTKGRHNSEKP
Q9R1P0	74	KIYKLNEDMACSVAGITSDAN
Q60864	461	YQKALDLDSSCKEAADGYQRC
P62192	58	KLPLVTPHTQCRLKLLKLERI
Q92600	99	RVCNALALLQCVASHPETRSA
P13639	67	TDTRKDEQERCITIKSTAISL
P63028	28	YKIREIADGLCLEVEGKMVSR
P21333	717	LRVQVQDNEGCPVEALVKDNG
P07237	312	LEFFGLKKEECPAVRLITLEE
Q3U5Q7	44	PRRFTVELPDCSLTHFVLGDA
P63000	178	VFDEAIRAVLCPPPVKKRKRK
Q64105	235	KLKSDGALVDCGTSAQKLLGL
Q8R5K2	247	SQQDAQEFLRCLMDLLHEELK
Q8R0Y8	235	YPFERMVFGACAGLIGQSASY
P84091	251	IAIDDCTFHQCVRLSKFDSER
P58252	466	RYVEPIEDVPCGNIVGLVGVD
Q8BGK2	106	HRPDPSTIEGCSQLKPDNYLL
P30046	24	RVPAGLEKRLCAAAASILGKP
Q02566	949	TAKKRKLEDECSELKKDIDDL
Q9DCD0	402	DDFFKSAVDNCQDSWRRVIST
Q99683	869	GKAADIWSLGCTIIEMATGKP
P52825	489	YGQTVATYESCSTAAFKHGRT
Q69Z37	1281	RCFKKYVELFCHLDTNLVQGK
Q91VD9	710	ISRASQTMAKCVKAVTEGAQA
P17427	330	DSEPNLLVRACNQLGQFLQHR
Q86SE5	51	IFSKYGKIVGCSVHKGYAFVQ
P27635	195	VAEKRLIPDGCGVKYIPNRGP
Q99NB1	491	LEGGDVSGALCISQAWPGMAR
O08663	135	PNGVFPKGQECEYPPTQDGRT
P13639	369	PSPVTAQKYRCELLYEGPPDD
Q9Z0N1	348	GVGTKIDPTLCRADRMVGQVL
Q9JIK5	754	MVFLKGKLGVCFDVRTEAVTE
Q9WUB3	496	GITPRRWLVLCNPGLAEVIAE
P07900	420	KVIRKNLVKKCLELFTELAED
P68366	295	QLSVAEITNACFEPANQMVKC
Q8BMF4	163	TRDVPVGSIICITVEKPQDIE
Q9ESD7	344	GARGYLKASLCVLGPGDEAPL
Q6PEB6	119	LCAAHKTPKECPAIDYTRHTL
P22315	193	IAFTQYPQYSCSTTGSSLNAI
Q5JTH9	317	MLTLLKDLLPCFPEGLVKSCS
Q61553	121	YFGGTEDRLSCFAQSVSPAEK
P00403	200	GVYYGQCSEICGANHSFMPIV
Q9D8B4	18	SYHEVPDGTQCHRKTYITTAL

Q60854	372	HHVKTNGILFCGRFSSPXXXX
Q9Y3I1	286	RLQLLPESFICKEKLGENVAN
Q99615	175	MDRALEFAPACHRFKILKAEC
Q13155	143	LSLLVLHRLLCEHFRVLSTVH
P14824	358	VSRVELKGTVCAANDFNPDAD
P35557	371	TTDCDIVRRACESVSTRAAHM
P07742	254	SAGGIGVAVSCIRATGSYIAG
Q9UHG3	258	WAVEGGNKLVCSGLLQASKSN
Q8BWF0	81	SGAKLGTVADCGVPEARAAVR
P49442	277	DTIKAALSRVCGGSVFPAAGA
Q78PY7	96	LRKKLIGKEVCFTIENKTPQG
P32921	66	AAMGEEYKAGCPPGNPTAGRN
O70468	471	VMVGQRVEFECEVSEEGAQVK
Q9CQ65	55	LILGKIKNVDCVLLARHGRQH
Q8K1I3	109	QRGYSVPTAACRSTVQMSKGQ
O35286	226	EVGYSIRFEDCSSAKTILKYM
Q91V41	40	QFTEKKFMADCPHTIGVEFGT
O88712	54	ILKDVATVAFCDAQSTQEIHE
P63104	94	EKIETELRDICNDVLSLLEKF
P50991	379	GSGKLLKITGCASPGKTVTIV
Q4VXU2	339	GGHSKGFGFVCFSSPEEATKA
Q99LC5	109	TQKQFSYTHICAGASAFGKNL
Q99KP6	114	SHALYQHDAACRVIARLTKEV
P63244	182	KLVKVWNLANCKLKTNHIGHT
Q62465	99	PGQLTLRVRACGLNFADLMGR
P21817	905	VRDDNKRLHPCLVDFHSLPEP
Q99J39	271	SNNIQGIVKECPPTETEERNR
P62701	181	DFIKFDTGNLCMVTGGANLGR
P13010	493	PNPRFQRLFQCLLHRALHPRE
P27659	253	KTHRGLRKVACIGAWHPARVA
Q9R1V6	23	CVLGTCPLARCGRAGVASLKG
O08528	438	HKAVRRLVPDCDVRFLRSEDG
P56380	6	XXXXXMALRACGLIIFRRHLI
P50544	423	QIEAAISKIFCSEAAWKVADE
Q1XH17	242	KPQTEFLMKFCLVTSRLQKIL
Q92945	176	QINKIQQDSGCKVQISPDSGG
Q8R035	82	IPLDRLSISYCRSSGPGGQNV
Q9P2E9	1323	AEFEEAQTSACRLQEELEKLR
P18031	92	ILTQGPLPNTCGHFWEMVWEQ
Q9DBF1	522	AWKQYMRRSTCTINYSTSLPL
Q8BP47	277	FRDHFFDRGYCEVTTPTLVQT
P63167	24	SEEMQQDSVECATQALEKYNI
Q8BFR5	127	TAARHYAHTDCPGHADYVKNM
Q02566	907	QDNLNDAEERCDQLIKNKIQL
P61161	221	ETVRMIKEKLCYVGYNIEQEQ
P50914	42	DQNRALVDGPCTQVRRQAMPF
Q9ESW4	407	KLLPRKLRFFCDPRKREQMLP
P23198	177	FYEERLTWHSCPEDEAQXXXX
P20073	298	TNQEIREIVRCYQSEFGRDLE
P00966	132	GNDQVRFELSCYSLAPQIKVI
Q9CXI0	244	RVLKPGGRFLCLEFGQVNDPL
P53996	159	INCSKTSEVNCYRCGESGHLA

P47955	61	LANVNIGSLICNVGAGGPAPA
Q9CXJ4	305	GSGLRKLSRQCQEQIARATGV
Q921G7	560	SIYDGPEQRFCPAGVYEFVPL
Q9D1I5	168	KPVIFLHPKDCGGVLVELEQA
P10599	69	VDDCQDVASECEVKCMPTFQF
Q9Z1Q9	1184	GCAVAVASDRCSIHLQLQGLV
P31949	13	KISSPTETERCIESLIAVFQK
Q3ULD5	131	TGIGRVSGVECMIVANDATVK
Q62234	778	NNPVKGSRFTCHGLTTAQSYI
P48039	130	GIAINRYCYICHSLKYDKLYS
P21981	669	GLHKLVVNFQCDKLKSVKGYR
P47857	170	GLVGSIDNDFCGTDMTIGTDS
Q9JI75	223	KSIWKEEPIHCTPPWYFQEXX
Q8NHW5	27	IIQLLDDYPKCFIVGADNVGS
O43175	369	QGTSLKNAGNCLSPAVIVGLL
P05109	42	DDLKKLLETECPQYIRKKGAD
Q9DB05	42	FGGSSKIEEACEIYARAANMF
O08807	151	FKSINTEVVACSVDSQFTHLA
Q76MZ3	329	ENVIMTQILPCIKELVSDANQ
Q99LC5	60	EVSCLVAGTKCDKVVQDLCKV
P10415	229	TLLSLALVGACITLGAYLGHK
Q80ZW2	146	RFVSLRDGFVCALLRFRQHVL
P15121	304	RNWRVCALLSCTSHKDYPFHE
Q6NXE6	484	VAKAALRDLGCHVELRELWTG
Q8K4Q0	362	LAERIMRSYNCTPVSSPRLPP
A2ASS6	23794	RKAYSTVATNCHKTSWKVDQL
Q9Z2W0	445	LAMHSIRETACTTGVLQTLTL
P21980	620	QNPLPVALEGCTFTVEGAGLT
O35459	101	CFQKISKDSDCRAVVVSGAGK
Q3UK37	398	KKALHQLQRQCQQELARLAGA
Q8BH61	153	VRLSVQSSPECIVGKFRMYVA
Q8BMS1	550	KDGPGFYTTRCLAPMMSEVMR
Q6ZWV3	80	ICANKYMVKSCGKDGFHIRVR
O70468	619	DYSFVPEGFACNLSAKLHFME
P23610	110	QERDARQRLVCPAAYGEPLQA
P51859	12	SRSNRQKEYKCGDLVFAKMKG
P05213	316	DPRHGKYMACCLLYRGDVVPK
Q921S7	207	SLARRTSAQNCTLATTWNRES
O00233	216	TRWAGKGLLGCNIIPLQRXXX
P29474	184	AKQAWRNAPRCVGRIQWGKLQ
Q64737	134	WRAFTNPEDACSFITSANFPA
P35579	917	TAKKQELEEICHDLEARVEEE
Q9WTX5	160	EAQVRKENQWCEEKXXXXXXX
P63330	251	HQLVMEGYNWCHDRNVVTIFS
P48200	178	CRGQTTCRGSCDSGELGRNSG
Q3U0B3	195	LEAQTHIRATCISPGLVETQF
Q14690	89	SLCEGMRILGCVKEVNELELV
O08600	193	LTRTYQNVYVCTGPLFLPRTE
Q9CQJ8	42	IHRDKYRYFACLMRARFEEHK
Q8BXV2	218	GYWRGSPGGLCSPSVEEKLEH
P53996	88	CGRGGHIAKDCKEPKREREQC
Q8CGC7	744	ERPAPAVSSTCATAEDSSVLY

Q80XN0	86	HSKGFLVFAGCLMKDKGDAGV
Q64737	237	GPNTGGMGAYCPAPQVSKDLL
P00558	99	LGKDVLFLKDCVGPEVEKACA
P06213	1261	DGGYLDQPDNCPERVTDLMRM
P23396	97	YAEKVATRGLCAIAQAESLRY
Q9BQ04	31	LFEQYGKVLECDIIKNYGFVH
P21980	143	GHFILLFNAWCPADAVYLDSE
Q99439	164	FDDATMKAGQCVIGLQMGTNK
Q60597	283	SSEKRFGLEGCEVLIPALKTI
Q8R2Y8	88	KMGKGKVAAQCSHAAVSAYKQ
Q3ULJ0	104	VIPHQFIHKICDEITGRVPEK
Q61733	345	PIRLFMELVTCGLSKNPYLSV
Q3U1J4	128	TGIIGIIDPECRMIGLRLYDG
Q9D7B6	157	LCTMEKFASYCLTEPGSGSDA
Q921H8	123	TVPLSTVNRQCSSGLQAVANI
Q96EK6	113	LIIEHKFIHSCAKRGRVEDVV
P80318	398	ERNLQDAMQVCRNVLLDPQLV
Q9NYY8	377	SKVVLDNIHGCPLRIMINILQ
Q99MR3	771	WHSARLRIFLCLGPREAPGAA
Q99NB1	271	EQEMAKEAPVCTPESMSSEDM
Q9CXY6	37	VPHIPFDFYLCEMAFPRVKPA
O89103	52	KLSAAEAQHRCNENGGNLATV
P17710	942	HQTVKELSPKCTVSFLLSEDG
P41216	242	GSDLVERGKKCGVEIISLKAL
P04075	240	KPNMVTPGHACTQKFSHEEIA
P15532	109	SKPGTIRGDFCIQVGRNIIHG
Q9H4A6	280	QLLDLDPEVECLKANTNEVLW
Q9D8N0	266	APAPEEEMDECEQALAAEPKA
P27695	65	PSGKPATLKICSWNVDGLRAW
Q3TXS7	898	QLKVLSMTETCRYQPFKPLSI
P84091	212	MKSYLSGMPECKFGMNDKIVI
Q80XN0	115	DRLRTIQLNVCNSEEVEKAVE
P78527	4045	EKNWYPRQKICYAKRKLAGAN
Q2TPA8	136	TRGTYLTSKACIPFLKKSKVG
Q8K297	364	LRALHEQEIDCQLVEAVDGKA
P61088	87	HPNVDKLGRICLDILKDKWSP
P62754	83	VRLLLSKGHSCYRPRRTGERK
P34932	34	ETIANEYSDRCTPACISFGPK
P11499	564	EESKAKFENLCKLMKEILDKK
Q8QZS1	94	IIKGAGGKAFCAGGDIKALSE
Q3U5Q7	422	MTYQRMENPSCHLVDASPSRE
Q6PB66	207	VTYQRLIAAYCNVGDIEGASK
P48962	129	SGGAAGATSLCFVYPLDFART
Q64514	614	LREGLHYTEVCGYDIASPNAG
Q07065	100	AAAAASSSASCSRRLGRALNF
P21980	10	XMAEELVLERCDLELETNGRD
Q9QYI3	58	YNYYTKAIDMCPNNASYYGNR
Q8QZR5	238	ARDRCCPRVLCVINPGNPTGQ
Q9CPR4	15	SLDPENPTKSCKSRGSNLRVH
P50570	607	YKDLRQIELACDSQEDVDSWK
Q9D7X3	171	GPNDGFLAQLCQLNDRLAKEG
Q922B1	244	TASQAAELRSCYLSSLDLLLE

Q91V80	196	GTALYYAIKNCSDKAKERGRD
Q9WVQ5	187	AHAMNEYPDSCAVLVRRHGVY
P34932	417	NSPAEEGSSDCEVFSKNHAAP
P49327	1471	RREPGGNRLRCVLLSNLSSTS
Q8BIJ6	1002	KHTSETADALCPRCAEVIGAK
Q91WU5	33	KTSADLQTNACVTRAKPVPSY
Q9JIF0	364	DFKGQLCELSCSTDYRMRXXX
Q64310	32	KQYLPHVARLCLISTFLEDGI
Q9QYB1	189	LDGDEMTLADCNLLPKLHIVK
Q922Q8	137	VAGDCLDEKQCKQCANKVLQH
Q9D517	217	RTKGFTTAVQCLRGTVAAIYD
Q8TEX9	708	VFEEVFKLLECPHLNVRKAAH
P14873	1913	EKTERTIKSPCDSGYSYETIE
P41216	55	ATRPKALKPPCDLSMQSVEIA
Q8BKC5	266	EATLQLSLKLCGDTNLNNMQR
Q8BFZ1	72	ILDAHTRKQICIVDKVTQTST
P21817	2363	VVRLLIRKPECFGPALRGEGG
Q9NVM4	262	FSKQVSSSAACHSRRFEPLTS
O70468	562	VGAKDQAVFKCEVSDENVRGV
Q9D289	149	VTAEVSSMPACKFQVMIQKLX
P30999	450	DQDNKIAIKNCDGVPALVRLL
O08529	640	ALEEAGFKLPCQLHQVIVARF
O08663	468	FEHTILLRPTCKEVVSRGDDY
Q9WV35	71	YSSGRNKTFLCYVVEVQSKGG
Q924M7	197	TEAMASALRNCFSHLMKSEKK
Q9CZU6	101	IRFRGYSIPECQKMLPKAKGG
Q8CG76	222	TRQVEAELLPCLRHFGLRFYA
P62880	149	LPGHTGYLSCCRFLDDNQIIT
Q86VP6	571	ATPYIKDLFTCTIKRLKAADI
Q8TEX9	42	LRAPAALPALCDLLASAADPQ
P62736	376	EAGPSIVHRKCFXXXXXXXXX
P62838	111	KVLLSICSLLCDPNPDDPLVP
Q9ES28	328	SPEAQQRVGGCFLSLMPQMRT
Q9EST5	27	AAVRELVLDNCKAMDGKIEGL
Q9Z2Z6	136	GIMTPGERIKCLLQIQASSGE
P62908	119	LLGGLAVRRACYGVLRFIMES
P49368	213	KIPGGIIEDSCVLRGVMINKD
Q9Z0X1	316	GGGFLGSELACALGRKSQASG
Q14684	197	DQNLKFIDPFCKIAAKTKDHT
P06213	1165	FVHRDLAARNCMVAHDFTVKI
O70433	254	WHNDCFNCKKCSLSLVGRGFL
Q9Z2I0	329	NLTRPQLVALCKLLELQSIGT
Q9GZT4	113	YIVVPQTAPDCKKLAIQAYGA
P80316	253	VVDAKIAILTCPFEPPKPKTK
P47738	321	FALFFNQGQCCCAGSRTFVQE
Q99836	216	SIASELIEKRCRRMVVVVSDD
P50462	171	DKDGELYCKVCYAKNFGPTGI
P50544	216	VASGQALAAFCLTEPSSGSDV
P05125	129	AGPRSLRRSSCFGGRIDRIGA
P31749	296	GHIKITDFGLCKEGIKDGATM
P14618	474	HLYRGIFPVLCKDPVQEAWAE
P06151	163	FPKNRVIGSGCNLDSARFRYL

O60610	1227	GPRQANRKAGCAVTSLLASEL
P17742	115	PNTNGSQFFICTAKTEWLDGK
Q8BIJ6	91	SDMELEIQQKCGFSELYSWQR
Q8VCT3	181	QAVLNRAFFPCFDTPAVKCTY
Q99MR8	591	VLGDLSSEDGCTYLKSSINGV
P42704	930	SARLQWFCDRCVANNQVETLE
Q99LB2	89	GEGLSVTGIVCHVGKAEDREK
Q9D0K2	456	KGNAHKIMEKCTLPLTGKQCV
Q9CPR4	144	GRINPYMSSPCHIEMILTEKE
Q02248	213	QNTNDVETARCTAGTLHNLSH
Q9CQJ8	103	RYECYKVPEWCLDYWHPSEKA
Q9CPP0	41	PVTMDSFFFGCELSGHTRSFT
Q60864	471	CKEAADGYQRCMMAQYNRHDS
Q16665	520	SPEPNSPSEYCFYVDSDMVNE
P06801	468	FPGVALGVVACGLRHIDDKVF
Q8BGQ7	903	DNEAGKITCLCQVPQNAANRG
P21333	810	GQGDVSIGIKCAPGVVGPAEA
O08749	477	AALALEYGASCEDIARVCHAH
Q9R0Y5	187	GTVDTVFSEVCTYLDSLKXXX
P21817	3635	SKQRRRAVVACFRMTPLYNLP
O35855	229	VRAWIGGVGDCKLGGNYGPTV
P49327	634	GLSWEECKQRCPPGVVPACHN
Q8QZS1	270	FEEHMDKINSCFSANTVEQII
P20618	224	DVYTGDALRICIVTKEGIREE
Q9JHR7	414	EGPQEWVFQECKDLNAVAFRF
Q9Z0N1	101	LDDPSCPRPECYRSCGSSTPD
Q15631	225	RGFNKETAAACVEKXXXXXXX
P05455	232	EMKSLEEKIGCLLKFSGDLDD
Q14166	361	RPGVLLNQFPCENLLTVKDCL
Q9WTP6	208	VEYYRKRGIHCAIDASQTPDI
Q8K298	970	PFLSPLEGHICLKISCQVNSA
P80314	395	EAERSLHDALCVLAQTVKDPR
Q9WTP6	232	SILAAFSKATCKDLVMFIXXX
Q6PDY2	169	RAEYTEASGPCVLTPHRDNLH
Q99J39	447	DSSLKGLTSSCGLMVNYRYYL
Q1XH17	144	PQQKMQLQEACMRKEKTVAVL
O15519	254	YKMKSKPLGICLIIDCIGNET
O08528	386	QIVSTRSASLCAATLAAVLWR
P63037	302	GQIVKHGDIKCVLNEGMPIYR
P63318	296	YNVPVADADNCSLLQKFEACN
O70433	71	WHEGCFHCSRCGSSLVDKPFA
O08997	41	FNIDLPNKKVCIDSEHSSDTL
P47964	48	KFVRDMIREVCGFAPYERRAM
Q8R127	98	MAKQAKLVLNCVGPYRFYGEP
Q8BK08	78	ETARWITVGNCLHKTAVLAGT
P07814	105	FTSTINELNHCLSLRTYLVGN
Q9D6Y7	107	HTRNPTYKEVCSEKTGHAEVV
Q6ZPY7	1187	LDPHTSHSWLCDGRLLCLHDP
O88544	70	VISRQLLTDFCTHLPNLPDST
P62258	97	QMVETELKLICCDILDVLDKH
P19096	893	RVIFPGTGYLCLVWKTLARSL
Q9JK42	195	PKHIGSIDPNCSVSDVVKDAY

Q91VM9	67	PFHDIPLKADCKEEHDIPRKK
Q8R1H0	68	WRRSEGLPSECRSVTDXXXXX
Q9CQT1	199	HEMGRLEHTFCTETRPYNQGA
P27546	636	ETPGSQPSEPCSGVSRQEEAK
P63028	172	FFKDGLEMEKCXXXXXXXXXX
Q9WV34	310	LELTPTSGTLCGSLSGKKKKR
P23229	86	RANRTGGLYSCDITARGPCTR
Q8BRK8	490	RSGSSTPQRSCSAAGLHRARS
Q61151	106	LVDYITISRGCLTEQTYPEVV
P16125	294	IENEVFLSLPCILNARGLTSV
Q8BH86	252	APLAFASPPGCMVMVPKDTAS
Q9QYB1	100	KIEEFLEEVLCPPKYLKLSPK
Q9DC61	265	VGVEHEHLVECARKYLVGAEP
P62820	126	NVNKLLVGNKCDLTTKKVVDY
Q9DB77	192	YKNALANPLYCPDYRMGKITS
O35215	57	TLLMNKSTEPCAHLLVSSIGV
P21980	98	WTATVVDQQDCTLSLQLTTPA
Q8BVE3	73	QEMLQTEGSQCAKTFINLMTH
Q9CR51	69	EAAALGSHGSCSSEVEKETRE
Q9DBB5	52	RSLPGATAAECASNLKKIYTV
P35247	35	TYSHRTMPSACTLVMCSSVES
O00232	255	KHYRAIYDTPCIQAESEKWQQ
P53395	279	AALKIPHFGYCDEIDLTQLVK
P23229	541	PSGICLQVKSCFEYTANPAGY
P00533	190	FQNHLGSCQKCDPSCPNGSCW
Q92945	436	EMTFSIPTHKCGLVIGRGGEN
Q99MK8	439	LQRDVNRRLGCLGRGAQEVKE
P70695	282	PNGKLRLLYECNPVAYIIEQA
Q09666	2806	DVSGPKVDVECPDVNIEGPEG
P84086	90	AEEKAALEQPCEGSLTRPKKA
P04406	247	TANVSVVDLTCRLEKPAKYDD
Q9QVP4	164	LAGNIDYKSLCYIITHGDEKE
Q8K1M6	351	QLITKFATEYCNTIEGTAKYI
Q9DCZ1	316	LDILGGLRSTCTYVGAAKLKE
P30050	17	PNEIKVVYLRCTGGEVGATSA
P47754	157	KVDGQQTIIACIESHQFQAKN
Q9DCS1	41	SALAKLLLAGCSLLRIPASAS
P30086	168	YELRAPVAGTCYQAEWDDYVP
Q7Z6M4	219	VQQVTKILHSCPSVLREDLGQ
P45880	47	LVKLDVKTKSCSGVEFSTSGS
P46782	155	RRVNQAIWLLCTGAREAAFRN
Q8K2B3	536	FRVGSVLQEGCEKISQLYGDL
Q8K2B3	357	MTLEIREGRGCGPEKDHVYLQ
Q91YT0	206	FVVRGAGAYICGEETALIESI
P70698	30	ASSVGTILKSCGLHVTSIKID
Q91VD9	727	GAQAVEEPSICXXXXXXXXXX
Q96EK6	128	RVEDVVVSDECRGKQLGKLLL
Q64737	93	VGDLTSAGVRCFGPTAQAAQL
O55143	669	ELSPSAQRDACLNARCFARVE
Q05816	127	IVECVMNNATCTRVYEKVQXX
P52480	31	AMADTFLEHMCRLDIDSAPIT
Q9R062	89	ELGITLTKLHCWSLTQYSKCV

P60603	15	VGPYGQSQPSCFDRVKMGFVM
Q62407	1697	LERMARKPTVCESETRTYMRQ
Q9CZW5	179	LQKWKEVAQDCTKAVELNPKY
O08663	380	GKGVVHDDMECSHYMKNFDVG
Q9JII6	260	LRWQVQRKVICIPKSINPSRI
P21266	39	DTSYEEKRYTCGEAPDYDRSQ
P02463	1460	RHSQTTDDPLCPPGTKILYHG
Q8VE95	132	SDSESPRLSYCGGGEALAIPF
O70370	335	NKNHCGIASYCSYPEIXXXXX
Q9Y272	11	MKLAAMIKKMCPSDSELSIPA
Q9D051	263	AAVLSKEGIECEVINLRTIRP
Q80XN0	288	DEKIAKMETYCNSGSTDTSSV
P36873	158	LWKTFTDCFNCLPIAAIVDEK
P47809	244	LLDRSGNIKLCDFGISGQLVD
P51637	19	LEARIIKDIHCKEIDLVNRDP
Q9Z1W9	249	KVRKTFVGTPCWMAPEVMEQV
Q6NVF9	159	LHGQSPVVTPCNKQFLSQFEM
Q60597	904	DPHKVKRLLFCTGKVYYDLTR
O75828	227	KADRILVNACCPGPVKTDMDG
Q04760	61	RVLGMTLIQKCDFPIMKFSLY
Q9UHB9	562	KPLVERFETFCLDPSLVTKQA
Q2TPA8	218	MLGGSGVENQCRKVDIIADAA
P23242	260	TTGPLSPSKDCGSPKYAYFNG
Q9QVP4	53	CIDQNRDGIICKSDLKETYSQ
Q8VHX6	713	LKLYAQDADGCPIDIKVIPNG
O75153	753	LKDAAAFLLSCQIPGLVKDCM
Q60936	403	DVLRRELTLECDYQREAAYAK
P58059	49	ISRRRYYEKPCRRRQRESYET
P50995	294	AIKGVGTDEACLIEILASRSN
Q8VBW6	153	LWNSQIPLLICRTYGLVGYMR
Q8BZF8	165	ISKTIEEYAICPDLRIDLSRL
P06801	47	QLNIHGLLPPCIISQELQVLR
P28663	103	KKADPQEAINCLNAAIDIYTD
P07744	457	TYRKLLEGEECRMSGECKSAV
P55264	105	EPHKAATFFGCIGIDKFGEIL
Q8VEM8	131	TLIGYSMQGLCKFGFYEVFKA
Q9ESW4	34	WGGHWLYGKHCDNLLRRAACQ
P48507	72	LVREFPDVLECTVSHAVEKIN
P18669	153	ADLTEDQLPSCESLKDTIARA
P53621	245	NESKAWEVDTCRGHYNNVSCA
Q9WUA3	410	LPDEKIVKSNCNVAVINVGAP
Q8K0D5	723	YTMEYCRYQPCSPSTQEELIN
O70305	447	EEKYTAVQRNCSDREGHGPNT
P17844	200	YCRACRLKSTCIYGGAPKGPQ
O43175	281	ALVDHENVISCPHLGASTKEA
Q96EL3	21	RPVKQVRVQFCPFEKNVESTR
P05202	212	PEQSVLLLHACAHNPTGVDPR
O14983	349	VETLGCTSVICSDKTGTLTTN
Q99MR8	58	VLIANRGEIACRVIRTAKKMG
P84091	246	SGKQSIAIDDCTFHQCVRLSK
P11172	174	QAHGIRLHSVCTLSKMLEILE
Q9P2E9	892	KRLDEVSRELCHTQSSHASLR

P35579	569	PKQLKDKADFCIIHYAGKVDY
Q8BVF2	128	LHLYKQGIPLCSLINHHLSGL
Q8CEI1	110	GLRIFTSVPKCXXXXXXXXXX
Q9CQR2	56	FNGQFKTYGICGAIRRMGESD
P62317	63	LGRVKAFDRHCNMVLENVKEM
P35486	261	GLRVDGMDILCVREATKFAAA
Q8BGD5	582	LAHFRDRGQFCLTYESAMTRL
Q9DB43	230	DDRTAGIHGDCDDDKYRRRPA
Q6NZJ6	825	FSVAYANMCRCLMALKVPTTE
P78527	3347	IANALSSEPACLAEIEEDKAR
Q8K0G5	198	TAGRWSPHHNCTQVATASDTT
Q8VCW8	503	DIALMDEQGFCKIVGRSKDMI
Q15369	11	MDGEEKTYGGCEGPDAMYVKL
P60766	157	RDLKAVKYVECSALTQKGLKN
P02463	1493	HGQDLGTAGSCLRKFSTMPFL
P45983	116	IVMELMDANLCQVIQMELDHE
B1AR13	81	VCGRSKNQPFCDGSHFFQRTG
P53811	187	WKKELANTPDCPRMCAYKLVT
Q922B2	267	CICADFEKVFCIGPVFRAEDS
P63005	252	PNQDGTLIASCSNDQTVRVWV
P14618	423	AVGAVEASFKCCSGAIIVLTK
P33992	482	AGITTTLNSRCSVLAAANSVF
P63254	7	XXXXMPKCPKCDKEVYFAERV
Q62523	376	QRQSVAVNESCGKCNQPLARA
Q14103	226	DNKTNKRRGFCFITFKEEEPV
Q9Z110	674	LRTEYGDLEVCIEVVDSVQEA
Q924M7	29	GKVGSKSEVACLLASSDPLAQ
Q8BTM8	8	XXXMSSSHSRCGQSAAVASPG
Q8BWF0	490	VNEGLISSVECPFGGVKQSGL
Q3LXA3	404	ALDRAAGDGDCGTTHSRAARA
Q9R0P3	206	ESKWKAYDATCLVKAYSGSQI
Q9WTL7	171	GSAKDLAILQCHGELDPMVPV
Q61316	167	MDATQIAGLNCLRLMNETTAV
Q91V76	249	HFYEMKAPLVCLPVFVSKDPG
P62879	317	VLAGHDNRVSCLGVTDDGMAV
P29474	853	GWVRDPRLPPCTLRQALTFFL
Q99JI6	141	GQNLARQWNNCAFLESSAKSK
Q9R0P3	158	HSMGGHGALICALKNPGKYRS
Q15185	75	KHKRTDRSILCCLRKGESGQS
P36552	362	PSYVPIVKKHCDDSYTPRDKL
P99028	51	CVKARERLELCDNRVSSRSQT
P36873	155	NIKLWKTFTDCFNCLPIAAIV
Q6PGB6	79	QKRLYIMTLGCLAPYRRLGIG
P80318	372	FITDCKDPKACTILLRGASKE
O09131	236	NLYLQDSPEACDYGLXXXXXX
P39687	123	ENLKSLDLFNCEVTNLNDYRE
Q8BGH2	445	GNIARLELNYCIPMGVQGGDR
Q9WTP7	85	ALHELKTLTQCSWLLDGFPRT
Q99JB8	402	KMSEEDEQGWCQGQLQSGRIG
Q8BGC4	218	EKAAFLKSIGCDRPINYRTEP
P54071	402	IRFAQTLEKVCVQTVESGAMT
Q501J6	219	IRDLERGVEICIATPGRLIDF

P62829	28	LGLPVGAVINCADNTGAKNLY
Q99MN9	367	VGNQPNVASGCLDINSSVKGA
O00429	367	TSELCGGARICYIFHETFGRT
Q06203	348	KCGLPYVEVLCKNRYVGRTFI
Q9QZ73	29	TQSSEKTAVSCLSQNDWKLDV
Q9Y3B4	83	ACDHLSGFNVCNRYLVVLYYN
O55229	72	RRARPEELSVCPVSGGLSNLL
Q8CDN6	149	NESDEHGFDNCLRKDMSFLES
Q9DCM0	98	TGVLRSLLPGCQSVISRLSGA
Q91YE3	58	RQDWKKHKLVCQGGEAPRAQP
P13639	651	WDVAEARKIWCFGPDGTGPNI
P62736	219	EIVRDIKEKLCYVALDFENEM
P50462	79	KGIGFGQGAGCLSTDTGEHLG
P67936	154	EERAEVSELKCGDLEEELKNV
P18760	39	VKKRKKAVLFCLSEDKKNIIL
P55264	352	AASVIIRRTGCTFPEKPDFHX
O00429	644	RKLSAREQRDCEVIERLIKSY
Q13557	373	NGDFEAYTKICDPGLTAFEPE
Q9WVL0	16	PILYSYFRSSCSWRVRIALAL
P67775	266	VVTIFSAPNYCYRCGNQAAIM
Q7L5N1	143	PSDIHVHKQVCEIIESPLFLK
Q920E5	332	YNRLKSLIEQCSAPLPPSIFM
Q99MK8	72	KLGYLLFRDFCLNHLEEAKPL
Q9DC69	86	MGSQVIIPYRCDVYDIMHLRL
Q9QZD9	76	HVLTGSADNSCRLWDCETGKQ
Q8K0W9	66	KVIYDKDQFMCGETVPAPSTN
P05201	160	AAGFKDIRPYCYWDAEKRGLD
Q9Z1W9	536	KTLTFKLASGCDGSEIPDEVK
P13010	249	KIERHSIHWPCRLTIGSNLSI
Q8VE95	109	LCGRERNFLRCEDRPVVFTHL
P27635	71	SSEALEAARICANKYMVKSCG
P97821	447	YFRIRRGTDECAIESIAVAAI
P62983	144	SHFDRHYCGKCCLTYCFNKPE
P98170	327	WEQHAKWYPGCKYLLEQKGQE
P40936	231	VVEKAIQDAGCQVLKCNCVSL
Q921G7	591	NAQNCVHCKTCDIKDPSQNIN
P78527	2342	ERKNILEESLCELVAKQLKQH
Q9DCL9	63	KAAISNKITSCIFQLLQEAGI
O55143	377	FILDKVEGDTCSLNEFSITGS
Q9ER88	379	GRKELRFLSNCNPEQLERLCA
Q9DBC0	220	LGIPTTRAGACVTSESTVMRD
Q9JLI6	304	GKAADLVSENCETYEAHMRDI
Q07076	338	GEGRLGTDESCFNMILATRSF
Q8BKZ9	170	TQPSPQPQIPCPARKEHKGTA
Q96EP5	124	KIFVGGIPHNCGETELREYFK
Q791V5	296	FRKVPCGKTYCYDLRMLIXXX
O08573	73	PRFEEGGYVVCNTKQNGQWGP
Q03265	244	DGTDEKKKLYCIYVAIGQKRS
P70698	216	GLGLSPDLVVCRCSNPLDTSV
P13010	296	KEDIQKETVYCLNDDDETEVL
Q9WUB3	172	YEFGIFNQKICGGWQMEEADD
P23229	131	SQGPGGKVVTCAHRYEKRQHV

Q9Z0S1	249	ASPGCKKWDTCAPEVILHAVG
Q61425	99	GDEFVEKTLSCLSTSTDAASV
Q60759	176	PGLAKGELLGCFGLTEPNHGS
Q61234	220	SLKMAYVSRRCTPTDPEPRYL
Q924D0	130	GRDVSGVVMECGLDVKYFQPG
Q8R0F8	68	EGSPVLMPAYCRNLHHEVELG
Q9D6R2	127	TFDLYANVRPCVSIEGYKTPY
P80316	493	KESNPALGIDCLHKGSNDMQY
Q9CXK8	36	LLVDRPDGTYCFRLHNDRVYY
Q9Y570	312	FRGLSNLFLSCPIPKLLLLAG
P05202	382	QHITDQIGMFCFTGLKPEQVE
P41250	471	CADRSCYDLSCHARATKVPLV
P14206	148	SYVNLPTIALCNTDSPLRYVD
P17918	135	QLGIPEQEYSCVIKMPSGEFA
Q91WD5	146	FDRLDYVSMMCNEQAYSIAVE
P10809	447	VLGGGCALLRCIPALDSLTPA
P12814	480	PSVNARCQKICDQWDNLGALT
Q8BH59	375	GELMYKNSFDCFKKVLRYEGF
P35235	104	DVIELKYPLNCADPTSERWFH
O08528	375	QEDCVATHRICQIVSTRSASL
Q00610	151	MFDRHSSLAGCQIINYRTDAK
P70349	84	LGHLMIVGKKCAADLGLKRGY
Q8CC88	857	EADKAPTNVTCILKTLVENGE
Q8BGK2	23	VGDALGYGNICRENSVLGSIQ
Q791V5	79	GLFTGLTPRLCSGVLGTVVHG
P43235	139	PVKNQGQCGSCWAFSSVGALE
Q6PE15	116	IGSSDGNLAECTVGKWRKDVL
P62962	128	GVHGGLINKKCYEMASHLRRS
Q99798	592	LQILIKVKGKCTTDHISAAGP
Q9H2U2	180	VCEIGSKILSCGEVIHVKILG
Q8QZS1	44	AAEVLLERRGCGGVITLNRPK
Q3U186	576	GMKLLGITPVCRMXXXXXXXX
O88342	225	IYDGKTGEKVCALGESKAHDG
Q9CPU4	56	TDPENGHMFNCIQRAHQNTLE
Q9CR68	51	PVLDVKRPFLCRESLSGQAAA
Q9DAK9	39	AEPSGDPAKECKEIVRGYKWA
O08573	258	DATRFHINLRCGGDIAFHLNP
P09541	74	TGEMKITYGQCGDVLRALGQN
Q99JY9	34	NTEPQFIIPSCIAIKESAKVG
Q8BMP6	484	DEIVPVYRRDCHEEVYAGSHQ
Q78PY7	228	LVTVMLSGIKCPTFRRETDGS
Q9D172	242	NKVVTTPAFMCETALHHIHDG
Q99J09	172	SYRAHAGQVTCVAASPHKDSV
P07814	856	PKAKINEAVECLLSLKAQYKE
P70460	332	TTSEAHPSTPCSSDDSDLERV
Q80X90	991	ILSPSRKVVPCLVAPVAGREC
P54886	606	KVTRLVRDSKCEYPAACNALE
Q9D7N6	138	LPTEETMSSTCLKSTGELVVQ
Q9Z0X1	440	SNIWVAGDAACFYDIKLGRRR
Q14152	478	ERAIVDAARHCDLQVRIDHTS
Q6PAR5	71	TSAEASPAECCQHAKILEDTQ
Q9BQG0	1031	TGPVRPRHQACLLLQKTLSMR

Q2TPA8	166	NLNPLWFKQHCAYTIAKYGMS
P14174	81	AQNRSYSKLLCGLLAERLRIS
Q9ER72	488	LHTGHLTIAGCKMSKSLKNFI
P15170	453	RPRFVKQDQVCIARLRTAGTI
Q9JKV1	88	DDCEFKRVPQCPSGRVYVLKF
P62918	195	AYHKYKAKRNCWPRVRGVAMN
Q8R1B4	79	NAMKIRDVTKCLEEFELLGKA
Q8CI94	808	WTKKVIRNIACSGKFSSDRTI
Q6PB66	1321	ELRDNDKVYSCSMKSYALDKD
Q99LX0	46	AGLAGKDPVQCSRDVMICPDT
Q9JI91	187	HTSWKDGLGLCALIHRHRPDL
Q6PB66	129	SNQALLLLRSCGSLLPELSLA
Q80X85	152	YRIFHEALKNCEPVIGLVPIL
O88844	73	AIKKYNVGVKCATITPDEKRV
Q9JMA1	257	FGVEFETTMKCTESEEEEVTK
P51174	351	ICVTRAFVDSCLQLHETKRLD
Q7TNG5	395	THPSRAQFVTCGQDKLVHLWS
Q7TMK9	96	SHVQNKSAFLCGVMKTYRQRE
P00533	470	DVIISGNKNLCYANTINWKKL
Q9R190	209	AVGTFARALDCSSSIRQPSLH
Q9DCZ1	186	IKVGVGPGSVCTTRTKTGVGY
Q09161	477	DIVPPTFSALCPANPTCIYKY
P30050	141	EILGTAQSVGCNVDGRHPHDI
Q8BFP9	419	YKANHEADDWCVPSREPKDMT
P18031	215	SPEHGPVVVHCSAGIGRSGTF
P41216	133	SYKEVAELAECIGSGLIQKGF
P61247	201	DSIGKDIEKACQSIYPLHDVF
O08992	119	IKQGIREVILCKDQDGKIGLR
P49915	456	RVICAEEPYICKDFPETNNIL
P12658	100	LLFRCQQLKSCEEFMKTWRKY
Q9GZT3	48	HFAQFGHVRRCILPFDKETGF
P04271	85	MAFVAMVTTACHEFFEHEXXX
Q6ZQ73	240	SPAAIRTLIQCLGSVGRQAGH
Q1XH17	313	VVSSSGRRVECSDQKAPPAGE
Q9EST5	124	CLKSLDLFGCCVTNRSDYRET
Q92769	262	MYQPSAVVLQCGADSLSGDRL
Q9CQS2	28	KKFDPMGQQTCSAHPARFSPD
P05201	391	YLLPSGRINMCGLTTKNLDYV
O14983	344	RSLPSVETLGCTSVICSDKTG
P60900	161	VYKCDPAGYYCGFKATAAGVK
P62932	687	KTDPIRLTSMCQPQEKARESL
O60443	156	QVLEGRNEVLCVLTQKITTMQ
P97822	87	SGGLEVLAEKCPNLTYLNLSG
Q9NYY3	26	KMCEQALGKGCGADSKKKRPP
Q99KN2	245	SGSDPSWKCICTLSGFHTRTI
A2APY7	98	RDFPLALDIGCGRGYIAQHLD
Q91ZA3	107	VHVKMADEAVCVGPAPTSKSY
Q922B1	184	SLLGGGGVDGCIHRAAGSLLT
P21817	3193	VEKLRPALGECLARLAAAMPV
P60981	147	ANGPEDLNRACIAEKLGGSLI
P35285	9	XXMALRELKVCLLGDTGVGKS
P12814	332	LHKPPKVQEKCQLEINFNTLQ

P24270	377	GPNYLQIPVNCPYRARVANYQ
Q8BJW6	377	PDGEHILTATCAPRLRVNNGY
O08709	47	FSHPRDFTPVCTTELGRAAKL
Q60597	395	MGKTKAEQFYCGDTEGKKVMS
P21980	230	VGRVVSGMVNCNDDQGVLLGR
Q8BTM8	2199	EIVEGENHTYCIRFVPAEMGM
Q9DCS3	345	IRQGRLTAPSCSEVPLQGYQQ
P16546	1622	MGNSLIERGACAGSEDAVKAR
Q6P3A8	380	EPFYIPDKWKCYDALRKMINY
P61081	47	DINELNLPKTCDISFSDPDDL
P45376	81	QDLFIVSKLWCTFHDKSMVKG
Q8R086	300	STARWAGARLCDVLAQAGHRL
Q91YT0	286	FNISGHVNHPCTVEEEMSVPL
Q61184	317	KEYKKIIVSLCTAKMFFVESS
P17563	31	GPREEIVYLPCIYRNTGTEAP
P63085	159	KPSNLLLNTTCDLKICDFGLA
Q7TNS2	13	ESELGRKWDRCMADTVVKLGT
P49458	48	LCVKVTDDLVCLVYKTDQAQD
Q9JLJ2	267	GKSPLIIFSDCNMENAVKGAL
P98170	213	CGGKLKNWEPCDRAWSEHRRH
P55072	105	RLGDVISIQPCPDVKYGKRIH
Q8K1X1	515	LLHKELSVHSCEVKGIEWTSL
Q8R3F5	270	MLPVSGGFHTCLMEPAVDPLM
O88342	325	VIKGHSKSIQCLTVHRNGGKS
P17844	234	TPGRLIDFLECGKTNLRRTTY
O70546	1303	KYCLLRTLKQCQTLREALIAA
P37268	6	XXXXXMEFVKCLGHPEEFYNL
Q68FD5	459	EKWLKEDKLECSEELGDLVKS
Q8JZN5	617	RQILEKRAYICAHPLDRASXX
Q9D883	169	EMGECTRGGFCNFMHLKPISR
Q9Z1P6	55	PSHKLSNNYYCTRDGRREVVP
Q6P8J7	397	VIDGVNYLVDCEKKLERGQDI
P12277	254	GNMKEVFTRFCTGLTQIETLF
P67984	25	KKQVLKFTLDCTHPVEDGIMD
Q8C7B8	722	FILGHLETRQCELASTMLTAA
P34914	512	MSKNMEKWIPCLKRGHIEDCG
P80318	455	EVIPRTLIQNCGASTIRLLTS
Q8BIJ6	819	ENEKDPKRRSCQTALAEILDV
O88342	382	TVNESEQLVSCSMDDTVRYTN
Q9D7S9	20	PKAPPPSLTDCIGTVDSRAES
Q924X2	462	WFDKSFTLISCKNGLLGLNTE
Q9CR76	156	TFWGWRQDTYCAVSDMIPLSE
P83731	6	XXXXXMKVELCSFSGYKIYPG
P36405	118	AELLEEEKLSCVPVLIFANKQ
Q91VD9	64	EKVGMQIPRFCYHERLSVAGN
Q12879	320	FSYIPEAKASCYGQMERPEVP
Q922B1	212	NCETGKAKITCGYRLPAKYVI
P62070	55	PTIEDSYTKQCVIDDRAARLD
Q921M4	981	RERPGLGSNPCIPFFYRADEN
P09651	43	HFEQWGTLTDCVVMRDPNTKR
O55222	42	DHGFSPLHWACREGRSAVVEM
Q9WUQ2	161	FSNEPLQKVVCFNHDNTLLAT

P01111	118	DVPMVLVGNKCDLPTRTVDTK
Q924X2	548	KALADDVELYCFQFLPFGKGL
Q80UJ7	322	VRVRKADNPQCLLGDFVTEFL
O88844	269	MKSEGGFIWACKNYDGDVQSD
P36873	245	FLHKHDLDLICRAHQVVEDGY
O60716	394	SNAAAYLQHLCYRNDKVKTDV
Q9CQS4	127	AHPCIVLRRQCQVNYHARHYH
Q8R1H0	34	VNKHPDPTTLCLIAAEAGLTE
Q80X90	1434	VKIAGPGLSSCVRACIPQSFT
P35754	8	XXXMAQEFVNCKIQPGKVVVF
Q9Z2Z6	58	QPPMYSGTLDCFRKTLMREGI
Q99JX7	587	SGMNLEWSQKCLQDNNWDYTR
O15523	315	GQQIRDLERGCHLLVATPGRL
P30416	328	RLASHLNLAMCHLKLQAFSAA
Q91VI7	80	LVLQGLQNPTCKIQKLSLQNC
Q9Z2W0	36	SPSPFHVVAECRSRLLQAGFR
P00533	291	PEGKYSFGATCVKKCPRNYVV
Q9CZ42	78	QDGRIGIVGGCQEYTGAPYFA
O00170	208	AAAKYYDAIACLKNLQMKEQP
P54071	336	SLGLMTSVLVCPDGKTIEAEA
Q9Y696	234	AYSRDEFTNTCPSDKEVEIAY
Q8BFR5	147	MITGTAPLDGCILVVAANDGP
P04083	324	FYQKMYGISLCQAILDETKGD
Q8K1M6	367	TAKYIETSELCGGARICYIFH
P61750	62	NVETVEYKNICFTVWDVGGQD
Q3U0V1	297	IGDPYKVQQACEMVMDILRER
Q11011	67	AEVSPINYSLCLKPDLLDFTF
P50136	175	DYPLELFMSQCYGNVNDPGKG
Q9CWJ9	325	FGDFVALSDICDVPTAKIISR
P21333	1260	EPAVDTSGVQCYGPGIEGQGV
Q8BH59	487	LFGLYKGAKACFLRDIPFSAI
Q3TC72	214	WLLGKTFDTFCPLGPALVTKD
P49312	175	QKYHTVNGHNCEVRKALSKQE
O08756	58	ESQAKKLGESCIFAPANVTSE
P34914	312	EIEEYAMELLCKEMVTFLDKL
Q6NS46	1229	VDPDVPRAFLCLSLIGPYRLE
Q14444	226	DLLEGKEKPVCGTTYKVLKEI
Q8BVA5	185	PNGKFATPFLCQFRYLLYATS
P11926	360	YYSSSIWGPTCDGLDRIVERC
Q9C0B1	104	VSRILIGNPGCTYKYLNTRLF
Q9EP69	84	VITKKMKVGECFNHAVWRATD
P09936	220	GEVRFSAVALCKAAXXXXXXX
P04350	239	VSATMSGVTTCLRFPGQLNAD
P49458	39	LKYRHSDGNLCVKVTDDLVCL
O08709	201	PTLSEEEAKQCFPKGVFTKEL
P50462	58	TVAAHESEIYCKVCYGRRYGP
P07310	146	SIKGYTLPPHCSRGERRAVEK
Q6ZWV3	49	KKAKVDEFPLCGHMVSDEYEQ
Q9QY76	41	KLGNPTDRNVCFKVKTTVPRR
P50570	27	QDAFSSIGQSCHLDLPQIAVV
P97379	73	HKVLSLNFSECHTKIRHVDAH
Q91XE4	175	SVESISKNGICLEMGPQPQGV

Q5XJY5	286	MKIEEKITLTCGRDGGLQNME
Q06203	339	TPAALAYAGKCGLPYVEVLCK
O88545	296	MAYLGTITKTCNTMNQFVNKF
Q01853	691	GFSGADLTEICQRACKLAIRE
Q9JIK5	450	NPQTLLFSATCPHWVFNVAKK
Q16543	308	YESLPEELQKCFDVKDVQMLQ
P36578	250	LAPGGHVGRFCIWTESAFRKL
Q9D7H3	28	QILRVSTALSCLLGLPLRVQK
Q01853	209	NEVGYDDIGGCRKQLAQIKEM
Q05586	744	AVLEFEASQKCDLVTTGELFF
P06151	84	TPKIVSSKDYCVTANSKLVII
Q9CZR8	63	RRKTGYSFVNCKKALETCGGD
Q62167	468	EDFLYHEGYACTSIHGDRSQR
P21980	370	TPQEKSEGTYCCGPVPVRAIK
Q9Z1Z0	303	PTNPPGATSSCQKAMFQCGLL
P14618	424	VGAVEASFKCCSGAIIVLTKS
O55222	422	TIPPGISPHVCKLMKICMNED
P62701	41	PSTGPHKLRECLPLIIFLRNR
P20618	82	YKLTDKTVIGCSGFHGDCLTL
Q01518	93	ERALLVTASQCQQPAENKLSD
P43897	240	VLGKYGALVICETSEQKTNLE
P61978	185	QTTIKLFQECCPHSTDRVVLI
Q8R5A6	150	VKSVSESHTPCPSESTGDTVP
Q9CPY7	445	FEHYTRQVIDCQLADVNNLGK
O70325	195	EPQVIEKDLPCYLXXXXXXXX
Q60854	102	TANRLFGDKTCDLLASFKDSC
Q8R086	265	PKHEVTVTLQCAGNRRSEMSK
Q9JHW2	146	DSFSTFDTPYCKVGLGICYDM
Q3U5Q7	243	ARAVLDLVDQCPKEVQKGKFQ
O55126	103	PKIHEGKQYPCTLVGTWNTWY
Q9QYB1	35	AGSDGESIGNCPFSQRLFMIL
Q60714	170	VNLRREPLAFCLGTSAAKALI
Q62234	1601	NEKPLTSDDHCSLKFEAGKTA
Q9D404	86	VVGDEYKNIPCSVAAYVPRGP
Q9Y6I9	68	GETGRLFTESCSISPKLRSIA
P47962	62	IVRVTNRDIICQIAYARIEGD
Q7TNG8	63	QHGHDESMHRCQPPDAVVWPQ
P54577	501	LQADFKISEECIAQWKQTNFM
Q96AG4	59	NKLTTLPSDFCGLTHLVKLDL
Q9CPP6	17	KTTGLVGLAVCDTPHERLTIL
Q9CPV4	182	RALLGYADNQCKLELQGIQGA
Q6P3A8	175	YRYRSGDLFNCGSLTIRAPWG
Q9JLV5	636	ELVRALQSLACGKPTQRVLTK
P52825	639	SGRNAREFLHCVQKCLEDMFD
Q921G7	247	HAKVTVFAEGCHGHLAKQLYK
Q9BW61	25	NFSRFHADSVCKASNRRPSVY
Q01433	230	QRVTISGEEKCGVPFTDLLDA
P05125	145	DRIGAQSGLGCNSFRYRRXXX
Q9DCB8	56	GEGQIRLTDSCVQRLLEITEG
Q91VR2	103	IIGVSSDRGLCGAIHSSVAKQ
Q61024	207	ASVEMVKYHHCTDEPLHAIYD
P35486	181	VPLGAGIALACKYNGKDEVCL

Q16658	397	VFRGEHGFIGCRKVTGTLDAN
P97443	90	KDAWLNHKNECAAIKKYGKVP
Q9JI57	230	ISLLPKGSRDCGLHGQASKVA
Q9DCS3	169	QSAATLGVNPCTAYRMLVDFE
Q8BG93	75	EEAGLHLKNVCFASVVNSFVE
Q7KZF4	440	ETVPAFSERTCATVTIGGINI
Q91YT0	425	ISKQIEGHTICALGDGAAWPV
Q9CZ13	410	VSHLDGTTPVCEDIGRSLLTY
P34932	38	NEYSDRCTPACISFGPKNRSI
P23492	142	IRDHINLPGFCGQNPLRGPND
Q9D0I9	86	INSRLQEVFGCAIRAAYPDLE
Q8VCW8	467	VPGELYIRGYCVMQGYWGEPQ
Q9D892	33	VIQILGDNFPCTLEAQKIDLP
P52503	79	VNEVEHRIIACDGGGGALGHP
Q9UNM6	182	YYKDALRFLGCVDIKDLPVSE
Q60676	77	NRSLAYLRTECYGYALGDATR
P68104	411	IVDMVPGKPMCVESFSDYPPL
O00231	289	YAGRQTEALKCVAQASKNRSL
P40926	285	CSFVKSQETECTYFSTPLLLG
Q91V92	20	TGKELLYKYICTTSAIQNRFK
P17563	268	DPSATQGFVGCALSSNIQRFY
Q8K0C8	92	MEGKPEAKDECXXXXXXXXXX
A2AVZ9	251	HETKELRSKECLPPKEENSGP
Q99LC5	68	TKCDKVVQDLCKVAGVAKVLV
Q07417	151	TPFTNGDKIGCFALSEPGNGS
O09131	191	QRLEALELKECLAHTPKLKLW
P17751	177	ALAEGLGVIACIGEKLDEREA
Q9JHU4	631	FKVQYPQSQACKMSHVRDLPP
P05201	253	YFVSEGFELFCAQSFSKNFGL
Q99KC8	279	DIPEVEASKACGEFVFLMDRS
Q9Z0N1	105	SCPRPECYRSCGSSTPDEFPT
P54819	92	LIEKNLETPLCKNGFLLDGFP
Q6PDY2	225	IRPKEASGSACDLPREVWLLE
O95573	561	FEDENGQRWLCTGDIGEFEPD
Q8CC88	1064	QTGNGMQKVLCPAETNHVDIK
Q8BTM8	2378	VHSPSGALEECYVTEIDQDKY
Q91WS0	72	MEDLGDKAVYCRCWRSKKFPF
Q14103	126	YFSKFGEVVDCTLKLDPITGR
O43765	153	GAVQDCERAICIDPAYSKAYG
O55143	471	GLSKIERANACNSVIKQLMKK
O08739	322	KVDTHIHAAACMNQKHLLRFI
P28482	65	KISPFEHQTYCQRTLREIKIL
Q9JHR7	904	LDKPKKLSAECAKYWGEIISQ
P52272	114	LMDAEGKSRGCAVVEFKMEES
P21980	277	HGCQRVKYGQCWVFAAVACTV
P70195	74	TEGMVVADKNCSKIHFISPNI
Q8BVZ1	51	VVALPLVKATCTAVSSAYNSA
Q5VYK3	1257	GQRTIAALLPCLLDKGMMSTV
Q3TBW2	180	SVPFLPLLGGCVDDTILSRQG
Q61792	53	NYKGYEKKPYCNAHYPKQSFT
P78527	795	MQPYYKDILPCLDGYLKTSAL
P26039	1199	AKAVTQALNRCVSCLPGQRDV

Q8CHP8	217	ENGRFIAGTGCLVRAVEMAAQ
O15382	345	VFGSGTACQVCPVHRILYKDR
Q9CZ13	69	VASEQSSHATCTVGVWIDAGS
Q9JHW2	153	TPYCKVGLGICYDMRFAELAQ
Q9JLV1	185	ERSQSPAASDCSSSSSSASLP
Q92879	61	RSQNPPQSKGCCFVTFYTRKA
O95433	207	ARPVGVKIPTCKITLKETFLT
P26039	1661	MRDKAPGQLECETAIAALNSC
P16332	740	AVQVLDDIEKCLAEKQQSVXX
Q9DB20	141	IMSVHRGEVPCTVTTASPLDD
P05089	168	DVPGFSWVTPCISAKDIVYIG
Q04750	632	NRANRAVAILCNHQRAPPKTF
Q8R3F5	118	PQEDLDRTVHCQPAVFVASLA
P55263	160	SLIANLAAANCYKKEKHLDLE
P48962	57	AEKQYKGIIDCVVRIPKEQGF
Q9D1P0	53	NKPVYHQLSDCGDHVVIINTR
P14152	154	APSIPKENFSCLTRLDHNRAK
Q921H8	177	RLLESEKARDCLTPMGMTSEN
P58281	786	MYWKNRTQEQCVHNETKNELE
Q15274	96	RVAEVRGPAHCLLLGERVALN
Q9CR00	59	GIGMNEPLVDCEGYPRADVDL
Q06587	398	FWKVSRPLELCYAPTKDPKXX
Q9CW46	222	QLTPALLHSRCLCVDHLPPGF
Q8JZQ2	625	YTKEQLLDRMCMTLGGRVSEE
Q8N0X7	504	LVDGVCTVANCVGKELAPHVK
P46459	264	KGILLYGPPGCGKTLLARQIG
Q9Z2Z6	165	CAKKLYQEFGCRGFYKGTVLT
Q9D1Q6	92	KNQVVFARVDCDQHSDIAQRY
P49327	1118	EQQVPILEKFCFTPHTEEGCL
Q8TAQ2	145	NCLSRPNIFLCPEIEPKLLGK
Q9Y3C8	116	TAKMYRGGKICLTDHFKPLWA
P70670	354	KGSSAVTNELCSPPGSSNVAG
P62984	115	PRAVNCRKKKCGHTNNLRPKK
O94979	60	LSDPSLDMKSCATFSSSHRYH
P05141	160	AEREFRGLGDCLVKIYKSDGI
P14873	2460	VVMQDESFPACKIELXXXXXX
Q8BTM8	483	TVGQACNPAACRAIGRGLQPK
Q9UL62	558	DEPNNCKGIRCEKQNNAFSTL
P50544	238	SIRSSAIPSPCGKYYTLNGSK
P16675	438	GEQVAGFVKECSHITFLTIKG
P41216	109	RGIQVSNNGPCLGSRKPNQPY
Q9UBF2	325	AMKHPSAVTACNLDLENLITD
P08397	114	LPPGFTIGAICKRENPHDAVV
Q91YR7	837	KTKSVDALKKCEHDPHVLLAV
O94927	349	RQVLILGLRRCCLWTELKALH
P30044	100	FGVPGAFTPGCSKTHLPGFVE
O70250	55	IKDAKIEFDICYTSVLKRAIR
P24452	77	QQSSRDEQGACAVLAVHLNTL
Q9D6S7	154	ILVNMASFPECTAAAIKAIRE
P98170	12	TFNSFEGSKTCVPADINKEEE
P78527	478	LAAKGPVLRNCISTVVHQGLI
Q9NPH0	183	TRCLLAGLFQCQKEGPIIIHT

O75694	974	AFQERLNSYKCITDTLQELVN
Q9BSK1	152	KSQLNDLQKICAGGKPHECSV
Q01853	174	KVVETDPSPYCIVAPDTVIHC
Q6PB66	847	LPAALEASIACHKKYKVLPRI
Q8N0X7	499	KVSQFLVDGVCTVANCVGKEL
Q6R5N8	527	KLQSLILSHNCLKILEPNSFS
P97390	172	LTALLLSLKKCPMIRYQLSSE
Q9P1F3	39	KFGVLFRDDKCANLFEALVGT
O08528	628	KWTKGFKASGCEGEDVVTLLK
Q91VD9	554	MLFLLGADGGCITRQDLPKDC
Q8BKC5	733	AAESMPLLLECARVRGPEYLT
Q6PIE5	660	SQVNPREAKACVVHGSDLKDM
P34932	245	KFDEVLVNHFCEEFGKKYKLD
Q62159	16	KKLVIVGDGACGKTCLLIVFS
P35486	273	REATKFAAAYCRSGKGPILME
Q61233	31	VDTDGNGYISCNELNDLFKAA
Q9R0H0	531	VDLVRASEAHCHYVTVKVFAD
P62983	149	HYCGKCCLTYCFNKPEDKXXX
P32020	495	VLPNSDKKADCTITMADSDLL
Q8CI94	758	SGFFSPKDPDCFKDVVNMLMY
Q60770	558	AYEVSQAHKSCEVIIGSTHIL
Q8R1S0	116	LSSFGAWDHICNMRCKAFRRM
Q04750	506	EEGETADTVGCCSLRVEHINL
Q08211	12	GDVKNFLYAWCGKRKMTPSYE
Q99JY9	408	KDYEEIGPSICRHNPVFGVMS
P62888	92	GTACGKYYRVCTLAIIDPGDS
P16615	560	EWGSGSDTLRCLALATHDNPL
P61081	65	DDLLNFKLVICPDEGFYKSGK
O70325	134	NVKFDMYSKICVNGDDAHPLW
Q7TPV4	1043	KTLSARELRVCFEDPEWEQLI
P51660	175	SAAKLGILGLCNTLAIEGRKN
Q7TNS2	58	GVGLGMAYSNCQHDFQAPYLL
Q9CQQ7	239	VQQEKETIAKCIEDLKLLAKK
P13489	38	QQCQVVRLDDCGLTEARCKDI
P52272	694	IKMENGKSKGCGVVKFESPEV
Q9JLV1	378	EVKVSSAPIPCPSPSPAPSAV
P97443	417	GHIEVGHGMICKAYAILLVTH
Q60597	507	FHKDVVVDLVCYRRNGHNEMD
O14744	22	SRVSSGRDLNCVPEIADTLGA
P01009	256	KRLGMFNIQHCKKLSSWVLLM
Q61553	80	LAADKDGNVTCEREVPDGDCR
O55143	498	SRDRKSMSVYCTPNKPSRTSM
Q99JB8	231	MEDMEQAFESCQAAERQRLLF
Q9Y237	45	GNAVKVRHILCEKHGKIMEAM
P12979	61	EEKGLGTPEHCPGQCLPWACK
Q8K4Z3	277	LNLPSYPDTECVYRLQXXXXX
P98192	137	KIFKQIFSKVCVNEEGIQKLQ
Q9EQQ9	863	DPSVAKSMMACLLSSLKANGS
Q15366	109	TLRLVVPASQCGSLIGKGGCK
P38646	608	EFKDQLPADECNKLKEEISKM
Q8BVI4	82	LGDQKVDAILCVAGGWAGGNA
Q9NR33	85	VETIAKDAYCCAQQGKRKTLQ

Q9CZ42	257	LISNGQQVLVCNQEGSSRRCG
P09382	61	NAHGDANTIVCNSKDGGAWGT
P84095	157	KQIHAVRYLECSALQQDGVKE
P29474	802	QYQPGDHIGVCPPNRPGLVEA
Q05816	120	KLKDGKMIVECVMNNATCTRV
Q791T5	222	AHPLHVISMRCMVQFVGREAK
P50462	120	FSAKFGESEKCPRCGKSVYAA
P40926	212	GKTIIPLISQCTPKVDFPQDQ
P50544	478	ILRLFVALQGCMDKGKELTGL
Q9DBB8	95	LCLAAGKAVLCEKPMGVNAAE
Q9CQJ8	31	KRALRHLESWCIHRDKYRYFA
P60900	154	DEEQGPQVYKCDPAGYYCGFK
P60766	105	EKWVPEITHHCPKTPFLLVGT
Q9UNE7	199	DDSHVRAQQACIEAKHDKYMA
Q91YT0	187	YEAGLIGKNACGSDYDFDVFV
P70310	153	ARRLLTPRRTCRLLSLVWVAS
Q9D273	179	GGKSSSALHFCRAVCRRAERR
O55091	38	CVIDENAKIFCIRVTDFMDDP
P55036	37	QAQQDAVNIVCHSKTRSNPEN
Q9Z2I8	256	FGETPEGQVVCFDAKINFDDN
Q8BY71	24	AVEKKLAEYKCNTNTAIELKL
Q9D0I9	638	LKVNMWRMLLCEAVAAVMAKG
Q9BXP5	640	YPNEDEMPNRCGIIHVRGPMP
P08249	275	AMNGKEGVVECSFVQSKETEC
O70370	329	IRMARNNKNHCGIASYCSYPE
Q80Y14	63	FLKGTPEQPQCGFSNAVVQIL
Q9CY45	79	DAAGEGGRIACVSAPSVYQKL
P26638	300	LPIKYAGLSTCFRQEVGSHGR
P11142	603	EHQQKELEKVCNPIITKLYQS
Q9CQ54	40	RLVYMGLLGYCTGLMDNMLRM
Q8QZT1	116	LGAGLPISTPCTTVNKVCASG
Q8BRK8	174	MSDGEFLRTSCGSPNYAAPEV
Q80X90	2057	IQTEDLEDGTCKVSYFPTVPG
Q91WD7	143	MYLTMLDLFKCIDEIKEEKEC
P68040	207	TVTVSPDGSLCASGGKDGQAM
P20810	661	IDALSGDLDSCPSTTETSQNT
Q9QZ06	229	PPPAVAPQPRCNEEDLKAIQD
Q9CWJ9	101	LDFNLVRVVVCNLYPFVKTVA
Q6PIE5	702	PQQKLIIVEGCQRQGAIVAVT
P47915	117	RSYMAKGQRLCQPKPKVQTKA
Q9D8B4	95	DDPLNYFIGGCAGGLTLGART
Q9D1C3	58	FHPALLQFLVCPLSKKPLRYE
Q71RI9	417	KLTAIPVSAFCDSKSKPHFEK
P62869	60	LLDDGKTLGECGFTSQTARPQ
P04075	73	LLTADDRVNPCIGGVILFHET
Q16623	145	NATQSDYRERCKGRIQRQLEI
P60710	272	FQPSFLGMESCGIHETTFNSI
P62826	120	RVCENIPIVLCGNKVDIKDRK
Q9JIX8	1051	AFWIDKIKSHCFVTYSTVEEA
P45376	45	AIDLGYRHIDCAQVYQNEKEV
Q8BUN5	121	FAFNMKKDEVCVNPYHYQRVE
Q99JX3	434	PTTVEDRVSDCTPAVEKPVSD

P31939	434	AVKYTQSNSVCYAKNGQVIGI
Q68FD5	1102	LDRAYEFAERCNEPAVWSQLA
Q810L3	341	WMERSSLCPTCRCPVERICKN
Q80X90	2289	PVIAPSDDARCLTVLSLQESG
O75369	1326	SPFKVAVTEGCQPSRVQAQGP
P17987	147	IVNTDELGRDCLINAAKTSMS
Q921H8	381	GAIALGHPLGCTGARQVVTLL
Q02257	457	DKDDITEPAVCALRHLTSRHP
Q9WUM4	23	VFGQAVKNDQCYDDIRVSRVT
Q8VEM8	86	TAVVPLDLVKCRMQVDPQKYK
Q8BGH2	403	THFFLNAGNLCNLNYGEGPKA
Q8VBT1	275	EQQSERNMKLCQENTELAEKL
A2A432	694	KSMLSKLKHECGAAFTSKLEG
Q8QZT1	410	KPGEFGLASICNGGGGASALL
P50579	448	LGIVDPYPPLCDIKGSYTAQF
Q9D172	151	LSTFAVDGKDCKVNKEVERVL
Q6PEB6	134	YTRHTLDGAACLLNSNKYFPS
P30999	429	HPKKEVHLGACGALKNISFGR
P97447	65	HYKNRYWHDNCFRCAKCLHPL
Q96AG4	131	DPVLAKVAGDCLDEKQCKQCA
P00750	118	CQCPEGFAGKCCEIDTRATCY
P21980	269	DILRRWKNHGCQRVKYGQCWV
Q8BP47	125	KNDPSLPEPACVKISALEGYR
Q12879	399	VWPRYKSFSDCEPDDNHLSIV
Q9CPV4	41	VLRHEEFEEGCKAACNGPYDG
P14152	137	IVVGNPANTNCLTASKSAPSI
Q99KI0	740	KDFAPGKPLKCVIKHPNGTQE
Q8QZT1	139	AIMMASQSLMCGHQDVMVAGG
Q9UQM7	6	XXXXXMATITCTRFTEEYQLF
P11499	521	VYMTEPIDEYCVQQLKEFDGK
Q08752	282	ALSCVLNIGACKLKMSNWQGA
P46777	76	YARIEGDMIVCAAYAHELPKY
P31948	62	GDYQKAYEDGCKTVDLKPDWG
Q9D1H8	63	ADVRHDGSEPCVDVLFGDGYR
Q8BMS1	349	LMGLYNGQVLCKKNKFGAPQK
Q9ET26	161	NFDQEGLVEHCKLTHSTDTKS
P32067	18	EKMTALEAKICHQIEYYFGDF
Q8WX93	964	TANQEYKVSSCEQRLISEIEY
Q99L45	224	KTSFVNFTDICKLLHRQPKHL
Q9D1L9	66	TSDPTDIPVVCLESDNGNIMI
P55060	939	AQSLHKLSTACPGRVPSMVST
P38060	307	QKLLEAGDFICQALNRKTSSK
Q8BTM8	1453	HDVTDASKVKCSGPGLSPGMV
Q8BIJ6	364	FSGVDLEGGTCSHPLTPDKVS
P05062	269	RTVPAAVPGICFLSGGMSEED
P62983	145	HFDRHYCGKCCLTYCFNKPED
A3KMP2	363	QEASKSPGENCQHQLAKDVGL
P47934	287	NSIQKSIFTVCLDKQVPRVSD
P62826	112	PNWHRDLVRVCENIPIVLCGN
Q9R1P4	92	RLLCNFMRQECLDSRFVFDRP
Q3UHX9	140	FRGVGKKGQACVQLARILQYL
P35282	27	SFKVVLLGEGCVGKTSLVLRY

P62761	187	SDPSIVLLLQCDIQKXXXXXX
P62908	134	RFIMESGAKGCEVVVSGKLRG
P51410	135	RVRMRTGVACCVSQAQKDELI
Q61598	414	YDATTHFETTCDDIKDIYKRM
Q99NB1	235	KKIVDEAVKSCPTVQHVLVAH
Q76KJ5	107	SSSEAGGRLTCAPAPSGSLRI
P47738	68	TVNPSTGEVICQVAEGNKEDV
Q9WVA4	63	QKWLKDGTVLCKLINSLYPEG
Q3UH68	929	QLEAEAGAPHCGTNPQPAQDP
Q60973	166	HPAKPDPSGECNPDLRLRGHQ
P00533	311	VTDHGSCVRACGADSYEMEED
P31943	34	ADEVQRFFSDCKIQNGAQGIR
P61971	80	AQDHQPTPDSCIISMVVGQLK
Q9DCC4	49	LASAPTDNNLCHFRALGCQTT
Q12931	501	RAGTRNIYYLCAPNRHLAEHS
P10242	130	KHLKGRIGKQCRERWHNHLNP
Q9CQ75	58	HPNLPILIRECSEVQPKLWAR
Q9DCN1	177	ESSQQPEVRLCQLNYPDVKGY
Q9DBM2	17	LPHSLAMIRLCNPPVNAISPT
P61978	184	TQTTIKLFQECCPHSTDRVVL
Q8K4Z3	152	KPLFTGLVTQCQKMDIPFLGE
Q8C0M9	219	KMVGRVGDSPCIGAGGYADNN
P08113	138	GNEELTVKIKCDKEKNLLHVT
P68104	234	SGTTLLEALDCILPPTRPTDK
Q3TL44	644	HNRAVLAQLGCPIKNLDALEN
Q9D7P6	70	VGTGLVGAPACGDVMKLQIQV
Q99J39	359	GRNELFTDSECQEISAVTGNP
P09671	164	KEQGRLQIAACSNQDPLQGTT
Q8C1E7	47	LEELTKLQANCTNSITRQKKR
P80313	310	TQYFADRDMFCAGRVPEEDLK
P60766	6	XXXXXMQTIKCVVVGDGAVGK
Q8VEK3	583	AGFQRKAVVVCPKDEDYKQRT
P78527	3187	NIWDDIITNRCFFLSKIEEKL
P17426	941	IIQTKALQVGCLLRLEPNAQA
Q15149	3299	VPLDVACARGCLDEETSRALS
Q2TPA8	11	MLPNTGKLAGCTVFITGASRG
Q9D7I5	226	IVGDVGGAQQCGMRALQVRTG
Q8BWY3	127	FKPINTSLYLCDNKFHTEALT
Q8BZF8	106	NGILSTPAVSCIIRKIKAAGG
Q9DAW6	224	HKSLRSLNNFCSQIGDDRPIS
Q9Z0N1	269	IRSFDVNKPGCEVDDLKGGVA
Q9D0I9	502	KAAQTSVAYGCIKYADLSHNR
Q8VBT1	36	NGPGKQDGERCSTSGQAPEQE
Q8BIG7	88	EQPQGDSMMTCEQAQLLANLA
P14866	452	NFMFGQKLNVCVSKQPAIMPG
P62280	131	QIGDIVTVGECRPLSKTVRFN
Q9CQN1	135	ALEKLRHKLVCEGQVLPEMEI
Q9DCS3	263	DLPLPRLALNCVGGKSSTELL
Q9DB29	47	SLLADRLVRKCDVLNRGFSGY
Q64514	209	VWHDGETWRACVDSNENGDLS
P54886	612	RDSKCEYPAACNALETLLIHR
Q8BGH2	237	KWEGVWRELGCLSRTASFAVR

P49368	173	KAISRWSSLACNIALDAVKMV
Q924D0	236	KAWGAHVTAVCSKDASELVRK
P49411	290	ERGILKKGDECELLGHSKNIR
Q9DCM2	176	NKLIENTDAACKYGAFGLPTT
P07900	598	VSNRLVTSPCCIVTSTYGWTA
Q9ER35	130	KSKTRQNTVGCGAEGAEPQGV
Q923E4	387	GDIFNQVVPRCPRCPADEPLA
Q99J39	205	NLERVTWHSPCEVLQKISECE
Q99N94	167	GEATVKFLRSCHLEVGMKNNV
P17812	362	VRYHEAWQKLCSAHGVLVPGG
P14618	152	ITLDNAYMEKCDENILWLDYK
P29474	1050	TPMTLVFGCRCSQLDHLYRDE
Q8CC88	161	RAGTAFYIDQCAVRAATEGRT
P16125	36	VVGVGQVGMACAISILGKSLA
Q9CQ60	236	ALVQPRTGALCWFLDEAAARL
Q86VP6	1134	FLMLVRLSTLCPSAVLQRLDR
Q8BKZ9	307	TVPHAYATADCDLGAVLKVRR
Q14181	198	AGNISLKVLGCPEALTGSYKS
P22460	346	TFELLVRFFACPSKAGFSRNI
P14550	134	PFPKNADGTICYDSTHYKETW
Q9Z1Z2	305	VVGKTYGLWKCVLPEEDSGEL
P04075	339	VKRALANSLACQGKYTPSGQA
Q99MK8	340	HVRISDLGLACDFSKKRPHAS
O70325	175	FTKFLIDKNGCVVKRYGPMEE
Q9D1G3	347	HFDRGINDWLCKYVYDHIGGD
P52825	84	DSQFRKTEVLCKDFENGIGKE
Q15149	3295	KSHRVPLDVACARGCLDEETS
P17563	371	VQVLEDQELTCQPEPLVVKGK
O00170	240	ITPLLLNYCQCKLVVEEYYEV
P28651	201	QYKGKSKTIPCFNPNTLLPDP
Q8BTM8	2601	LVGVHGPRTPCEEILVKHMGS
P09528	131	LATDKNDPHLCDFIETYYLSE
Q791T5	206	KETSYEMMMQCVSRMLAHPLH
Q12879	87	TDPKSLITHVCDLMSGARIHG
P21283	376	YYPYVYYKIDCNLLEFKXXXX
Q15233	145	RGKQLRVRFACHSASLTVRNL
Q62418	127	ARAEEDVEPECIMEKVAKASG
P37804	38	ERLVEWIVVQCGPDVGRPDRG
Q64702	4	XXXXXXXMAACIGERIEDFKV
Q7TQ48	648	QERGYPFNDVCQWFIDRADLI
P09211	48	TWQEGSLKASCLYGQLPKFQD
Q64514	150	PIHRVALAEACRKQEEFDIAN
Q91Z53	288	PSHPLLTLKNCVILPHIGSAT
Q9EQQ9	596	RANSSVVSVNCKGKDSEKIEE
Q8BFP9	71	QFLDFGSVNACEKTSFMFLRQ
Q9Z0S1	59	KADRLVQMSICSSLARKFPKL
Q9DBG3	857	ENELQFQIKECHLNADTVSSK
Q8K2B3	89	GLSEAGFNTACLTKLFPTRSH
P05063	135	TQGLDGLLERCAQYKKDGADF
Q64337	145	VVGTRYKCSVCPDYDLCSVCE
Q4G0N4	393	NRVFSSSRQRCFSSKVCVRSR
Q60714	406	IANMDGKVGSCGFNSRILTHV

P52480	165	NILWLDYKNICKVVEVGSKIY
O08528	606	LPLGFTFSFPCQQNSLDQSIL
Q09161	73	NYKSKILRLLCTVARLLPEKL
Q9WU78	40	PSGGEEQAQYCRAAEELSKLR
P18760	139	TGIKHELQANCYEEVKDRCTL
P14780	99	ATLKAMRTPRCGVPDLGRFQT
Q8NC51	11	MPGHLQEGFGCVVTNRFDQLF
Q8CHT0	279	GDTVTSSEHLCGINFTGSVPT
O00170	238	QQITPLLLNYCQCKLVVEEYY
Q8QZR5	29	KVLTLDTMNPCVRRVEYAVRG
Q15149	1098	RLMAEREYGSCSHHYQQLLQS
Q8R349	133	MSQSSIKSSICLLRGKIYDAL
O70251	161	DETDMTKLEECVRSIQADGLV
Q8CEI1	62	AIQVTDISGGCGAMYEIKIES
P35579	91	SKVEDMAELTCLNEASVLHNL
P30042	177	HQAGKPIGLCCIAPVLAAKVL
Q9DBF1	478	FRWLGPKGSDCGIVNVNIPTS
O88587	200	YLPDTLLLEECGLLRKGTVLL
O55143	364	GTLTTNQMSVCRMFILDKVEG
Q99KB8	220	GRLPPDTKVYCGHEYTVNNLK
Q9Y2Z0	88	NSTAMLRKGICEYHEKNYAAA
O43765	148	LGNYAGAVQDCERAICIDPAY
A8C756	1910	RTLAVLRLLACLEGKEGLRAE
Q9D6N1	232	QQLARFRSLLCTAEGESAAFL
P61161	11	MDSQGRKVVVCDNGTGFVKCG
P21980	27	NGRDHHTADLCREKLVVRRGQ
P61922	440	ISRVRGRGTFCSFDTPDEAIR
P05089	303	VNTAVAITLACFGLAREGNHK
P04350	12	REIVHLQAGQCGNQIGAKFWE
Q9DCL9	281	LMGSTSDLGHCEKIKKACGNF
P53702	70	HQDRAYDYVECPVTGARAKDK
Q8BIF0	212	VKGENLEAVVCEEPQVTYSKQ
P30042	176	FHQAGKPIGLCCIAPVLAAKV
O08528	517	APVKMLPTYVCATPDGTEKGD
Q8JZN5	275	DKLGIRGSNTCEVHFENTRVP
Q02566	37	QTRPFDIRTECFVPDDKEEYV
Q9QYG0	78	YHDVGLNYKSCFQPLFRFGDM
Q9R1P4	85	AGLTADARLLCNFMRQECLDS
Q8BGK2	206	LKVLPLAEEYCRKTIRHMAEY
P49327	642	QRCPPGVVPACHNSKDTVTIS
P29474	661	GLGSRAYPHFCAFARAVDTRL
Q9QYC0	525	KTAGPQSQVLCGVMMDRSLVQ
P61247	139	TTDGYLLRLFCVGFTKKRNNQ
Q8BVI4	101	NAKSKSLFKNCDMMWKQSMWT
P07356	262	LENAFLNLVQCIQNKPLYFAD
Q64520	98	VRAVQAMNRICVLDVDLQGVR
Q8BH86	54	RSLAPAFESFCQGNRGPLPLL
Q60930	104	GTEIAIEDQICQGLKLTFDTT
P46777	100	VGLTNYAAAYCTGLLLARRLL
Q8CIB5	426	TPAHQLNLRGCEVTPDVNISG
P41091	236	AQLKYNIEVVCEYIVKKIPVP
Q9CZU6	359	VLRKTDPRYSCQREFALKHLP

Q9CPR5	8	XXXMAGTARGCGTSLDLLRSL
Q78PY7	152	NNPEQNRLSECEEQAKASKKG
P17987	397	EMERSLHDALCVVKRVLESKS
P09411	367	DEVVKATSRGCITIIGGGDTA
Q921G7	386	LTFPGGLLIGCSPGFMNVPKI
Q9CQ92	41	SKSTQFEYAWCLVRSKYNEDI
Q01320	404	SEKFIKAAIGCGIVESILNWV
P26639	107	SWKTTPYQIACGISQGLADNT
Q61553	341	NASCYFDIEWCDRRITLRASN
P14854	54	MTAKGGDISVCEWYQRVYQSL
P13639	591	ETVSEESNVLCLSKSPNKHNR
Q80UJ7	858	SLKAKFGTEKCEHEEEKEGLE
P62736	259	VITIGNERFRCPETLFQPSFI
O08749	484	GASCEDIARVCHAHPTLSEAF
Q9ET80	29	EEGKAHGHGICTGPKGQGEYS
Q8VBZ3	645	STVPKATSGACTASQPQEAPP
P15880	229	IDDCYTSARGCTATLGNFAKA
Q91VD9	75	YHERLSVAGNCRMCLVEIEKA
Q9JLJ2	45	ATGRVIATFACSGEKEVNLAV
Q9D0M3	219	DYVFSLLTGYCEPPTGVSLRE
P23368	441	EEAYTLTEGRCLFASGSPFGP
A3KMP2	425	NQLLIHAAMTCTSSVHKNVAR
Q9D6M3	271	NEDTYSGFLDCARKIWRHEGP
Q3UMB9	936	IRMIRSGGLHCSSNAIRFVPD
Q9Z0N1	434	LGKIVLTNPVCTEVGEKIALS
Q921G7	100	AAEQGKDIRVCLVEKAAQIGA
Q99MK8	619	VEETQIKERKCLLLKIRGGKQ
Q9UI30	33	LRLQATEVRICPVEFNPNFVA
Q9R0H0	392	TWTANAGIEECRMACGGHGYS
Q99K43	184	FVNIKKQIILCMEELEHSPDT
Q9CPY7	335	PINIIGLAPLCENMPSGKANK
P26443	172	KALASLMTYKCAVVDVPFGGA
O15355	13	AYLSQPNTVKCSGDGVGAPRL
P49721	91	ANFTRRNLADCLRSRTPYHVN
Q6P2B1	125	LALQMPSWKGCVQTLVEKYSN
Q29RF7	1084	KLYTVCDVALCVINSKSALCN
P97447	255	WHDYCFHCKKCSVNLANKRFV
Q9DCM0	189	HEKIFTLPGNCLIYPAHDYHG
Q8BGH2	65	LGRTKDDIIICEIGEVFKAKN
P13010	346	EGKCFSVLGFCKSSQVQRRFF
Q8BMF4	613	VASVMSVTLSCDHRVVDGAVG
P22314	234	VTKDNPGVVTCLDEARHGFES
Q8VDK1	63	TPNKQENFKTCAELVQEAARL
O00231	202	SARTTANAIYCPPKLQATLDM
Q9D0T1	102	ACGVSRPVIACSVTIKEGSQL
Q07417	289	ALGIAQASLDCAVKYAENRNA
Q9CPU0	139	GIAVPDVYSACKRFEELGVKF
Q2TPA8	71	IEAAGGTALPCVVDVRDEQQI
O15382	342	VREVFGSGTACQVCPVHRILY
Q99700	301	EGVFKTYSPKCDLVLDAAHEK
P30681	23	MSSYAFFVQTCREEHKKKHPD
P10605	93	TFDAREQWSNCPTIGQIRDQG

Q9D7I5	53	LKQSPLKVRFCTNESQKSLRE
P04350	303	MFDAKNMMAACDPRHGRYLTV
Q16543	336	PTDAKYHMQRCIDSGLWVPNS
Q08J23	93	YKSHAKEILHCLKNKYFKELE
P50544	434	SEAAWKVADECIQIMGGMGFM
P20337	184	QAFERLVDAICDKMSDSLDTD
Q6ZWY3	77	TGGKARLTEGCSFRRKQHXXX
Q9DCT8	126	VTTFTGEPNMCPRCNKRVYFA
P06213	1272	PERVTDLMRMCWQFNPKMRPT
P00533	329	EEDGVRKCKKCEGPCRKVCNG
Q99KI0	410	AKQALAHGLKCKSQFTITPGS
O55234	161	GMGLSMGTMICGWDKRGPGLY
Q9R0Q7	58	KHLNEIDLFHCIDPNDSKHKR
Q8BWT1	287	ARVVGYFVSGCDPTIMGIGPV
Q80X90	1280	ITNPSGASTECFVKDNADGTY
P48643	181	TTLGSKVVNSCHRQMAEIAVN
Q71RI9	432	KPHFEKLVRFCFIKKDSTLDA
P62821	26	LIGDSGVGKSCLLLRFADDTY
P36578	96	RSGQGAFGNMCRGGRMFAPTK
P15626	87	LRYLARKHNLCGETEEERIRV
P41250	466	IEIVGCADRSCYDLSCHARAT
Q9EQK5	59	RMVTVPPRHYCIVANPVSRDA
P61202	179	QKILRQLHQSCQTDDGEDDLK
P08752	140	RLWADHGVQACFGRSREYQLN
Q8BTM8	1018	IVSPSGAAVPCKVEPGLGADN
Q9EPL8	757	LQCKGRGIDQCIPLFVEAALE
Q5XJY5	479	QVSFISKKNYCNIQVTKVTQV
P63167	56	FDKKYNPTWHCIVGRNFGSYV
Q8QZZ7	167	RIGTLLDAIICRMSTKDVLXX
Q8VEK3	449	EKPYFPIPEDCTFIQNVPLED
P97807	330	ELSGAMNTAACSLMKIANDIR
P29474	1048	QPTPMTLVFGCRCSQLDHLYR
O95219	172	LLRIASHPILCRDKIFYLFLT
Q8R164	206	LYGYDYLAKTCEDWVDGISQF
P62070	183	RVIRKFQEQECPPSPEPTRKE
P52825	643	AREFLHCVQKCLEDMFDALEG
Q9WVJ2	357	KGMKDRLELWCTDVKSMEMLV
P99029	200	NVEPDGTGLTCSLAPNILSQL
Q05519	79	DSPLPVSSRVCFVKFHDPDSA
Q61553	260	KDELFALEQSCAQVVLQAANE
P63330	196	LQEVPHEGPMCDLLWSDPDDR
P18031	32	DIRHEASDFPCRVAKLPKNKN
P21333	444	LEARGDSTYRCSYQPTMEGVH
Q9CZY3	71	ENRIYSLKIECGPKYPEAPPS
Q924X2	155	TSHATKIWAICVRLLSSRRPM
P21817	810	KFLPPPGYAPCHEAVLPRERL
P61759	8	XXXMAAAKDGCGLETAAGNGR
P70670	1763	DSHISPVSDACSTGTTTPQAS
Q64277	123	GENTRRLVALCDVLYGKVGDF
P70388	206	RQTQGQKVKECQTELKYLKQN
Q91YT0	125	VNADEGEPGTCKDREIMRHDP
O55143	635	GDNKGTAVAICRRIGIFGQDE

Q924X2	526	QRLPWDIPEQCREAIENSYQV
P56380	124	YRWLGLEEACCLAQFKEMKAT
P43243	806	IPKTGFYCKLCSLFYTNEEVA
Q3UKJ7	312	FERAHSKGVTCLSFSKDSSQI
Q60714	80	RHRRAGDTIPCIFQAVARRQP
Q8QZT1	193	DVYNKIHMGNCAENTAKKMNI
Q8C025	386	HLHLNIFKTSCQQAPEQVYKH
Q71FD7	35	AVSEEVGQAACEARRARPWEM
Q8VC70	190	GFARMESTEKCEAIITHFNGK
Q6A0A9	917	AFRVTAASGHCGAFSGSDSSR
Q60631	198	NSDPNWWKGACHGQTGMFPRN
Q7TPV4	676	QVVRSVFGHICPHLTPRCLQL
Q00610	736	DVHFKYIQAACKTGQIKEVER
Q99NB1	86	WDTPYHTVWDCDFRTGKIGWF
Q5SW19	1198	KTKESSEYLKCLTQQAVALQR
Q80XN0	209	RMANPARSPYCITKFGIEAFS
Q8BGH2	421	PKAHIRKLAECIRWSYGAGVV
Q9JLV1	154	MTEAAQTDKQCGQMPATATTA
P27348	25	QAERYDDMATCMKAVTEQGAE
Q9DB26	49	IVAEMDVPLHCRTEFSTQEDE
P54729	399	AQEARLGLRACDGNVDHAATH
P97429	198	TDEVKFLSILCSRNRNHLLHV
Q9DCW4	131	FLGKQAIDDDCNQTGQMTAGL
P46782	172	AFRNIKTIAECLADELINAAK
O55222	239	TRKSRDFNEECPRLRIFSHPN
Q9DCA2	109	ATNASLARASCGTEGFRNAKK
P62245	30	RGKRQVLIRPCSKVIVRFLTV
P29474	94	LSAQAQQDGPCTPRRCLGSLV
Q9Z2I9	430	IADSGLKILACDDLDEAAKMV
P62495	335	NLDIMRYVLHCQGTEEEKILY
Q921F2	173	SQRHMIDGRWCDCKLPNSKQS
Q9Y536	62	CFHRIIPGFMCQGGDFTRHNG
Q8CI94	373	DKAWEITKKTCAYTNHTVLPE
Q3UZY0	566	HHSGLLRRAFCIWKESTQGFR
Q9WUM4	456	KEIKSIKETICSQDERISKLE
P69905	105	DPVNFKLLSHCLLVTLAAHLP
Q8C7H1	181	LSVLAVDPSSCTSGGSLLGDK
P49327	1127	FCFTPHTEEGCLSERAALQEE
O14980	528	VTVIKDLLGLCEQKRGKDNKA
O08600	110	RLRGDGDRSACDFREDDSVHA
P40124	415	PTISINKTDGCHAYLSKNSLD
Q8BZF8	124	AGGIILTASHCPGGPGGEFGV
Q9QUM9	78	HLFKITESIGCVMTGMTADSR
Q9WUM4	39	VSRVTWDSSFCAVNPRFVAII
P20810	408	AAPAPVSEAVCRTSMCSIQSA
P05976	181	LMAGQEDSNGCINYEAFVKHI
P46459	21	CPTDELSLTNCAVVNEKDFQS
P14131	25	RKKTATAVAHCKRGNGLIKVN
O70145	291	MFNGQKGLVPCNYLEPVELRI
P52480	326	RCNRAGKPVICATQMLESMIK
P63323	92	NKKLGEWVGLCKIDREGKPRK
P31948	26	SVGNIDDALQCYSEAIKLDPH

Q91VD9	463	QDIASGRHSFCEVLKDAKKPM
Q9R0H0	199	VLAQLITRGECYGLHAFVVPI
P56399	219	WLNLTDGSILCGRRYFDGSGG
P68366	376	GDLAKVQRAVCMLSNTTAIAE
Q9EQ20	413	IISNVKPSMTCYKEEIFGPVL
P60843	134	GDYMGASCHACIGGTNVRAEV
P10605	319	FFKILRGENHCGIESEIVAGI
P50995	384	TDESKFNAVLCSRSRAHLVAV
P02766	10	XMASHRLLLLCLAGLVFVSEA
P35250	255	FINSENVFKVCDEPHPLLVKE
P53569	374	RALVAAHELLCDKPEEEKALL
P12004	81	NLTSMSKILKCAGNEDIITLR
P27348	134	DYFRYLAEVACGDDRKQTIDN
Q922E4	324	LLNHFKVDLVCHGKTEIVPDR
Q9Z2W0	411	QDLMVRNDSPCGTTIGPILAS
Q8BMF4	483	DFIIKASALACLKVPEANSSW
Q02566	1342	AHALQSSRHDCDLLREQYEEE
Q6P1X6	98	YEAAFPFLSPCGRERNFLRCE
P52825	535	VGELQHMMAECSKYHGQLTKE
A2AJI0	375	RSASASPLTPCSAPRSAHRCT
P62736	287	HETTYNSIMKCDIDIRKDLYA
Q80YD1	251	FEKSNAAGVPCDLVTGEERLT
P54577	250	DVKKKLKKAFCEPGNVENNGV
Q91VD9	92	IEKAPKVVAACAMPVMKGWNI
P50462	25	KTVYHAEEIQCNGRSFHKTCF
P19838	61	KQRGFRFRYVCEGPSHGGLPG
O95816	162	DQKFQSIVIGCALEDQKKIKR
P68404	217	ESKQKTKTIKCSLNPEWNETF
Q00610	926	LACVAYERGQCDLELINVCNE
Q924X2	427	FFVTLDEDSHCYNPDDETSLS
Q60597	566	EEEISKYDKICEEAFTRSKDE
Q05816	43	RKMAAMAKPDCIITCDGNNIT
Q9Z0S1	206	RSHSNQLVTDCISAMNPDTVL
Q71RI9	338	IDIKRMDDPECYFNSLPKELE
P12970	199	RLGHLVHRKTCTTVAFTQVNS
P80313	511	NALTAASEAACLIVSVDETIK
Q05586	798	LDKTWVRYQECDSRSNAPATL
P30050	162	IDDINSGAVECPASXXXXXXX
Q62159	159	NRISAFGYLECSAKTKEGVRE
Q9ER60	731	KECVCKIASDCSLPRWHMHDF
Q9CQV5	120	LKRRANQVDICALVLRQLPAH
P29474	976	DGLGPLHYGVCSTWLSQLKPG
P04247	67	KGSEDLKKHGCTVLTALGTIL
P52272	676	WKMLKDKFNECGHVLYADIKM
Q9UQM7	280	WISHRSTVASCMHRQETVDCL
Q8NHW5	119	AAARAGAIAPCEVTVPAQNTG
Q8BGX2	52	GALLRDYAEACGDAAAAARAR
Q99LC3	67	SRVITVDGNICSGKNKLAKEI
Q9CQ60	78	ASFARWTLGFCDERLVPFDHA
P17751	268	IYGGSVTGATCKELASQPDVD
P05202	106	YLPIGGLAEFCKASAELALGE
Q8QZS1	335	LIMEYRITQACMEGHDFHEGV

Q3TCJ1	237	VEKSERVVESCQAEVNKLRRQ
Q9WUB4	18	SVKIAPGAVVCVESEIRGDVT
Q9CZY3	144	MKLPQPPEGQCYSNXXXXXXX
P97351	96	LITEDVQGKNCLTNFHGMDLT
P62987	91	SLRQLAQKYNCDKMICRKCYA
Q8BZF8	60	SSIDLRDRQGCTMVVGSDGRY
Q15149	3667	NLTYRQLLERCVEDPETGLRL
P12268	140	VFEAKARHGFCGIPITDTGRM
P41216	298	MITHQNIINDCSGFIKATESA
Q61686	160	LVLAKEANVKCPQIVIAFYEE
P17182	389	TFIADLVVGLCTGQIKTGAPC
Q91ZA3	359	RLQVEHPVTECITGLDLVQEM
Q96AG4	48	LPKATILDLSCNKLTTLPSDF
Q8BWF0	422	LLSNVTRDMLCITEETFGPLA
P17710	214	LPVGFTFSFPCRQSKIDEAVL
O55143	447	VGEATETALTCLVEKMNVFDT
P21333	2543	VFVDSLTKATCAPQHGAPGPG
Q9CXT8	481	IERLPDFNQICSNMRWIRDXX
P68040	153	QDESHSEWVSCVRFSPNSSNP
P59017	136	QPVTYEAYRECTVETAVHASG
P38646	487	DGQTQVEIKVCQGEREMAGDN
P10599	73	QDVASECEVKCMPTFQFFKKG
Q16665	800	SGLPQLTSYDCEVNAPIQGSR
Q8VE95	68	DDSKMKNFITCFKDLQFLVTF
P34914	120	IALKKKGFTTCIVTNNWLDDG
Q91YP0	273	YSDRISELSGCNPDPQIVPFR
P17710	879	CDDSILVKTVCGVVSKRAAQL
P26039	286	ERKIFQAHKNCGQMSEIEAKV
O88844	363	SFFAKALEDVCIETIEAGFMT
Q05D44	631	NTEKLRAPIICVLGHVDTGKT
Q92879	62	SQNPPQSKGCCFVTFYTRKAA
P63244	168	PNSSNPIIVSCGWDKLVKVWN
P48962	257	ADIMYTGTLDCWRKIAKDEGA
Q8BZB2	7	XXXXMEPKAPCPAAVPSEERK
Q8R0N6	260	TAIPYSMRSPCPSNPIQRPAY
O70468	784	PKISNVGEDSCTVQWEPPAYD
Q9CPY7	313	RADMGGAATICSAIVSAAKLN
Q9Z1Q9	478	YRSTRLVNWSCTLNSAISDIE
Q9DCM0	170	GCGRTDFQQGCAKTLYHSVHE
Q8CFE6	400	PIRSSVTHLLCPTKEFSWLRH
P61962	109	RVGETETRLECLLNNNKNSDF
Q9DA03	97	KDLLTENVPYCDAPTQKQXXX
P56375	29	YEVFGTVQGVCFRMYTEGEAK
O08553	439	LEYNIFEGMECRGSPLVVISQ
P68871	94	GTFATLSELHCDKLHVDPENF
O43390	292	PDDKKKNRGFCFLEYEDHKSA
Q07417	246	SSTANLIFEDCRIPKENLLGE
P29474	1114	LCLERGHMFVCGDVTMATNVL
Q9EQ20	368	LITPQAKERVCNLIDSGTKEG
Q9D6M3	52	GQRMYASMSDCLIKTIRSEGY
P05202	295	GERVGAFTVVCKDAEEAKRVE
P98170	303	GEGDKVKCFHCGGGLTDWKPS

P27695	93	WVKEEAPDILCLQETKCSENK
P47754	141	YVKEHYPNGVCTVYGKKVDGQ
Q5SFM8	49	DKPEKELKAFCADQLDVFLQK
P52480	49	PITARNTGIICTIGPASRSVE
Q99NB1	178	SPLAVAAMLACARIGAIHTVV
Q8BH64	138	FGNTFLNRFMCAQLPNQVLES
P60843	131	MALGDYMGASCHACIGGTNVR
Q8BWM0	382	APSVHHVNPSCKDXXXXXXXX
Q8R143	63	SCLWCNENKACMDYPVRKILP
O09131	90	GHLVTESVITCEYLDEAYPEK
Q5M6W3	326	ISLGEYERAACFAANSPKRIL
Q99536	50	AAAASPPLLRCLVLTGFGGYD
Q9EQ20	149	FRGLQVVEHACSVTSLMLGET
P47757	206	QMEKDETVSDCSPHIANIGRL
P13639	290	PEGKKLPRTFCQLILDPIFKV
P47712	151	CPDLRFSMALCDQEKTFRQQR
Q9CQA3	70	MQTYEVDLNKCGPMVLDALIK
Q80YD1	418	ARKFNDPNDPCKILVATDAIG
Q91WK1	114	VADVDMSRDSCVGADDRSWVF
Q15366	158	IAGIPQSIIECVKQICVVMLE
P17742	161	KTSKKITISDCGQLXXXXXXX
P35247	40	TMPSACTLVMCSSVESGLPGR
O35309	183	QTRDKLELSFCKSRNGGGEVE
Q9CZR8	148	QQVALGTMAHCQNLTDRLSTY
P48643	407	EAKRSLHDALCVIRNLIRDNR
P50462	123	KFGESEKCPRCGKSVYAAEKV
Q09161	483	FSALCPANPTCIYKYGDESSN
P97351	111	HGMDLTRDKMCSMVKKWQTMI
Q8BGK2	137	SGFGAATKAMCIGMRYWKPER
P53395	333	PILNASVDENCQNITYKASHN
P10518	203	VMSYSAKFASCFYGPFRDAAQ
Q62095	222	IIKEKRDLMACAQTGSGKTAA
P61106	26	IIGDMGVGKSCLLHQFTEKKF
P49717	604	TLSIAKAGIICQLNARTSVLA
Q99NB9	933	KRVKPYLPQICGTVLWRLNNK
P98170	351	NIHLTHSLEECLVRTTEKTPS
P41216	626	RGLQGSFEELCRNKDINKAIL
Q99L04	177	KAACDRLAADCAHELRRHGVS
P53396	229	AKVDATADYICKVKWGDIEFP
P78527	1507	RQCLPSLDLSCKQLASGLLEL
P78527	1499	KGIAPGDERQCLPSLDLSCKQ
P00558	50	IKAAVPSIKFCLDNGAKSVVL
P26641	194	FPNTNRWFLTCINQPQFRAVL
P06801	164	ERILGLGDLGCNGMGIPVGKL
Q8BMS4	156	LSGMKILDVGCGGGLLTEPLG
Q8VDC0	625	DIDFTGPAPVCAKTKEKLEVT
P17563	8	XXXMATKCTKCGPGYSTPLEA
Q9WV60	317	PRTPPEAIALCSRLLEYTPTA
P62913	150	SIADKKRRTGCIGAKHRISKE
Q60932	140	GYKREHINLGCDVDFDIAGPS
Q00987	77	YDEKQQHIVYCSNDLLGDLFG
Q78J03	162	DGPKPTGQRFCINSVALKFKP

Q9CQN1	263	TKIIIHLKSDCKDFASESRVQ
Q9WTZ1	61	AICRVQVMDACLRCQAENKQE
Q9D517	254	LYGKKYEADMCVRRFPLEDIP
P43897	71	FVNCKKALETCGGDLKQAEIW
Q02566	1750	QTEVEEAVQECRNAEEKAKKA
P51787	445	EKMLTVPHITCDPPEERRLDH
P21817	35	ATVLKEQLKLCLAAEGFGNRL
Q6IFX4	453	GPPSRILVKICTITKEIKDGK
Q9WU65	381	YWEPSARGIICGLTQFTNKCH
O95801	367	VKALTPAFLVCVGSSPFCKNF
Q9Z2I9	384	LVNIFGGIMRCDVIAQGIVMA
P30999	533	WESVLTNTAGCLRNVSSERSE
P63242	73	IFTGKKYEDICPSTHNMDVPN
P54310	743	CRETRQATEFCVQRIRLILTP
Q8R1G2	222	HGFVHRKREDCSPADKPYIEE
P21980	524	VSYNGILGPECGTKYLLNLNL
P50462	168	NVTDKDGELYCKVCYAKNFGP
O55143	524	KGAPEGVIDRCTHIRVGSTKV
P61620	13	IKFLEVIKPFCVILPEIQKPE
Q8CI94	446	DCKRINMAHLCVIGSHAVNGV
P10599	62	VIFLEVDVDDCQDVASECEVK
Q8K411	627	LFCSVLTKLGCGILNYREQAQ
Q9WV55	179	DTETRKLMEECKRLQGEMMKL
Q8BIJ6	110	QRERKVKTEFCLHDGPPYANG
P12382	351	NQSVRLPLMECVQVTKDVQKA
O95861	243	ASAYVFASPGCKKWDTCAPEV
P11499	366	YVRRVFIMDSCDELIPEYLNF
P51660	189	AIEGRKNNIHCNTIAPNAGSR
O08992	240	RNGLLTDHHICEINGQNVIGL
Q8BKY8	276	SISFTKTTFECTDYDLRQLVV
P10649	115	MDTRMQLIMLCYNPDFEKQKP
Q3B7Z2	461	RLEENGYRSICEQVSHHPPAA
O08715	352	ATQPKGKEESCVPASQETSLG
P10518	75	LRPLVEAGLRCVLIFGVPSRV
P32322	262	SLLINAVEASCIRTRELQSMA
P85094	84	AQGIRPVSKTCFSMVPALQKE
P62993	32	GDILKVLNEECDQNWYKAELN
Q8K2C6	293	DRFRFHFPGPCGKTLPEALAP
P27659	114	TVFAEHISDECKRRFYKNWHK
Q9D0K2	504	TVDDIKKSTGCDFAVSPNLMP
Q3ULD5	431	DGAKMVAAVACAKVPKITVII
P35505	315	GEGMSQAATICRSNFKHMYWT
P70695	331	SPEDVQEYLSCVQRNQAGRXX
P26039	2480	LVKAAQKAAACEDQENETVVV
O95372	56	TIRLPHVKYICPHAPRIPVTL
P21266	208	IAAYLQSDQFCKMPINNKMAQ
Q99KI0	385	LDIRVGLIGSCTNSSYEDMGR
P50396	202	YRTDDYLDQPCLETINRIKLY
P84075	185	SDPSIVRLLQCDPSSASQFXX
Q9H9Q2	110	TIVSLASRMKCIPYSVLLKDL
P62281	60	AIEGTYIDKKCPFTGNVSIRG
O43395	37	EPTVVTAALNCVGKGMDKKKA

P10809	237	YFINTSKGQKCEFQDAYVLLS
P07237	56	VEFYAPWCGHCKALAPEYAKA
P84091	337	PLNTSGVQVICMKGKAKYKAS
Q99MR8	505	SHSTIAKESVCQAALGLILKE
P07900	481	GDEMVSLKDYCTRMKENQKHI
P28659	177	AMHQAQTMEGCSSPMVVKFAD
O76021	197	SKNLSREINDCIGGTVLNISK
P85094	136	GLQVHVVVDACSSRSQVDRLV
Q9WTX5	120	LDIKGLLDVTCKTVANMIKGK
Q8BG51	572	HKMPPPQAFTCNTADAPSKDI
P14866	260	SLNGADIYSGCCTLKIEYAKP
Q8BH59	563	GQTTYSGVVDCFRKILREEGP
P62889	85	SGNNIELGTACGKYYRVCTLA
Q9H0F7	148	VTSVKVSQLLCLENIKDKPWH
P05213	315	CDPRHGKYMACCLLYRGDVVP
Q9QYG0	321	LQGMGYMASSCMTRLSRSRTA
Q9CWJ9	363	ILSKKKNGNYCVLQMDQSYKP
Q8K2B3	238	NGECRGVIALCIEDGSIHRIR
P30416	396	NKAAKTQLAVCQQRTRRQLAR
Q8BLF1	345	EALPKTYILTCEHDVLRDDGI
P58252	693	FQWATKEGALCEENMRGVRFD
Q99MR8	186	GYHGKDQSDQCLREHAGKIGY
Q7TNV0	165	KFRNAMLKSICEVLDLERSGV
P00492	106	TVDFIRLKSYCNDQSTGDIKV
P29218	184	MVLSNMEKLFCIPVHGIRSVG
P23919	31	TQSRKLVEALCAAGHRAELLR
Q91ZA3	394	DIPISGWAVECRVYAEDPYKS
A3KMP2	124	MFAKGNFPRACDLWEQILRDH
Q92769	274	ADSLSGDRLGCFNLTVKGHAK
P13639	41	GKSTLTDSLVCKAGIIASARA
P50991	295	NLVKQIKKTGCNVLLIQKSIL
Q9QYG0	371	GPPGHTMEVSCXXXXXXXXXX
Q9JHI5	134	GLSYGAHSNLCVNQIVRNGNE
P70296	133	LVYEQEQPLSCDEPILSNKSG
Q8K2B3	266	TGGYGRTYFSCTSAHTSTGDG
Q6P8J7	90	MTPSGYTLDQCIQTGVDNPGH
Q922B1	264	EHRLRSVAFPCISTGVFGYPN
P70398	787	VVIQSNDDIACRAIDLLKEIY
P26883	23	GRTFPKRGQTCVVHYTGMLED
P21126	13	LTVKALQGRECSLQVAEDELV
P50579	436	SKYLMALKNLCDLGIVDPYPP
Q3ULJ0	216	KNIVAVGAGFCDGLRCGDNTK
O35643	863	ENEAQFQIRDCPLNTEAASNK
Q5U458	494	KVIDVTVPLQCLVKDSKLILT
P45952	244	GKKELNMGQRCSDTRGIAFED
P17751	117	DPKIAVAAQNCYKVTNGAFTG
P97494	501	CKGGNAVVDGCSKAQSSSEPA
P45880	76	TGTLETKYKWCEYGLTFTEKW
Q7TPV4	361	STYVGTFLEGCQDDPKRQLTM
P46459	250	FPPEIVEQMGCKHVKGILLYG
Q8BP47	522	WYTDQRKYGTCPHGGYGLGLE
Q9CQM9	148	RLKKLTHAAPCMLFMKGTPQE

Q9EPV8	6	XXXXXMIEVVCNDRLGKKVRV
Q78IK4	152	LGLATLGATVCYPAQSVIIAK
P14206	163	PLRYVDIAIPCNNKGAHSVGL
Q99L13	250	AKILNMSSGRCWSSDTYNPVP
Q8VCI5	229	QQQHSVMVKICEQFEAETPTD
Q99832	450	KALEIIPRQLCDNAGFDATNI
P28482	166	LLNTTCDLKICDFGLARVADP
O00299	191	LPKLHIVQVVCKKYRGFTIPE
O08529	405	DEDEEDGERGCTFLVGLIQKH
Q8C460	285	LKKAYSFAMGCWPKNGLLDMN
P62913	72	IRRNEKIAVHCTVRGAKAEEI
Q811U4	681	QEMATTFARLCQQVDVTQKHL
P12382	708	RVFANAPDSACVIGLRKKVVA
O15519	259	KPLGICLIIDCIGNETELLRD
Q7TPD0	346	LSTPDSQSLRCDLIRYICGVV
P58404	720	LRLWSLDNKTCVQEITAHRKK
P63276	35	GNDFHTNKRVCEEIAIIPSKK
P68366	347	TKRSIQFVDWCPTGFKVGINY
Q6PDN3	1291	GKVAGTQPITCKWMKFRKQIQ
Q8BTM8	623	VEGPSQAKIECDDKGDGSCDV
Q8BXG3	410	TLLQSKLKEKCNMTRDMHAAV
P85094	21	ILPESSILFLCDLQEKFRPSI
B1AR13	119	TCKATQRPPYCDGTHKSEQVQ
P17182	399	CTGQIKTGAPCRSERLAKYNQ
Q14003	370	TFEFLMRITFCPDKVEFLKSS
P40227	406	RAVKNAIDDGCVVPGAGAVEV
P29474	201	GKLQVFDARDCRSAQEMFTYI
Q9CQ62	268	FEKEMIDRIPCGRLGTMEELA
Q91Z53	29	GRAALAQAADCEVEQWNSDDP
Q64727	545	GPYRQDLLAKCDRVDQLTAQL
P10637	614	PVDLSKVTSKCGSLGNIHHKP
P31749	224	KYSFQTHDRLCFVMEYANGGE
Q9DCW4	42	DGVKHSMNPFCEIAVEEAVRL
P13489	248	KLGDVGMAELCPGLLHPSSRL
P09411	380	IIGGGDTATCCAKWNTEDKVS
O70250	23	ESLWNQENRFCGWFDAELSEK
Q9NR33	84	FVETIAKDAYCCAQQGKRKTL
Q8K411	556	TQQSKHQDASCLPALKVSDIE
Q00610	491	RANVPNKVIQCFAETGQVQKI
Q7TMF3	92	VPPEWHRWLHCMTDDPPTTNP
P63244	138	TIKLWNTLGVCKYTVQDESHS
Q8BIJ6	883	PGLEEAVESACAMRDSFLGSI
Q99P88	799	YQALALWKLLCEHQFSVIVGE
O35737	22	VVKVRGLPWSCSADEVQRFFS
P28659	150	LRGPDGLSRGCAFVTFTTRTM
P36873	127	NFFLLRGNHECASINRIYGFY
Q8BIJ6	465	KEENIVHSYPCDWRTKTPVLI
Q8BZA9	114	RAMAKAAGEECPMFTPPGGET
Q6A068	769	EDSAIPRRLECLKEDVQRQQE
Q8CI94	143	GNGGLGRLAACFLDSMATLGL
O54931	416	TQAAKEQKAPCVSESQSAGAG
P14824	96	IVNLMRPLAYCDAKEIKDAIS

O70370	281	YKSGVYDDPSCTGNVNHGVLV
P29474	212	RSAQEMFTYICNHIKYATNRG
P58252	751	MEPIYLVEIQCPEQVVGGIYG
Q8K0S0	261	RDRLPLLDIACNKFLTCSVED
P34914	78	DESYRKSSKACGANLPENFSI
P21981	553	LRILYEKYSGCLTESNLIKVR
Q91Z53	123	ELAVSLLLTTCRRLPEAIEEV
Q9CPY7	462	NLGKYRSAGACTAAAFLREFV
O55234	111	LGTMAGGAADCSFWERLLARQ
Q64462	250	TPHGRRFLRACQIAHDHTDHV
P52657	98	IKVDKVKIVACDGKNTGSNTT
P28474	240	AKAKEFGASECISPQDFSKSI
P97823	211	KIYEGMMHSSCQQEMMDVKHF
P42574	163	GKPKLFIIQACRGTELDCGIE
O55137	14	TLNLEPSGRSCWDEPLSIAVR
Q9CQW1	66	RASVKEQEYLCHVYVRSDSLA
Q8BU88	166	GRFGIMEKVYCHYFVKLVEGP
Q9EPV8	18	DRLGKKVRVKCNTDDTIGDLK
P62746	107	EKWVPEVKHFCPNVPIILVAN
O70433	132	WHETCFTCQRCQQPIGTKSFI
P54822	27	LAARYASREMCFLFSDRYKFQ
O14920	179	YAKELDQGSLCTSFVGTLQYL
Q9D2G2	246	AQRLKEAQNTCAMLTTFNEVD
Q8K2C6	124	PNPGHLAIAQCEARLRDQGRR
Q62426	64	FIKVDVGGDKCVHLRVFQPLP
P09411	316	GIPAGWMGLDCGTESSKKYAE
Q14139	465	SRLLTFNPTYCALKELNDEER
Q99MK8	120	DSYIMKELLACSHPFSKNATE
P70168	223	SERHFIMQVVCEATQCPDTRV
P53811	94	HEKAWNAYPYCRTIVTNEYMK
Q9CQH8	23	SPSEYHYMKVCLEFQEHGVGL
P00403	196	ATRPGVYYGQCSEICGANHSF
Q9Z2Z6	283	MIRAFPANAACFLGFEIAMKF
P70168	585	IQFNDLQSLLCATLQNVLRKV
P21980	545	EPFSEKSVPLCILYEKYRDCL
Q9WUM4	330	MPKRGLDVNKCEIARFFKLHE
Q8BWT1	179	AAKYNISREDCDRYALQSQQR
Q8VE38	68	RREVMAAAKVCEITHESPSVK
P30041	91	AWSKDINAYNCEEPTEKLPFP
Q8BG51	535	KQHFMDSRIPCLIVAAKSDLH
P35754	79	TVPRVFIGKDCIGGCSDLVSL
P85094	206	LFQGQSPLTSCXXXXXXXXXX
Q99961	147	QNFIDPLQNLCEKDLKEIQHH
O55023	183	RIVLSNMEKLCSIPIHGIRSV
Q9CQJ8	4	XXXXXXXMAFCAPPAYLTHQQ
O75822	207	KKITNSLTVLCSEKQKQEKQS
Q9UL62	553	ETRAIDEPNNCKGIRCEKQNN
P55769	30	KKLLDLVQQSCNYKQLRKGAN
Q62234	406	GREGETMSLGCRVVITPEIKH
Q8VEK3	311	ASYGVSKGKVCFEMKVTEKIP
P60520	15	FKEDHSLEHRCVESAKIRAKY
Q9CQ62	86	TTFLSTLGAQCVIASRNIDVL

P61163	34	GFAGDQIPKYCFPNYVGRPKH
P50570	86	SKTEHAEFLHCKSKKFTDFDE
P56391	65	EWYRRVYKSLCPVSWVSAWDD
Q60870	14	RFDRFLHEKNCMTDLLAKLEA
P17174	46	LGVGAYRTDDCHPWVLPVVKK
Q8K2B3	654	VIDKTLNEADCATVPPAIRSY
Q8VDQ1	187	LLGCSRVVGICGTQEKCLFLT
P21817	1302	LPVQFHQHFRCTAGATPLAPP
Q99L13	210	AVGTGQSAKICNNMLLAISMI
Q8K2M0	321	MTPAGLAFFQCRWDDSVTHTF
Q9EP69	445	KNAWADNANACAKQYAGTGAL
Q9DBF1	70	GGRGEVITTYCPANNEPIARV
P13707	329	FPLFTAVYKVCYEGQPVGEFI
Q8CI94	437	RRMSVIEEGDCKRINMAHLCV
P22314	632	QDPPEKSIPICTLKNFPNAIE
P62879	25	RNQIRDARKACGDSTLTQITA
Q9CZ13	380	FFLQGQWMRLCTSATESEVTR
Q9NQC3	1101	YSNSALGHVNCTIKELRRLFL
P08752	66	IHEDGYSEEECRQYRAVVYSN
P31327	600	YALGGLGSGICPNRETLMDLS
Q62261	1389	ANKAELFTQSCADLDKWLHGL
Q9D0M3	139	YVAYRHLVGVCYTEEEAKALA
Q9H9Q2	240	EMEQQLAERECPPHAEQRQPT
Q60932	245	AAKYQVDPDACFSAKVNNSSL
P41567	69	LVKAFKKKFACNGTVIEHPEY
Q6P3A8	297	SMAQEKLGVSCEVIDLRTIVP
P45984	163	KPSNIVVKSDCTLKILDFGLA
O35678	32	LVNADGQYLFCRYWKPSGTPK
Q99JX4	125	KNTPVRYTVYCSLIKVAASCG
P21283	225	VLSEDQDSYLCNVTLFRKAVD
Q8K1A5	44	GSKDHLNEKPCAEAGSARTSL
P19253	38	LLGRKVVVVRCEGINISGNFY
Q99LD8	262	LSDVTLVPVSCSELEKAGAGL
P12979	65	LGTPEHCPGQCLPWACKVCKR
Q3UQ84	585	QYKGPAGTPECPVLIHRAVLG
Q9CR24	71	KGEVSFPGGKCDPDDQDVIHT
Q99K01	491	CIQSKLPVLTCTLQLREEFKQ
P99028	65	VSSRSQTEEDCTEELFDFLHA
Q91VD9	564	CITRQDLPKDCFIVYQGHHGD
O08573	138	HLVDTIAVSGCLKLSFITFQN
Q13257	106	VLERWQFDIECDKTAKDDSAP
Q9CZC8	324	VKLVPKAQSPCFGDDDPAKKE
Q9D0F9	374	NLMDASKLSLCGEESFGTGSD
P31943	267	SDRFGRDLNYCFSGMSDHRYG
P23975	351	RDALLTSSINCITSFVSGFAI
P04406	152	NSLKIISNASCTTNCLAPLAK
O35459	186	DLVSACDIRYCTQDAFFQIKE
Q15185	76	HKRTDRSILCCLRKGESGQSW
P62888	52	KAKLVILANNCPALRKSEIEY
Q13144	618	MDSPLDSSRYCALLLPLLKAW
Q9CZ42	306	SPLLVAAWGACTLTRECNRQA
Q8BIJ6	311	PWTIPANQAICYMPEAKYAVV

Q9CQL4	93	YPAFIVNLIKCQVELNRKVLV
P16858	282	LGYTEDQVVSCDFNSNSHSST
Q9CQE3	100	RVIDPVTGKPCAGTAYLESPL
P18206	85	PPAFIKVENACTKLVQAAQML
P05063	178	ANVLARYASICQQNGIVPIVE
Q9WVQ5	96	PASKKLKKSQCTPLFMNAYTM
P58281	551	TRNLSLAVSDCFWKMVRESVE
Q9QYJ0	308	YPPGKVIEPGCVRVVRGEGMP
Q9CZR8	314	VTVVDFVRFECGEDEQVAEAE
Q9JHW2	97	EEDAGKLYNTCSVFGPDGSLL
Q9CR00	81	QVRTARHNIICLQNDHKALMK
Q61233	336	REKDDIQRAECMLQQAERLGC
P84086	105	TRPKKAIPAGCGDEEEEEEES
P99028	35	VDPLTTVREHCEQLEKCVKAR
P97443	74	RCGQCKFAHYCDRTCQKDAWL
P07814	92	LEFSATKLSSCDSFTSTINEL
Q9Y2S7	143	YYQVLIDARDCPHISQRSQTE
A6H611	446	FQRANKPQQDCHFTIRGGRLK
P14866	261	LNGADIYSGCCTLKIEYAKPT
P14873	1936	IKTPEDGGYTCEITEKTTRTP
Q9CX34	49	EQNPDDAQYYCQRAYCHILLG
P22102	298	EFNCRFGDPECQVILPLLKSD
Q9DCB8	79	FLRLQVEGGGCSGFQYKFSLD
P55072	572	IFDKARQAAPCVLFFDELDSI
Q62312	76	NGGAVKLPQLCKFCDVRLSTC
Q922Q8	140	DCLDEKQCKQCANKVLQHMKA
Q6NSR8	284	KTTMPGMKRDCGGAAAVLGAF
Q922B1	114	SDKQREEHYFCKDFIKLKKIP
Q8VDN2	249	TRNIAFFSTNCVEGTARGIVV
Q9CR62	184	EEGVPTLWRGCIPTMARAVVV
Q8R3V5	267	VKSQTTYYAQCYRHMLDLQKQ
P55036	87	TVQPKGKITFCTGIRVAHLAL
O43765	129	ELNPANAVYFCNRAAAYSKLG
P40937	238	GKVTEETVYTCTGHPLKSDIA
P55072	535	TLLAKAIANECQANFISIKGP
Q8CGY6	519	KLAKQCRKWLCNTAIDTRTRR
Q05816	87	TADGRKTETVCTFQDGALVQH
Q3U1J4	378	ERQGQGQLVTCSGAFKEGSLR
Q8VHX6	729	VIPNGDGTFRCSYVPTKPIKH
Q60597	331	VIRKELEQIFCQFDSKLEAAD
P47857	334	LLEGTPDTPACVVSLSGNQAV
O35459	91	NRAFWRELVECFQKISKDSDC
Q9D0S9	75	PADILYEDQQCLVFRDVAPQA
Q99J39	401	QALQGPLMRLCAWYLYGEKHR
O94760	274	ELEKVDGLLTCCSVLINKKVD
Q8R2L5	101	CIYGRHITGLCGKKQREITKA
Q00610	934	GQCDLELINVCNENSLFKSLS
Q5UIP0	312	DNFALNPDILCSAKRLKLLMQ
Q8BTM8	2476	IDGPSKVKMDCQECPEGYRVT
O55125	83	KIQFHNVKPECLDAYNSLTEA
P62857	27	VLGRTGSQGQCTQVRVEFMDD
Q8R127	238	KLKRRWPVSYCRELNSYSIPF

Q9CXT8	248	RIVLAAAGGVCHNELLELAKF
P13010	235	YSFSESLRKLCVFKKIERHSI
Q8R1F1	154	SKSGSTPILKCPTQFPLILWH
Q80X90	2115	APAVATVGSICDLNLKIPEIN
P51174	166	KFIPQMTAGKCIGAIAMTEPG
P14866	581	NGPYPYTLKLCFSTAQHASXX
P97372	33	NLFQEADDFLCTFLPRKIISL
P50396	282	GEVARCKQLICDPSYIPDRVQ
Q92600	91	TLTAHQSNRVCNALALLQCVA
P12979	70	HCPGQCLPWACKVCKRKSVSV
O88587	234	AYVRGSSSFECTHYSSYLEYM
Q9D051	306	WPQFGVGAEICARIMEGPAFN
P29474	991	SQLKPGDPVPCFIRGAPSFRL
P08207	62	VDKIMKDLDQCRDGKVGFQSF
Q3V4B5	37	KLGMAVSSDSCRSLKYPYVAV
Q9JKX6	75	PVLQRTLHHECVILVKQFRPP
Q9DAS9	43	SKASADLMSYCEEHARSDPLL
Q8K411	780	KYLLNCDNMRCSVNATPQQMP
Q99JW4	22	RCKGGFAPAECIVNSNGELYH
O88342	170	YRLATGSDDNCAAFFEGPPFK
P42772	74	LLLLHGAEPNCADPATLTRPV
P06745	133	NRVLDKMKSFCQRVRSGDWKG
Q8CIM3	37	RACVGLKRLGCPRGVYSPLAH
Q5SW19	665	SEDSIGPEAGCEEEGSSVSGL
Q9D7X3	124	AHKNGRVLVHCREGYSRSPTL
P45376	187	KYKPAVNQIECHPYLTQEKLI
P47738	388	SGQQEGAKLLCGGGAAADRGY
P47857	709	RIFANTPDSGCVLGMRKRALV
P55060	387	SDIDTRRRAACDLVRGLCKFF
O75828	226	RKADRILVNACCPGPVKTDMD
P98078	138	VTDNRAFGYVCGGEGQHQFFA
O08528	368	RLGLSPLQEDCVATHRICQIV
Q8VDN2	428	WFALSRIAGLCNRAVFQANQE
P48725	2650	KTLKHHTQKGCVLNRQSKSSL
P08249	89	GYLGPEQLPDCLKGCDVVVIP
O09131	32	GQIRVYSMRFCPFAQRTLMVL
Q9DBL1	261	NKMGIRASSTCQLTFENVKVP
Q8K274	24	VKATGHSGGGCISQGQSYDTD
Q7L8L6	670	KAAVPLGGFLCNVADKSGAME
Q9CR21	140	IPDIDAEKLMCPQEIVDYIAD
Q9Z2U0	63	LQDERTVRKICALDDNVCMAF
P70670	1866	LLVSPAKGSDCLHSPKGPVGS
Q02257	372	TSNSPRLVQNCLWTLRNLSDV
Q71FD7	334	ECMGRNFHENCYRCEDCSVLL
Q923E4	390	FNQVVPRCPRCPADEPLAIMK
Q9JJX8	38	AIGKGSFGKVCIVQKRDTKKM
P80313	158	KVEQRKMLEKCAMTALSSKLI
Q8BUV3	284	QATSRLSTASCPTPKQIRRPD
Q8NBI5	226	PLPPNYSYGLCPGNGTTKEEK
P13639	728	GGQIIPTARRCLYASVLTAQP
O08528	794	TKFLSQIESDCLALLQVRAIL
P82933	330	VDRLGKHDVTCTVSGGGRSAQ

Q99KI0	126	PKVAVPSTIHCDHLIEAQVGG
Q91VT4	5	XXXXXXMDKVCAVFGGSRGIG
P78527	25	LQETLSAADRCGAALAGHQLI
P58281	375	LRMRKNVKEGCTVSPETISLN
Q8BFS6	54	GLMKAWSTGNCDAGGDEWGQE
P10649	174	LDQYRMFEPKCLDAFPNLRDF
O00220	336	VTVQSPGEAQCLLGPAEAEGS
P61979	145	YQHYKGSDFDCELRLLIHQSL
Q15527	38	RCILTGHELPCRLPELQVYTR
Q15527	29	RLQTDARKVRCILTGHELPCR
Q91YR7	739	REAYNQGLKKCPHSTPLWLLL
Q924M7	11	MASPRVFPLSCVVQQYAWGKV
Q497Q6	177	QDDEKRTLLQCETGKIYTMKE
P24270	393	VANYQRDGPMCMHDNQGGAPN
P21817	1039	ATKRSNRDSLCQAVRTLLGYG
Q60936	617	HRKMGGSFLICSKLKARFPCK
P14873	2357	PPGLPVYLDLCYIPNHSNSKN
Q99L47	208	EEAAHDLALACKLDYDEDASA
Q15366	54	GARINISEGNCPERIITLAGP
Q8BMF4	290	TRDVPLGAPLCIIVEKQEDIA
O94760	222	LTVPDDIAANCIYLNIPNKGH
P06213	1083	NEASVMKGFTCHHVVRLLGVV
Q62433	289	TKTTLLKMADCGGLPQISQPA
Q9CQ65	130	RPQTFYDGSHCSARGVCHIPM
Q9CZB0	70	KWSLPMALSVCHRGSGIALSG
P17987	236	PKRIVNAKIACLDFSLQKTKM
P08551	323	LKAKTLEIEACRGMNEALEKQ
Q9D880	249	RDPARVVVVDCKKEAFRLQPF
Q8BGC4	55	FHEAVTLRRDCPVPLPGDGDL
Q64105	160	SKTVVNISSLCALQPYKGWGL
Q9JHK4	99	VKAELGFLESCLRVNPKSYGT
Q8JZN7	545	APPGLSPAEFCRRHRLPAPAS
P22460	331	ADPFFIVETTCVIWFTFELLV
P17182	119	NAILGVSLAVCKAGAVEKGVP
P53041	404	GRSISKRGVSCQFGPDVTKAF
Q9CZ13	453	DICSKYFYDQCPAVAGYGPIE
Q91YT0	142	RHDPHKLVEGCLVGGRAMGAR
Q60749	19	SRLTRSSGRSCSKDPSGAHPS
P62333	193	LARAVASQLDCNFLKVVSSSI
O55222	346	SMADVKFSFQCPGRMYAPAWV
P03899	39	LYSEKANPYECGFDPTSSARL
Q9D6Y9	309	GLNMFDGTDSCYFHSGPRGTH
Q99L85	95	EGPLKALRSMCKRTDHGSVTI
P17563	461	LGPALAHELRCPGGDCSSDIW
P61758	113	TRFLLADNLYCKASVPPTDKM
Q9CQR4	40	LVSAAPEKLICEMKVEEQHTN
P26443	112	VRGILRIIKPCNHVLSLSFPI
P47738	181	SYTRHEPVGVCGQIIPWNFPL
Q9D024	215	YNLWCSGRVCCEGMLIQLRFL
P46471	377	DIRFELLARLCPNSTGAEIRS
Q924X2	305	PVMALGMVPMCSYQMERMFNT
O09161	53	KQMLKRYDLLCLYYHEPVSSD

P08249	93	PEQLPDCLKGCDVVVIPAGVP
Q91VT4	115	HTNLLGSMLTCKAAMKTMIQQ
P29474	99	QQDGPCTPRRCLGSLVFPRKL
Q99JY0	436	SLGHPFGATGCRLVMAAANRL
Q921G7	265	LYKKFDLRASCDAQTYGIGLK
P61971	38	QLGAIYIDASCLTWEGQQFQG
Q8WTY4	92	EMARILRPGGCLFLKEPVETA
P19367	813	ILQQLGLNSTCDDSILVKTVC
P97478	137	ALLGKEGAMACTVAVEESIAN
Q02566	697	DNPLVMHQLRCNGVLEGIRIC
P20618	89	VIGCSGFHGDCLTLTKIIEAR
Q9CPY7	376	EGRLILADALCYAHTFNPKVI
O43447	131	GCQFFITCSKCDWLDGKHVVF
Q8TAT6	188	GKFVALENISCKIKSGCEGHL
Q8BH64	356	HHISPGDFPDCQKMQELLMAH
Q9WVQ5	75	ERIQPEDMFVCDINEQDISGP
Q9NX47	46	RGSTKWVHQACLQRWVDEKQR
Q8BHP7	56	TSEGSDCRCKCIMRPLSKDAC
Q61425	201	QKTFESLVDFCKTLGKHPVSC
Q91VD9	367	LLNKVDSDNLCTEEIFPTEGA
Q64105	172	LQPYKGWGLYCAGKAARDMLY
Q9CRB2	18	PEESEAQAEGCSEERTYKELL
P00441	7	XXXXMATKAVCVLKGDGPVQG
P23284	202	KPLKDVIIADCGKIEVEKPFA
P06801	110	IVYTPTVGLACQQYSLAFRKP
Q9WUM3	345	ARFYKLHERKCEPIVMTVPRK
Q9D6R2	331	FDHAAKIEAACFATIKDGKSL
Q9CXA2	39	GEPLRIVHAGCPEVAGPTLLA
P62753	12	KLNISFPATGCQKLIEVDDER
Q9WUM5	60	IYIDKNTKIICQGFTGKQGTF
O94927	350	QVLILGLRRCCLWTELKALHD
P36578	208	NRRRIQRRGPCIIYNEDNGII
Q9QUR6	57	NKITVPFLEQCPIRGLYKERM
Q99LC5	155	VRTIYAGNALCTVKCDEKVKV
Q99P30	223	DFDLHDLIPSCERTFLWRYSL
Q92616	1535	AYCAPKQLSSCLPNIVPKLTE
P30556	289	IVDTAMPITICIAYFNNCLNP
Q8BIQ5	150	MFELMKQMKLCVQNSPQEARN
P46660	54	SRSNVASTAACSSASSLGLGL
Q9D7B6	349	QEEREDAVALCSMAKLFATEE
Q3ULD5	167	QEIALQNRLPCIYLVDSGGAN
P17987	357	QAEEVVQERICDDELILIKNT
Q99L04	10	XMVAPMKGQVCVVTGASRGIG
Q96I24	109	QISRIQAESGCKIQIASESSG
P51410	74	RKELATVRTICSHVQNMIKGV
Q13347	144	DNNEPYMKIPCNDSKITSAVW
P47857	114	NLVKRGITNLCVIGGDGSLTG
P52480	358	VANAVLDGADCIMLSGETAKG
Q8TCG1	92	QLAVDIETRDCLQNTYNLNSV
P34914	230	QFPEAPLPVPCNPNDVSHGYV
Q9H9A6	264	KLRFLPEFPSCSLLKELHVGE
Q9CZ13	268	EEDAVPGLTPCRFTGSEIRHR

Q99KQ4	397	LQKLTRDLLNCSFKCSYVVTN
Q16555	504	VPRGLYDGPVCEVSVTPKTVT
P41216	221	LEGVENKLTPCLKIIVIMDSY
Q9DB27	113	KFVLSGANIMCPGLTSPGAKL
Q6PIE5	209	ADLRIISSHGCKVDNSSLTGE
Q8BFR5	222	ETPVIVGSALCALEQRDPELG
Q8VEM8	232	QIPYTMMKFACFERTVEALYK
Q9R112	361	AQSGILDRTMCLIMKNQRPIK
P25205	148	RPKVVRSVHYCPATKKTIERR
Q99MN9	92	MESDMFVEHRCADFGMAADKN
O43143	190	SLPGPKRGVACTQPRRVAAMS
P50990	149	AHEILPNLVCCSAKNLRDIDE
A2ASS6	14760	SEGKIHRLQICDIKPRDQGEY
Q8BVI4	158	GMAKGAVHQLCQSLAGKNSGM
Q9EPL8	477	SELGYMRARACWVLHYFCEVK
P28271	165	EFLKWGSQAFCNMRIIPPGSG
P56501	25	VKFLGAGTAACFADLLTFPLD
Q9CZ30	187	KKLKPEYDIMCKVKSWVIDQK
Q8BVQ5	238	RVSMVGQVKQCEGITSPEGSK
P63104	189	YEILNSPEKACSLAKTAFDEA
Q7TNG8	369	PGSKAYSTDVCVPISRLPEIL
Q78J03	132	ILRRLDTSLGCPRMEVVCKQC
Q60597	956	QKYPNAELAWCQEEHKNQGYY
Q9CQ62	116	KTGNKVHAIRCDVRDPDMVHN
P17987	76	EVEHPAAKVLCELADLQDKEV
P80318	475	SLRAKHTQESCETWGVNGETG
P53811	230	IFTNLHRQLFCWIDKWIDLTM
P35579	896	QEQLQAETELCAEAEELRARL
Q9BUJ2	487	WDVLIQQATQCLNRLIQIAAR
P41216	275	PPEPEDLAIICFTSGTTGNPK
Q99J39	214	PCEVLQKISECEAVHPVKNWM
Q9NPH0	175	NIFRNLESTRCLLAGLFQCQK
Q921G7	117	QIGAHTLSGACLDPAAFKELF
P48039	127	NITGIAINRYCYICHSLKYDK
P13639	567	CLKDLEEDHACIPIKKSDPVV
P26599	250	SLDGQNIYNACCTLRIDFSKL
Q8BP48	174	EEIDHAVHLACIARNCYPSPL
P02730	317	LLHSLEGFLDCSLVLPPTDAP
Q9WUB3	784	VFADYEEYIKCQDKVSELYKN
Q93092	250	NTGEIKALAGCDFLTISPKLL
P15105	183	RDIVEAHYRACLYAGVKITGT
Q922B2	130	VRKVNQKIGSCTQQDVELHVQ
Q8BH95	111	KEMQNRTFQDCYSSKFLSHWD
P54136	369	DGRKIVFVPGCSIPLTIVKSD
Q91VT4	55	ELGGNHLAFRCDVAKEQDVQS
Q60936	638	AMFEEAYSNYCRMKSGLQXXX
Q99P88	1208	LAECKLAVIHCAGYSDPILVH
Q6PB66	112	GLLQRVFESTCSSGSPGSNQA
Q9JLZ3	113	IIRSEVPGIFCAGADLKERAK
P27816	635	PETVTGTGKKCSLPAEEDSVL
P62761	87	DGTIDFREFICALSITSRGSF
Q99JB8	421	IGLYPANYVECVGAXXXXXXX

Q60597	487	SDDPEAVMYVCKVAAEWRNTF
Q9EPB5	31	WGSQKNPPVLCLHGWLDNANS
P52825	512	IRPASIFTKRCSEAFVREPSK
P13707	7	XXXXMAGKKVCIVGSGNWGSA
P31749	310	IKDGATMKTFCGTPEYLAPEV
Q5SSW2	1343	GRDKFSPRRFCLFKGIFRNFD
Q8VBT1	652	SEGDSAVVPGCESREQPPPEV
P54071	154	GGTVFREPIICKNIPRLVPGW
P46459	91	SLYTFDKAKQCIGTMTIEIDF
Q80UM7	136	PGTPPKLRHTCEQGDGVGPYG
Q99832	364	GGERYNFFTGCPKAKTCTFIL
P70670	1820	ANSNSASSPKCPDPSSKKDTK
P12004	162	SHIGDAVVISCAKDGVKFSAS
P04117	118	KRDGDKLVVECVMKGVTSTRV
O70468	248	EVSTKDKFDSCNFNLTVHEAI
Q32MW3	299	EDTKLKSLDICHPQERNVFNR
P98170	300	YALGEGDKVKCFHCGGGLTDW
P62879	204	PDGRTFVSGACDASIKLWDVR
P86048	105	HVIRINKMLSCAGADRLQTGM
P10768	176	YKSVSAFAPICNPVLCPWGKK
P03995	291	DYRRQLQALTCDLESLRGTNE
Q9JKS4	653	LFHMEDGEPYCEKDYINLFST
Q99MN9	271	RAFDNDVDALCNLREFFNFLP
P41216	510	YLASKGEGEVCVKGANVFKGY
P21817	314	SKAHTKATSFCFRISKEKLDV
P15121	299	LLSYNRNWRVCALLSCTSHKD
Q9EQ20	86	KAEMDAAVESCKRAFPAWADT
Q8BK64	56	AVRVENEEGKCEVTEVNKLDG
Q76MZ3	174	AELRQYFRNLCSDDTPMVRRA
Q9JHU4	4568	VTGLKLQGATCSNNKLSLSNA
Q8BKT7	59	GRDYELYKYTCQELQRLMAEI
Q9CXT8	389	HVVQNEWKRLCTDVTESEVAR
P62320	20	LHEAEGHIVTCETNTGEVYRG
Q9QY76	121	PEDLMDSKLRCVFELPAENAK
P21980	371	PQEKSEGTYCCGPVPVRAIKE
Q9BXJ9	721	CMIRLFNTAVCESKDLSDTVR
Q9DCS9	125	QREGENYQQNCAKELEQFTKV
O75369	660	AYGPGLEKSGCIVNNLAEFTV
A2ASS6	33667	TATNTAGSTSCQAHLQVERLR
Q61768	65	STSQEQVYNDCAKKIVKDVLE
Q9HC38	221	AAAFGRIAFSCPQKELPDLED
Q8K2Y7	89	EKVKSGASWTCQQLRNKSNED
O43707	793	GALGPEEFKACLISLGYDVEN
P97823	144	QKLAGVTALSCWLPLRASFSQ
P09936	152	AHDAVAQEGQCRVDDKVNFHF
P50171	39	LAAEGAAVAACDLDGAAAQDT
P24270	232	LVNADGEAVYCKFHYKTDQGI
P21817	252	RLVYYEGGAVCTHARSLWRLE
Q99NB1	144	ITYRELLETTCRLANTLKRHG
Q8VC74	130	AKRLNQEVAVCARQFGWSKRV
P05063	290	ASLNLNAINRCPLPRPWALTF
Q9Z1Q9	681	PLLRPQWYVRCGEMAQAASAA

Q8R127	77	SLSSEVGVIICDISNPASLDE
Q03265	294	PLQYLAPYSGCSMGEYFRDNG
P21980	336	DKSEMIWNFHCWVESWMTRPD
Q9Z1Q9	380	RGETTLWNPGCDHAGIATQVV
Q9ESD7	834	VLFSRRGPSYCGRNCGKLQTI
P00558	108	DCVGPEVEKACANPAAGSVIL
O70251	50	AVSGPPPADLCHALRWYNHIK
P24270	425	QRSALEHSVQCAVDVKRFNSA
Q15149	3336	PATYGELQQRCRPDQLTGLSL
Q924M7	309	TPKFIDVPTLCEMLNYTPSPS
Q15185	40	NFEKSKLTFSCLGGSDNFKHL
P04843	477	PAAEARMKVACITEQVLTLVN
Q13868	68	GSVERVNKLICVKALKTRYIG
P50991	410	EAERSIHDALCVIRCLVKKRA
Q791T5	359	YSPVFKSWIHCWKYLSVQGQL
O55222	428	SPHVCKLMKICMNEDPAKRPK
P50990	430	AKQITSYGETCPGLEQYAIKK
Q9DCM0	34	LLRQMFEPKSCTYTYLLGDRE
Q99714	214	PLLTSLPEKVCNFLASQVPFP
P50396	317	PIKNTNDANSCQIIIPQNQVN
P19123	35	DIFVLGAEDGCISTKELGKVM
Q1XH17	456	PGPVYPIFDVCWHDKGKNAQP
Q5U3K5	501	AGHPRVAPQQCSEPETKWSST
Q01320	391	TLQAKSFGSTCQLSEKFIKAA
O08553	248	RSITIANQTNCPLYVTKVMSK
P10809	442	VEEGIVLGGGCALLRCIPALD
Q3ULD5	267	AEDLGGADLHCRKSGVTDHYA
P40124	29	EAVSHTSDMHCGYGDSPSKGA
P47934	449	LAYYRIYGQACATYESASLRM
Q9CY27	18	IRDAKTREKLCFLDKVEPQAT
P62717	64	MKKSSGEIVYCGQVFEKSPLR
P19246	263	AQAEARDALKCDVTSALREIR
Q9JJL8	425	RYGEVTSASNCTDFQSRRLYI
Q9WTI7	802	RGFILRHSPRCPENAFFLDHV
P30048	229	FQYVETHGEVCPANWTPDSPT
Q99873	262	VEDLTFTSPFCLQVKRNDYVH
P98170	90	VGRHRKVSPNCRFINGFYLEN
P10768	181	AFAPICNPVLCPWGKKAFSGY
Q6PB66	483	QETYINYVFPCFDSAQSVRAA
P26443	327	MRYLHRFGAKCVGVGESDGSI
P15105	359	YAVTEAIVRTCLLNETGDEPF
Q8BGK2	313	ESGATGTIAGCLFGLLHGLAT
Q8K3K7	248	DADVTKLVDTCYQSMRATFLQ
Q9Y3B4	74	YEDIFDAKNACDHLSGFNVCN
Q8R2L5	91	LSQFISPFTGCIYGRHITGLC
Q8BFR6	44	LEHRSKDSHGCSEVNVVKERP
Q08752	275	RAKLQPIALSCVLNIGACKLK
P27348	94	EKVESELRSICTTVLELLDKY
Q62095	297	KFSYRSRVRPCVVYGGADTVQ
A3KMP2	24	LPLSTTSNEACKLFDATLTQY
Q06830	52	FFYPLDFTFVCPTEIIAFSDR
Q91Z53	57	QGVVGAHGLLCRLSDRVDKKL

P53702	39	CPMHKGQRKGCPVTAATSDLT
Q12904	161	DVSRLDLRIGCIITARKHPDA
P50990	148	KAHEILPNLVCCSAKNLRDID
P00505	187	QGYRYYDPKTCGFDFTGAVED
P14824	552	SLETRFMTVLCTRSYPHLRRV
Q8BU88	70	EPRRPAEIYHCRRQIKYSKDK
O55070	73	MEIKDSSNNICPMLMEKLNGN
Q9CXT8	419	LLQLDGSTPICEDIGRQMLCY
Q8TCG1	108	NLNSVLAGVVCRSSHTDSVFL
Q9R0Y5	25	GGPGSGKGTQCEKIVQKYGYT
P70349	38	PAKIIFEDDRCLAFHDISPQA
P62911	96	EVLLMCNKSYCAEIAHNVSSK
Q09666	1833	EAEVPDVDLECPDAKLKGPKF
Q9D710	186	IYADLSLKYNCSGLNFGKVDV
P62258	98	MVETELKLICCDILDVLDKHL
Q9CZ42	104	KVGADLTHVFCAREAAPVIKS
P38060	141	SELFTRKNANCSIEESFQRFA
Q9D967	138	IIDVGRLGVTCIHIRDGMSLQ
P62241	100	LVRTKTLVKNCIVLIDSTPYR
O95573	573	GDIGEFEPDGCLKIIDRKKDL
P19246	225	QKKAQALQEECGYLRRHHQEE
O70572	411	DRGSEPHLAYCLQQEGDRAXX
Q922B1	203	LTDECRTLQNCETGKAKITCG
P68036	86	HPNIDEKGQVCLPVISAENWK
O08528	834	TVVARRAAQLCGAGMAAVVDK
Q8BGY2	38	NGFVVLKGRPCKIVEMSTSKT
Q9DB27	144	IMAEGKQHALCVGVMKMSAED
P53026	164	TIKFQMKKVLCLAVAVGHVKM
Q14498	478	NSAQGNVYVKCPSIAAAIAAV
P60842	66	SAIQQRAILPCIKGYDVIAQA
O08528	909	KGAALITAVACRIREAGQRXX
Q91WJ8	144	PDSGGLPERSCMLTGTPESVQ
Q99497	53	PVQCSRDVVICPDASLEDAKK
P06745	404	LIHQGTKMIPCDFLIPVQTQH
P31943	122	FVRLRGLPFGCSKEEIVQFFS
Q8BTM8	2293	ISFEDRKDGSCGVAYVVQEPG
Q8VDN2	518	KGAPERILDRCSSILLHGKEQ
Q8R010	306	PTNVQRWLKSCENLAPFSTAL
O15400	28	ISSNIQKITQCSVEIQRTLNQ
Q8R0F8	132	WTLAKSFTSSCPVSAFVPKEK
Q7TPV4	896	ARRYCHEVGPCAEALHAQVER
Q9DC61	465	THSRKLPHELCTLIRNVKPED
O88441	246	VKNYSNLLAFCRRIEQHYFED
Q9DB29	173	ANACLQVARDCGTDVLDLWTL
P22087	99	EPHRHEGVFICRGKEDALVTK
Q8BGH2	457	PMGVQGGDRICDGVQFGAGIR
Q8K4F5	127	GLLPQLGLVPCVLVGHSMGGK
Q99JY0	459	DGGQYALVAACAAGGQGHAMI
Q99LC3	112	RPLDIEFSGSCSLEKFYDDPK
Q00839	562	LNTLLQRAPQCLGKFIEIAAR
Q9BUJ2	377	ALYPHVLVKNCAVEFNFGQRA
Q80XM9	95	ILTMLLMLKLCTEVRVANELN

P50171	192	ARELGRHGIRCNSVLPGFIAT
Q9CXA2	310	SSATGSVFTGCAVREAKCGDF
Q8K0Z7	231	EEEKNLFKFICDASSLHQVRK
O08573	325	FKVAVNGQHMCEYYHRLKNLQ
Q9DCX2	101	DQEEKEDVKSCAEFVSGSQLR
Q9CRA7	96	KIQAIDATDSCIMDIGLDHMV
P26196	324	AYVTERQKVHCLNTLFSRLQI
Q01320	996	KVFKLQSSLTCNSMVLFDHVG
P21980	285	GQCWVFAAVACTVLRCLGIPT
Q9CPZ8	31	KIIREKARERCSEQVEDFTRC
P98156	120	MRTCRINEISCGARSTQCIPV
P54071	418	SGAMTKDLAGCIHGLSNVKLN
Q9Z1Q5	223	AYAREEFASTCPDDEEIELAY
Q99MR8	450	RQSALSKLRYCLHQYNIVGLR
P08752	326	DTKEIYTHFTCATDTKNVQFV
P68040	240	LDGGDIINALCFSPNRYWLCA
Q8BKC5	972	TKENVNATENCISAVGKIMKF
Q02566	1413	EEAVEAVNAKCSSLEKTKHRL
Q9D6R2	222	LQKCREVAENCKDIKFNEMYL
P21817	1590	QSERKNPAPQCPPRLEMQMLM
Q5SW19	734	FSPGVRFPESCQDEVRDQKQL
Q501J6	168	PYLERGDGPICLVLAPTRELA
Q80X90	1868	IEGPSKAEISCIDNKDGTCTV
Q8BMS1	470	KEVESVTPEHCIFASNTSALP
Q6P8J7	238	FDKPVSPLLTCAGMARDWPDA
P05063	202	LPDGDHDLKRCQYVTEKVLAA
Q61699	642	RNDAKNAVEECVYEFRDKLCG
P12970	182	ALCRKMGVPYCIIKGKARLGH
Q9R0H0	559	RAVQAVLRNLCLLYSLYGISQ
Q8C5H8	321	EKQKSSGLNLCTGTGSKAWSF
Q99KC8	374	LGGTEILTPLCNIYKASSIPG
Q8BIG7	133	ALPEAGRVVTCEVDAEPPKLG
Q14315	1103	GGLGLTVEGPCEAKIECQDNG
P04083	343	GDYEKILVALCGGNXXXXXXX
P15105	209	PAQWEFQIGPCEGIRMGDHLW
P30153	390	VRLNIISNLDCVNEVIGIRQL
P97461	66	YAAKRFRKAQCPIVERLTNSM
P80315	252	EKAKIGLIQFCLSAPKTDMDN
Q9D172	50	GARVALVLSGCGVYDGTEIHE
P26599	251	LDGQNIYNACCTLRIDFSKLT
P61080	85	HPNINSNGSICLDILRSQWSP
P27546	595	QHKGQSTVPPCTASPEPVKAA
Q91VH6	58	PHAGYTYCGSCAAHAYKQVDP
P13010	339	EQMKYKSEGKCFSVLGFCKSS
P63330	269	IFSAPNYCYRCGNQAAIMELD
P35486	41	ANDATFEIKKCDLHRLEEGPP
P49915	449	PGPGLAIRVICAEEPYICKDF
Q8VE38	240	LVHEFPEKISCSFHVTKQTAQ
Q7TPV4	890	MHHLCRARRYCHEVGPCAEAL
P30101	244	KFIQENIFGICPHMTEDNKDL
Q8BTM8	1997	VVPPSGREEPCLLKRLRNGHV
Q9JIK5	585	IPEVDLVVQSCPPKDVESYIH

Q8BH59	435	PLPAEILAGGCAGGSQVIFTN
O35737	290	GSTFQSTTGHCVHMRGLPYRA
Q60597	604	TLDGQPRSMTCPSTGLEEDVL
Q8BW75	26	GMAAAKLLHDCGLSVVVLEAR
P07355	133	TDEDSLIEIICSRTNQELQEI
P24270	460	VLNEEERKRLCENIAGHLKDA
Q99JR6	133	SAALPELKLLCGADVLKTFQT
O70468	715	EEWVFDKKLLCETEGRVRVET
Q8R1G2	198	STLTQKLKEHCIVNYQVKTFS
Q91VI7	404	SLRELDLSNNCMGGPGVLQLL
Q15149	950	MRGRLPLLAVCDYKQVEVTVH
Q91WS0	83	RCWRSKKFPFCDGAHIKHNEE
Q00610	918	YCEKRDPHLACVAYERGQCDL
P06151	293	INEDVFLSVPCILGQNGISDV
Q8QZR5	254	NPTGQVQTRECIEAVIRFAFE
Q8R016	164	VEKYGVVPKKCFPESHTTEAT
Q8CC88	1415	RTNATPRHNNCVTLTHTNQVV
P10620	50	TRKVFANPEDCVAFGKGENAK
Q8BFQ4	287	LDGKHTGPITCLQFNPKFMTF
P56546	44	PLVALLDGRDCTVEMPILKDL
P80313	370	FFTGCPKAKTCTIILRGGAEQ
Q9QUM9	47	LTSVAVRGKDCAVIVTQKKVP

Negative dataset $\mathbb{S}_L^-$ contains 2300 non-SNO sites and peptide fragments

Uniprot ID	Site	Sequence
Q8BMS1	97	SAVLISSKPGCFVAGADINML
Q9D892	154	RGSRDFGWDPCFQPDGYEQTY
B1AR13	70	ELVAGKTYRWCVCGRSKNQPF
Q497Q6	228	VPMSYIESEFCKKSRXXXXXX
Q8BMS4	238	HVSHLEMFIQCCYQVLKPGGS
Q64702	422	DENQHSSNHHCLGKTPFPFAD
Q9D0K2	28	RSSRGALHKGCVCYFSVSTRH
Q9Y272	24	DSELSIPAKNCYRMVILGSSK
Q9CZC8	186	AAERITEGVRCICNHLSLATK
A8C756	535	TWVFPVLSVLCGGNLDQRSYV
Q9WU78	691	TEILVRFQNKCSDIVFARKTE
Q99MK8	208	GRGGFGEVYGCRKADTGKMYA
P19838	703	VEHDNISLAGCLLLEGDAHVD
O08715	444	NPRGDDNFVACMANNSQSVLS
A2ASS6	30880	WTKGDKELDLCEKISLQYTGK
P54886	183	YEAMFTQYSICAAQILVTNLD
P12382	518	VEARGRYEELCIVMCVIPATI
Q6PDN3	669	FEQKGGWHSLCIQEVFPEDTG
P04350	201	QLVENTDETYCIDNEALYDIC
P70398	1340	QVAQEQFFLMCTRCCMGHRPL
Q15149	850	QWSWMLQLCCCIEAHLKENAA
O55229	53	RDAQRRAYQWCREYLGGAWRR
P78347	123	RKTVEDYFCFCYGKALGKSTV
P78527	2248	IKTLVECWKDCLSIPYRLIFE
Q64467	29	DRCPCPCPCPCPCPCPVIRPP
O00468	488	TCAVKNGQAACECLQACSSLY

P54310	418	DLLGSTGERICLAGDSAGGNL
P70388	1296	FYRVKKNMDQCSEIVKCSISS
Q9JKS4	606	RCYEQFFAPICAKCNTKIMGE
O15355	495	APDTSGDGTGCDNMTCIIICF
A2ASS6	22724	TYKVTGLTEGCEYFFRVMAEN
P98156	84	ESDFVCKNGQCVPNRWQCDGD
Q9R1V6	692	AGTVCSGNGVCSNELKCVCNR
Q6P3A8	28	GAERRRCGLRCAALVQGFLQP
Q8VDQ1	180	LAGQIGHLLGCSRVVGICGTQ
Q6P2B1	248	SSNLHEAASDCVCSALYAIEN
A2ASS6	6333	IKAGDSARLECKITGSPEIQV
P25205	123	PRTLTSCFLSCVVCVEGIVTK
Q8BFP9	17	LRGGTSVRPLCAVPCASRSLA
Q8WX93	1086	MAANPQGRISCTGRLMVQAVN
Q14498	303	YGFITFSDSECAKKALEQLNG
Q9P2E9	1216	EKHMAAASAECQNYAKEVAGL
Q9EPL8	567	DLTNVIQKMICEYSEEVTPIA
Q9JHU4	790	IESVRTYERTCEKVEERNTIS
O70433	224	CDLYAKKCAGCTNPISGLGGT
Q810L3	306	KMEETLTCIICQDLLHDCVSL
Q3UH68	1019	FHIQCFRCGICKGQLGDAVSG
Q9D1G3	90	LSGHVLFAKLCTMVAPQLRSW
Q9DC61	87	VASQNKFGQFCTVGILINSGS
P62702	181	DFIKFDTGNLCMVTGGANLGR
Q5VYK3	1638	NVKYKIVAISCAADILKATKE
A8C756	1722	ELQGTLSLWRCVLTLLQSEEQ
Q8K411	313	WDKPREFHITCGPDSLATETA
Q6P2B1	527	SAAAKAIHNICSVCRDHMAQH
O70433	101	DCYSNEYSSKCQECKKTIMPG
Q9CR68	236	ANAGDFGGYYCPCHGSHYDAS
P26039	1939	ALVTKAGALQCSPSDVYTKKE
P97823	173	DISVLQCHGDCDPLVPLMFGS
Q9D172	175	HGAKKPIGLCCIAPVLAAKVI
P53569	421	RKHPNMKGVVCGEIERLLFRS
Q8BP48	22	DGCSSEAKLQCPTCIKLGIQG
Q91YR7	339	RNLIMKGTEMCPKSEDVWLEA
P46459	334	IIIFDEIDAICKQRGSMAGST
Q99K43	209	DVVCEDESAFCLSLENIATLQ
A3KMP2	376	QLAKDVGLPLCQALLEAENGN
P19838	666	HLAMMSNSLPCLLLLVAAGAD
P13639	751	MEPIYLVEIQCPEQVVGGIYG
Q8BWM0	109	LQLTLYQYKTCPFCSKVRAFL
Q5VYK3	1722	CYRQELCKLMCERLKLSTWKV
Q9JKS4	550	ASSRTPLCGHCNNVIRGPFLV
Q8K4Q0	1047	CIAVADKDSICFWDWEKGEKL
Q8JZN7	506	DSSDPKTFVHCATIYKRYYMD
P54136	8	XXXMDVLVSECSARLLQQEEE
Q9Y696	35	AGSDGESIGNCPFSQRLFMIL
Q9JJL8	328	LPVRMVCASTCYRAETDTGKE
P98156	212	TSSCIPLSWVCDDDADCSDQS
Q8K298	1114	VDIRLWQPDACYKPVGKPXXX
P27695	99	PDILCLQETKCSENKLPAELQ

Q00987	2	XXXXXXXXXMCNTNMSVPTDG
Q8JZN7	488	SLLDTSLDTTCDVACLMFDSS
O00468	494	GQAACECLQACSSLYDPVCGS
P00533	215	ENCQKLTKIICAQQCSGRCRG
P26196	385	RVFHDFRNGLCRNLVCTDLFT
Q9Z2W0	269	PRLDNLHSCFCALQALIDSCA
O89103	464	GWELAPNGVFCSRGTVFSELP
Q3UHX9	270	YWGYTVRLASCLSAVFAEAPF
Q9CQ60	156	ILGVGPDGHTCSLFPDHPLLQ
P48725	1688	HVASIGCANPCADDELEQEGV
O60610	437	RPQYYKLIEECISQIVLHKNG
A2ASS6	19651	RINKDELIRQCAFRVPGLIEG
Q12879	460	MNVKKCCKGFCIDILKKLSRT
P52825	13	PRLLLRDWPRCPSLVLGAPSR
Q6PAR5	1167	LMAQLQETMRCVCRFDNRTCR
Q8R5A6	456	EGFSHFHLYVCAAFLVRWRRE
Q99JR6	163	QEIVEKFGLVCVSRSGHDPER
Q8C7H1	48	GALPNCFGHHCTKRVLLSDGF
Q9R0Q7	76	HKRTDRSILCCLRKGESGQSW
A2ASS6	27041	RKAYATITNNCTKNTFKIENL
O00468	447	DSDCWRQQAECRQQRAIPSKH
P38060	174	VRGYVSCALGCPYEGKVSPAK
Q9EST5	114	STLEPLKRLDCLKSLDLFGCE
Q9JI57	480	WSDMSSVSEDCGPGTSGEIAM
Q01320	215	LKPFSGEDYTCITFQPDLSKF
P08752	287	EKITQSSLTICFPEYTGANKY
P15105	42	WVDGTGEGLRCKTRTLDCEPK
Q9JJL8	324	AFRDLPVRMVCASTCYRAETD
P16546	1930	TVHKDRVNDVCTNGQDLIKKN
Q9BSK1	188	RTERGEKPHGCGECGKTFMRK
Q8R1B4	750	KAMKMGDWKTCHSFIINEKMN
Q99683	226	MITPHNKVYCCDSSFMKGLTE
Q60597	215	EIIRRLEMAYCQHIGVEFMFI
Q3UKJ7	383	VKIWNMKTTECSNTFKSLGST
A2ASS6	17608	EVFIDIGAQDCLVCKAGSQVK
Q80UJ7	117	MNNDFPPRAHCLVRWYGLREF
Q4VXU2	232	VMRDNSGHSRCFGFVNFEKHE
P12277	74	PGHPYIMTVGCVAGDEESYEV
P12277	146	SIRGFCLPPHCSRGERRAIEK
Q9ER72	138	QDGKKVTWYCCGPTVYDASHM
Q9BXJ9	20	ENALFKRILRCYEHKQYRNGL
P47712	220	KALYESGILDCATYVAGLSGS
P98170	227	WSEHRRHFPNCFFVLGRNLNI
O75694	102	ELVEQFGHMQCNCMMGVFPPI
Q9CQA3	198	CILCACCSTSCPSYWWNGDKY
Q8NBI5	382	TSLLCLGFALCASVPILPLQY
A2ASS6	6853	IDISQSGEYTCVVSNNAGQAS
Q99JY0	29	ALRSSIRPLSCSSQLHSAPAV
Q91W97	133	GTELFDYVADCLADFMKTKNL
Q3U1J4	604	TTFESSHYLLCALGDGALFYF
A2ASS6	14145	CTPEDIKTYTCDAKDFKTSCN
Q9EQ80	253	LLLHTGMGRLCTLDESVSLAI

Q8BIF0	11	MVARLTAFLVCLVFSLATLVQ
Q5VYK3	1768	EALAEILLETCKSITYSLENK
Q64727	85	PPAFIKVENACTKLVQAAQML
Q61598	203	RTDDYLDQPCCETINRIKLYS
Q9Z2I0	71	YLCFKGEPLSCWTQRPECQGT
Q3TL44	5	XXXXXXMRWGCHLPRTSWGSG
P39053	708	FSELLANLYSCGDQNTLMEES
A2ASS6	1868	VLEGETARFRCRVTGYPQPKV
Q8R2L5	137	DPAYLKDPRVCNIRYREXXXX
Q8BK08	116	SLPAGVLSLACCTLYGISWQF
O70145	513	GIFPKAFVEGCAAKNLEGIPR
Q9Z1W9	111	VAIKRINLEKCQTSMDELLKE
A2ASS6	28120	KKAWVSVTNNCNRLSYKVTNL
Q9JHU4	3710	TVTRSSLQSQCLNEVLKAERP
O95372	147	HPLAGIVALSCWLPLHRAFPQ
Q9D051	169	SQCFAAWYGHCPGLKVVSPWN
Q791V5	268	YSPIYTSWIDCWCMLQKAGNM
Q86VP6	206	IIALGHLVMSCGNIVFVDLIE
Q9D8B4	115	THSYGTAAMGCVYMGTAAALF
Q9DAS9	69	SENPFKDKKTCIILXXXXXXX
A8C756	1859	GWQSPHSQKLCHLQRIASEQS
Q9UHG3	242	NAFVGAVSLSCSDSGLWAVEG
Q99MK8	154	DLFQPYIEEICQNLRGDVFQK
P02463	39	GDCGGSGCGKCDCHGVKGQKG
Q8R349	72	SRKLDKLYEACRYLAARCHYA
Q13144	95	ATGVQETFVFCCWKAAQIKEH
P78527	2363	QNTMEDKFIVCLNKVTKSFPP
Q62407	1682	RRGLVIVTELCTEELLERMAR
P42574	220	KDGSWFIQSLCAMLKQYADKL
P14780	674	VFQYREKAYFCQDRFYWRVSS
Q91YE3	279	INGRTKAMVACYPGNGTGYVR
Q9CZC8	235	VFSPADDRLDCCAGQDSLEKQ
Q9D710	127	LTLCIVFLMTCKPPLYMGPEY
P06213	286	QDWRCVNFSFCQDLHHKCKNS
Q923E4	494	RLGGEYAKLCCNPVKLSEITE
Q3UZY0	300	HRATVLQIHFCDWQWAWEWRQ
P32020	288	YDMSKEAARRCYEKSGLTPND
A8C756	1752	VTTAMSQGNTCQSTEFAFCQV
Q99JW4	196	DKMGVPICGACRRPIEGRVVN
Q9D517	109	EIDFLCGWTMCERFGVLGSSK
P14618	317	FLAQKMMIGRCNRAGKPVICA
O70572	358	EEAREVAIILCIPSVGLVLVA
Q5SSW2	201	EMLEEWRPLMCPFDVTMQKAI
O95372	213	KTYPGVMHSSCPQEMAAVKEF
A2ASS6	3535	VAKLSVTVTGCPKPKIQWFFN
P84095	6	XXXXXMQSIKCVVVGDGAVGK
Q8CHP8	297	EDVKSNQESDCMFKKKMVPDF
Q06587	87	LRSGNKECPTCRKKLVSKRSL
A6H611	376	ERYNIEPSLYCPFLSLGACME
Q91YT0	385	KHESCGQCTPCREGVDWMNKV
P23368	190	PVGKLCLYTACAGIRPDRCLP
P25205	126	LTSCFLSCVVCVEGIVTKCSL

Q14315	535	VREAGDGVFECEYYPVVPGKY
P48725	758	LEEQLQKKESCHREMLTQELE
O60502	863	DPSVAKSMMACLLSSLKANGS
O00468	1597	DEKSPCQPNPCHGAAPCRVLP
Q9QUR6	410	SFLSPGVIYHCDLTKEELEPM
Q9CZW5	217	KECLEDVTAVCILEGFQNEQS
Q16665	255	RHSLDMKFSYCDERITELMGY
Q9CXJ4	91	PAVLWQHPRLCLIALCEAKES
Q9QYJ0	202	EGEVINEKDRCKKCEGKKVIK
Q9ET80	402	DQAALAARQECDIARAVAREL
A2ASS6	33564	VARNKHGEDSCKAKLTVTLHP
Q6NXE6	206	CSGIRCVRHACLKHEQNRQDL
Q8TAQ2	486	NPQEYLTSTACRRNLAGDVCA
Q64462	330	TTSGISWFLYCMALYPMHQQR
Q9D1G3	382	FVVTTLWLGPCDIVYLWSVLN
P70398	471	SPEQLDHLFDCFKASWTNASK
Q9DCD0	171	AAKVGTGEPCCDWVGDEGAGH
Q6A0A9	1097	HLNALSTDSACRREAALEAAV
P11142	17	VGIDLGTTYSCVGVFQHGKVE
P97822	27	EEVTELVLDNCLCVNGEIEGL
Q99798	126	SKVAVPSTIHCDHLIEAQVGG
Q9D883	155	SPVTDFREACCRQYEMGECTR
P28650	339	WGVTTGRKRRCGWLDLMILRY
A2ASS6	4698	VKVDDSGTYSCEATNDVGSDS
Q12879	1412	RSSLRSTASYCSRDSRGHNDV
Q62407	1057	KLSTAKDELTCSARLTVRPSL
Q9BQG0	623	SCDLLGDIQTCIRKSLGEKPR
P14873	143	RHKLLVLTGQCFENTGELILQ
P61978	205	IGGKPDRVVECIKIILDLISE
P62753	100	GERKRKSVRGCIVDANLSVLN
Q8K0S0	250	APKGSLGDRFCRDRLPLLDIA
Q9H9Q2	178	NNIVKTLHEWCDGCEAVLLGI
Q9JMA1	122	NTCYMNATVQCIRSVPELKDA
Q62407	1661	ARLLARLQHGCVLYFHEAFER
O60610	314	SGTTIALKVGCLQLINALITP
P21817	844	RGPHLVGPSRCLSHTDFVPCP
Q9CQN1	657	NPRHTLIKKLCQLRESEPELA
Q14139	202	PENLLPFAVQCRNLTVSNTRT
P68404	409	KPPFLTQLHSCFQTMDRLYFV
O00429	300	NRLLMHHIRDCLPELKTRINV
Q64277	150	ENASGLDYQSCPTSEDCENNA
Q9ER60	145	MFIMITILTNCVFMTMSNPPS
P09382	3	XXXXXXXXMACGLVASNLNLK
Q9JLV5	298	LKNGKTEDLACMYKLFSRVPN
Q62312	486	ALVLWEMTSRCNAVGEVKDYE
Q99KN2	142	WEVDEEDEYECVSVLSSHTQD
Q9CXT8	342	NLSSKLAQLTCHGNLCHSFQS
P47757	147	AGDGSKKIKGCWDSIHVVEVQ
P13639	388	DDEAAMGIKSCDPKGPLMMYI
O95219	318	LFYAEALRAVCRKHELMQYDL
Q00987	441	PLNAIEPCVICQGRPKNGCIV
Q14181	259	EPVTLLGQIGCDSNGKLNNKS

P70388	680	ITQLTDENQSCCPVCQRVFQT
P31327	761	KNVVSGKTSACFEPSLDYMVT
P21817	2158	SVEDTMSLLECLGQIRSLLIV
Q9BSK1	303	TGDKPYKCSDCGRTFYFKSDL
P43235	221	MYNPTGKAAKCRGYREIPEGN
Q810L3	476	SLPTAAPDYMCPLQGSHAICT
Q9H2U2	283	QCWKALLMKKCNGGAINCTNV
Q9JLJ2	220	QGGAATGQFLCHHREVAKISF
Q9D1C3	6	XXXXXMLSATCRRLAPALRRL
Q14444	257	DSTHNHQNGLCEEEEAASAPA
P26639	558	IKDAIGRYHQCATIQLDFQLP
P62918	90	EGIHTGQFVYCGKKAQLNIGN
Q06203	237	TEGWVVSSESCSFLSIGARYY
Q9Y570	347	GKFQMQVLPQCGHAVHEDAPD
P00533	1146	PEYLNTVQPTCVNSTFDSPAH
P21333	483	TVGQACNPSACRAVGRGLQPK
P07742	790	SLENREECLMCGSXXXXXXXX
Q9BUJ2	218	DTLVAIDTYNCDLHFKVARDR
O08715	392	EALPPKTYVSCLSSPLSGPTK
P53996	102	KREREQCCYNCGKPGHLARDC
P16675	235	NRLWTSLQTHCCAQNKCNFYD
Q9ESD7	1716	LRPSQLLHLFCQQHRIKAPVY
Q5UIP0	2450	QLFEMHEKLSCMANSVIKNLQ
Q8TEX9	95	LTALQRETEHCVSLSLAQLSA
Q5UIP0	880	KLDEVPKVYSCLNNKLEKLLG
P54822	113	AGIIHLGATSCYVGDNTDLII
Q8VEM8	252	KFVVPKPRSECTKAEQLVVTF
Q8VC74	323	TPYRDLSAAFCAKFLSRKRXX
Q9ER60	1562	ENPGTNIKGDCGNPSIGICFF
A2ASS6	21103	LLTVKAGTNVCLDATVFGKPM
O08715	461	SVLSVSSLGQCSDPVSTSGLE
Q99MR3	278	ASVFAVLFNGCTGIMAGANMS
Q9QYJ0	146	QLSKNVLCSACSGQGGKSGAV
Q920E5	117	DSSLTRRGQICWYQKPGIGLD
Q9EPL8	878	LFNGLKRAYACHAEHENDSDD
P50579	223	LNAGLAFPTGCSLNNCAAHYT
P15170	276	GANLKEQSDFCPWYIGLPFIP
Q9NPA8	50	CGWKDQLKAHCKEVIKEKGLE
Q9UL62	918	EVQGAAQSSECPLACSSSLHC
Q9DCN1	337	MQVIHPDGTKCLLGRQKRFPP
Q6IFX4	238	NHEEEVNSLQCQLGDRINIEV
P06213	328	GYTMNSSNLLCTPCLGPCPKV
O00468	1564	LPNPCHGGAPCQNLEAGRFHC
A2ASS6	9743	VSEHQSATFECEVSFDDAIVT
Q99K43	567	YPGSTPLQHNCSIKSVASTYS
P45880	8	XXXMATHGQTCARPMCIPPSY
P10768	56	PALYWLSGLTCTEQNFISKSG
P00533	260	VCRKFRDEATCKDTCPPLMLY
A2ASS6	27731	ICEAELRTTSCKVTKLLKGNE
O00468	607	GPCETCGDAVCAFGAVCSAGQ
Q04750	302	EKNTITNLSKCDFTQMSQYFK
Q3U186	11	MACGFRRSIACQLSRVLALPP

Q8BRK8	106	VSGGELFDYICKHGRVEEVEA
P13489	30	WAELLPLLQQCQVVRLDDCGL
P29218	201	RSVGTAAVNMCLVATGGADAY
Q9JJX8	412	NILTHTCPRGCSSXXXXXXXX
O00468	1112	RASCYNSALGCCSDGKTPSLD
P70388	143	CAEIDREMISCLGVSKSVLNN
Q62312	545	IQIVCETLTECWDHDPEARLT
Q6ZPY7	832	KRAVRGVREMCDVCETTLFNI
A2ASS6	33430	ITKFDDGTYRCKVVNDYGEDS
A2ASS6	7887	STIEDYAQYACLIENEAGQDI
Q5JTH9	839	SSPAKRPRLKCLLHIVRKLSA
Q92616	1984	SQKSDERQGVCIGLSEIMKST
P21817	3525	KKMLPIGLNMCAPTDQDLITL
Q8BGQ7	525	FVDEVVTGQECGVVLDKTCFY
Q9WUQ2	280	PHKRLRQPPPCYLTAWDSSTF
Q9JLZ3	169	LGGGLELALACDIRVAASSAK
O55070	244	TSCAYDRIVLCGQEIVNSVVP
Q05519	455	IETGSPKTKECSVEKGTGDSL
Q8BGK2	74	TAEALTTDYWCLDDLYREMVK
A2ASS6	22713	YSTVTTKCHKCTYKVTGLTEG
Q9JIK5	609	RTGRAGRTGVCICFYQNKEEY
Q5UIP0	1139	IVIPQDVTEDCGMAEHLEKSS
Q9Y277	36	MVKIDLKTKSCSGVEFSTSGH
Q99KC8	416	EVKLNSKKHRCFSFGIGQGAS
Q5M6W3	482	TDDLIQLITACPQIDLIRCLT
Q64277	76	AFKGVLDKDPCSVLPSDYDLF
P48725	586	QELTRDDLLPCSQCGQEPAMA
Q9WV60	107	ELQIMRKLDHCNIVRLRYFFY
Q99MN9	202	IPQISLIMGPCAGGAVYSPAL
O00468	650	GSACELREAACLQQTQIEEAR
Q3TBW2	64	IPPKPAINPRCLPPPPKPPKE
P20810	241	AIDALSSDFTCGSPTAAGKKT
Q9NYY3	462	TLGSCSSSSECLEDSTMGSVA
P00533	506	ENSCKATGQVCHALCSPEGCW
A8C756	228	DSSSPIWQSMCGLLSIFTKFL
A2ASS6	6864	VVSNNAGQASCTTRLFVKEPA
Q61151	314	RGLMKFWPKTCSQKEVMFLGE
P54310	163	LQEYVTLHKGCFYGRCLGFQF
O75153	1072	IQEYMHLALYCFASSQLSTAL
Q4G0N4	404	FSSKVCVRSRCWDACMVVDGG
Q69Z37	224	SNETFRFAAACMNSRTNGTIH
Q9D8B4	32	TYITTALGGICGIIGSAYRVS
P35235	367	TKEVERGKSKCVKYWPDEYAL
Q9JK42	212	KDAYDMAKLLCDKYYMASPDL
Q9DBC0	242	FYDGNPKYEKCTVVLRIAPTF
A2ASS6	4297	NLVPSDGKFKCLKEQNAYTLV
P53569	740	LFAKTILEGNCIQYSGDPLQD
Q08211	881	GCIFYVGDAICTIAAATCFPE
P63242	22	AGASATFPMQCSALRKNGFVV
Q9JI75	35	VAVEELSKQGCTVTVSDLYSM
Q8CC88	1230	SKEEGETYRMCKEFSHKNWVV
Q8JZN7	79	QEEIHKANVVCVVYDVSEETT

Q9JHK4	260	VSREEACLSVCFSRPLIVGSK
Q29RF7	904	SAIMKLAQEPCYHEIITPEQF
P21817	3014	PLINQYFTNHCLYFLSTPAKV
P35235	490	IDIIREKGVDCDIDVPKTIQM
Q92879	137	MFSSFGQIEECRILRGPDGLS
P54577	519	NFMTKLGSISCKSLKGGNISX
P58404	17	AAAVASAASSCRPLGSGTAPN
Q92616	932	HVTLRLLKPECVLDKSWCQEE
Q8BKC5	915	YFISPMLQYVCDNSPEVRQAA
O55091	235	CEDKQTFLQDCEDDGETAAGG
P30681	106	KRPPSAFFLFCSENRPKIKIE
P53618	235	LVIVELIYKVCHANPSERARF
Q924M7	251	LHQQHPGDIGCFAIYFLNLLT
O75694	637	IQPPAMSTPVCALGNPATQAT
Q8R1S0	132	AFRRMQVWDSCSEALIMFDRD
O70572	261	CCETLKTTTGCDPHSDKPFSD
O00468	2016	PKAYGTGFVGCLRDVVVGRHP
P02730	885	PLIFRNVELQCLDADDAKATF
P97821	290	PILSPQEVVSCSPYAQGCDGG
O75694	356	AVIENSESLDCQLLAVTHAGV
Q9GZT4	128	IQAYGASIVYCEPSDESRENV
A2ASS6	8625	KYQTTLTDNTCALTVNMLEEA
A2ASS6	15721	LDPDTDKWVRCNKMPVKDTTY
O60443	408	DSAAALLGTCCKLQIIPTLCH
P49327	2468	LGADYNLSQVCDGKVSVHVIE
Q9QZ73	90	KIGIDGIQQFCDDLALDPASI
Q9JHU4	3806	SQQYLPLSTACSSIYFTMESL
P13489	409	SLRELDLSNNCLGDAGILQLV
Q9BXJ9	817	EALYDGSLGDCKEAAEIYRAN
A8C756	591	HRGALGALMACLRTARAHGHL
Q8CG76	106	GLGLGLGSGDCTVKIATKANP
Q99MN9	21	AAGARLSVLNCGLGITTRGLC
P11172	86	YTALPLATVICSTNQIPMLIR
Q9UBF2	230	KSGLKSQFAYCMLIRIASRLL
Q1XH17	56	AADGTVACPCCQAPTRPQALS
O70325	64	VCLDKYRGFVCIVTNVASQUG
Q8BKC5	776	LSEIMHSFAKCIEVMGDGCLN
P49327	2024	DYFVVFSSVSCGRGNAGQSNY
P45376	299	LLSYNRNWRVCALMSCAKHKD
P07814	1480	GAPSMGAKSLCIPFKPLCELQ
A2ASS6	23493	RDKAGQRWVKCNKKALTDLRF
A2A432	321	ANLYKQLRQICEDHIKAQIHQ
Q9D1P4	176	YQGLQSLEEVCVYHSGVPIFH
P34932	270	IRALLRLSQECEKLKKLMSAN
P13489	323	CQLESLWVKSCSFTAACCSHF
Q3U5Q7	226	RDAARAVLEECTSFIPEARAV
Q8CC88	300	SQLLSFATTLCSQESSTLGLP
Q9D819	270	KAIVDALPPPCESACSLPTDV
Q01518	416	PTISINKTDGCHAYLSKNSLD
Q99LD8	176	VSGSSHLRGLCGMGGPRTVVA
Q5SSW2	500	PGVDPNDFSKCMITFQFIGTF
Q8K4Q0	1037	SVVKFHPFTPCIAVADKDSIC

Q9ER60	763	CGEWIETMWDCMEVAGQAMCL
Q9ER60	1724	TTLKRKQEEVCAIKIQRAYRR
Q9NVM4	368	RVRQMRPVCDCQAHLLWNRPR
A2ASS6	19362	SFSSVLTIKNCLRKDTGEYQL
Q9WTI7	860	LCMKNMVWKYCRSISPEWKQQ
O00468	172	TPVPPTPPDACRGMLCGFGAV
Q6PGB6	60	YFNDIAVGAVCCRVDHSQNQK
Q9R062	233	TYNPQTKSVNCDSQDPTVSHP
Q9NQC3	559	GLTPDLVQEACESELNEVTGT
Q9EQQ9	896	RILEFYSKLGCFEIAKMEGFP
O89103	308	ASRNPCSSNPCTGGGMCHSVP
Q8BGQ7	152	EAAGLEPDLECRQIWQNLGLD
P00533	526	WGPEPRDCVSCRNVSRGRECV
Q9ESD7	1631	SVSDQDNYIPCTLEPVFGKMF
O70468	1249	PCPYDGGVYVCRATNLQGEAQ
P08397	247	VLHDPETLLRCIAERAFLRHL
P98192	512	PGLRKEDVFSCFSFLRNVFSD
Q5UIP0	1692	RLHKRDSFDNCSLGESSKIGI
O00468	669	ARAGPCEQAECGSGGSGSGED
Q9BQG0	1096	LELLNVLFRTCKHEKLTLDLT
Q64436	352	PEGLLATVTVCLSLTAKRLAS
Q64436	365	LTAKRLASKNCVVKNLEAVET
P11172	255	RLMQKKETNLCLSADVSLARE
Q91WD7	740	VMNDNSQKALCRIESPLSRTE
Q99873	240	VDPKQLVTNACLIKEVDIYTV
A2ASS6	7980	VDHSDVGEYTCKAENSVGAVA
Q14166	572	ACAKPPPLGLCDYPSSRAMYA
Q9QYJ0	143	TKLQLSKNVLCSACSGQGGKS
O00468	729	GSDGVTYSTECELKKARCESQ
Q9DB43	35	EHCLVANHAKCIVQSYLQWLQ
Q12879	456	TNEGMNVKKCCKGFCIDILKK
P28474	211	GGVGLAVIMGCKVAGASRIIG
Q7TNG5	605	GKVHLFSYPCCQPRALSHKYG
Q99KN2	86	LASASFDATTCIWKKNQDDFE
Q9D1I5	126	KNKAGGMHHVCIEVDNISAAV
Q9D1P4	10	XMALLCYNRGCGQRFDPEANS
Q6P1X6	66	DDSKMKNFITCFKDPQFLVTF
P32921	309	KIFRDRTDIQCLIPCAIDQDP
P21333	2107	LEDGTCRVTYCPTEPGNYIIN
Q92616	1362	SQQVQESVASCLPPLVPAIKE
A8C756	1033	AEVKGKEEKACDVTAQMVLAC
Q7Z6M4	142	ILLGLNPEPVCVVLKKSPQLL
Q00610	436	QLNKYESLELCRPVLQQGRKQ
Q8C7B8	540	GCLCRALLEACRLEEEPHSLF
Q9ES28	77	LSNIREFLRACGASLRLETFD
Q9ET26	45	EVFEKPVQVPCGHVFCSACLQ
Q16658	260	KDELFALEQSCAQVVLQAANE
Q6P3A8	359	LNLEAPISRVCGYDTPFPHIF
Q9JHK4	186	SNYSSWHYRSCLLPQLHPQPD
Q8R5G7	633	NLLKNMAQLLCVETSEGEEPL
Q9CXA2	317	FTGCAVREAKCGDFKAVIVEV
Q9CPP0	79	LCLTEGATDECNVVEVVARDH

Q9DB29	137	RVILITPPPLCEAAWEKECVL
Q99K01	200	LCNQLGLPFPCLCRVPCNTMF
P48725	1668	LQVLYRPFLKCRMQLDQHQPH
Q91VH6	279	SFLNYAQSSQCRSWQDSSVSY
Q9DBF1	248	LEDNLLPGAICSLVCGGADIG
P30153	148	GDWFTSRTSACGLFSVCYPRV
Q9ER60	772	DCMEVAGQAMCLTVFLMVMVI
O00468	1128	TPSLDAEGSNCPATKVFQGVL
Q8JZN7	472	VNGQEKYLILCEVNADSLLDT
P21333	1353	SPFQVPVTEGCDPSRVRVHGP
O70546	960	DAFFPPLHQFCTNPNNPVTVI
Q99KQ4	401	TRDLLNCSFKCSYVVTNGLGV
O70325	29	LAAPGLAGTMCASRDDWRCAR
Q9WU65	529	PENGDNPVFSCLPLGFFIVSS
Q6NZJ6	940	NHDEESLECLCRLLTTIGKDL
Q91YE3	140	RSPGPERASLCPAGGGPGEAL
Q62234	694	EGKSYRFRVRCSNSAGVGEPS
Q91VH6	88	GPSHHVPLSRCALSSVDIYRT
Q8K1X1	83	NYHHNIGSPYCLRLASADVTG
Q8BRK8	413	HLGIRSQSKACDIMAEVYRAM
Q99KC8	5	XXXXXXMEHHCGLITSNKETV
Q8BKC5	986	VGKIMKFKPDCVNVEEVLPHW
Q9JLI6	129	EEGTRPHFITCTVEHDSIRLP
Q9BY44	132	SFIQKKMQNWCPSWSEDETLC
A2ASS6	5924	QCIIANEGGSCACSARVALKE
Q3TCJ1	62	QVIEIHNHQPCSQLFSFYDYA
Q7TPD0	594	LQKGSDTEAQCEVMQEIVDQV
P26639	656	FMADIDLDPGCTLNKKIRNAQ
P70388	102	EMVAVHRSMLCSQKNKKTEFK
Q62234	1142	DDGVISLNFECDQMTPKSEFV
Q5SSW2	1625	YDELKRDAKLCLSLMSQGLLY
P00533	595	YIDGPHCVKTCPAGVMGENNT
P00533	232	RCRGKSPSDCCHNQCAAGCTG
Q29RF7	589	PTCSCKQADICVREIARKLAN
Q8VDK1	199	TPAGKVGLAICYDMRFPELSL
Q06587	69	MTTKECLHRFCSDCIVTALRS
Q9BXP5	715	TQELGKDKWLCPLSGKKFKGP
P26639	174	AMERVYGGCLCYGPPIENGFY
Q9CXY6	311	VMTLEQQDMVCYTAQTLVRIL
Q99683	1351	LYYVTRDDLKCLRLRGGMLCT
Q64702	740	MDHANEGHRICLSLESVISEE
Q6NXE6	17	SRYSSGASIGCTPTSTQAKMV
P97447	209	RFTAVEDQYYCVDCYKNFVAK
Q6IFX4	36	RTNSSSNKSCCHDGQSTGCAL
Q9CPY7	145	LELPSVEVDPCGDAQAAAEGA
P62736	2	XXXXXXXXXMCEEEDSTALVC
Q8CGC7	5	XXXXXXMAALCLTVNAGNPPL
P16546	1314	PESAEDLKEKCTELNQAWTSL
Q9D1G3	477	TTLAVLFVTYCGVQLVKERER
P19096	1354	KGHALGETLACLPSEVQPAPS
Q96EK6	179	GYTVSEENYMCRRFLKXXXXX
Q9DB26	3	XXXXXXXXMACLSPSQLKKFQ

P97390	359	EVSEVEQELACQNDHSSALQN
P50462	40	FHKTCFHCMACRKALDSTTVA
Q9DBC0	462	LTGADFTNTFCVLSSFPADLS
P18206	313	LDEAGKVGELCAGKERREILG
Q8BIJ6	414	VASQHSLPMDCLVDEGGMFTD
Q6NSR8	311	GFKDNLHAVFCLAENAVGPNA
Q11011	266	FVETRSKDGVCVRVYTPVGKA
Q9BY44	80	LHSFDLLKAVCLEFSPKNTVL
Q3UK37	25	LPEAVAALSRCLPAGPSPEIF
Q9ESW4	370	AGTECLQASHCTLVLPEGTEG
P78527	3781	MNGILAQDSACSQRALQLRTY
Q9CZW5	478	FEEIIKKFPRCAEGYALYAQA
P00505	272	RHFIEQGINVCLCQSYAKNMG
Q99714	91	KFGRVDVAVNCAGIAVASKTY
Q9H2U2	302	NVQISDSPFRCTQEEARSLVE
Q61699	659	KLCGPYEKFICEQEHEKFLRL
Q8R1F1	194	QAVLQDCVRHCNNGIPENSKV
Q91W97	386	TIVSFRSANLCAAALATILTR
Q15149	1405	IERHGEKVEECQRFAKQYINA
P10518	122	FPSLLVACDVCLCPYTSHGHC
Q99683	225	YMITPHNKVYCCDSSFMKGLT
Q922B2	259	SPQLYKQMCICADFEKVFCIG
A2ASS6	22158	APSESTGTIICKDEYEAPTIV
P21817	3785	AEMVLQMISACKGETGAMVSS
Q8BUN5	31	GEQNGQEEKWCEKAVKSLVKK
Q99873	216	WENVYGFDMSCIKDVAIKEPL
Q92616	121	SAALLALTWTCLLVRIVFPSR
Q91YR7	698	LGNISAAQELCEEALRHYEDF
P30048	45	CGRTSLTNLLCSGSSQAKLFS
O00468	1832	SGHPCLNGASCVPREAAYVCL
O43143	599	SITAMLSVPQCFVRPTEAKKA
Q9UL62	248	KAEYEELSQQCKLFAKDLLDQ
Q6NZJ6	823	PNFSVAYANMCRCLMALKVPT
Q9H9A6	469	NKLSFISLELCVLQKLTFLDL
O89103	406	GCINTDGSFYCSCKEGYIVSG
Q99700	332	EEIMESILFKCSDFVVVQFKD
O14744	196	WMWWHNFRTLCDYSKRIAVAL
Q921S7	177	TEDIPKRETYCPLIVDSLIQL
P35557	230	CEVGMIVGTGCNACYMEEMQN
P78347	80	KDFQKDFVKYCVEEEEKAAEM
P17710	669	SFPCKQTSLDCGILITWTKGF
Q9Z1Z2	152	HTSGIKKALWCSDDKQILSAD
A2ASS6	24676	CDVSVGEWTMCTPPTGINKTN
P22102	1005	TVQLGENGKICWVKEEXXXXX
A2ASS6	4321	VKTEDEGEYVCEASNDSGKAK
Q99JB8	36	VQRVEDGHRLCGDLVSCFQER
Q8TCG1	58	NQILTSECLSCLVELLEDPNI
Q921M4	829	LKERVEELEHCCIQLSGETDT
Q9R190	106	SLPATHIRGKCSVTLLNETDI
Q6ZWY3	59	HAQTVVLCVGCSTVLCQPTGG
P08752	225	CFEGVTAIIFCVALSAYDLVL
Q6PDN3	43	PAFILPPRNLCVKEGATAKFE

Q3TXS7	806	KMPKVQYKSNCKPSTFAYPAP
P78527	1128	EKSLGTIQQCCDAIDHLCRII
Q61024	455	KHLLRETFEDCNLLPKEILWR
P27816	654	VLEKLGERKPCNSQPSELSSE
Q92879	119	KLFIGMISKKCTENDIRVMFS
Q99KN2	261	HTRTIYDVAWCQLTGALATAC
Q14315	805	GQGDVSIGIKCAPGVVGPAEA
O75153	333	TAPQAEHAMDCVRAEDAYTSR
Q8R086	318	HRLCDSEAHVCFEGLDSDPTG
P29474	707	GWAQAAFQAACETFCVGEDAK
O70546	1358	SNSRKTYIVHCQDCARKTSGN
Q86VP6	500	SSNLKIDALSCLYVILCNHSP
Q99KB8	35	LGQALLGLSLCHSDFRKNLTV
O00468	766	APLPPVAPLHCAQTPYGCCQD
Q9CPV4	206	AAAFGRIAFSCPQKELPDLED
Q8K2B3	190	FGKGGQAHRCCCVADRTGHSL
Q99JW4	193	PCHDKMGVPICGACRRPIEGR
Q5VYK3	1459	EKEEPIYKTSCALTIHAIGRY
A2ASS6	6384	LGTEDSGDFICEAQNPAGSTS
Q99JW4	275	SALNKAWCVSCFACSTCNTKL
Q8R0N6	243	VVANSGFDVLCHALESYTAIP
Q9Z2W0	239	SPDSIMEMELCLADTQPAVLG
P22314	748	GAPFWSGPKRCPHPLTFDVNN
P28651	78	DVRLSPNYVVCRDCEVTNDGH
P55072	209	NEVGYDDIGGCRKQLAQIKEM
P22102	559	EAVVAGIAKACGKAGCALLGG
Q9R1V6	347	RSGAAYIGGICSLLRGGGVNE
A2ASS6	30780	SEVGDGRWLKCNYTIVSDNFF
Q9CR76	27	WRRPLQGLWCCSGQGDSKRWV
Q62407	57	FLRPLKNAAVCAGSDVRLRVV
P78527	2244	NLEIIKTLVECWKDCLSIPYR
Q9R190	261	TLVPQGGPVLCRDEMEEWSAS
Q5UIP0	895	LEKLLGEIIACLQFSYTGTYD
Q8BGC4	330	LELYARGDLVCEVDLGHLAPD
P00533	591	QCAHYIDGPHCVKTCPAGVMG
Q01320	1142	YLTKEKKDELCKQRNEKEQEL
Q8BUN5	338	PATVCKIPPGCNLKIFNNQEF
A2ASS6	30207	DSFSTLTVENCNRNDAGKYTL
Q9H0C8	242	ANLGDSRAILCRYNEESQKHA
P10768	28	VFEHDSVELNCKMKFAVYLPP
Q8CGC7	910	EAKVLFDRVACQGEVVRKLKA
O94979	787	PNIMQLRDRLCRAQGEPVAGH
A2ASS6	15416	SRIKGYIVEKCPRGSDKWVAC
P21817	345	PPEIKYGESLCFVQHVASGLW
O00468	320	FKKFDGPCDPCQGALPDPSRS
Q91YP0	135	CNLKGIPYRQCGKLIVAVEQE
P21333	2293	ISFEDRKDGSCGVAYVVQEPG
Q6ZPY7	1514	DFVSPEHVKHCFRLTQEFRHL
Q9WUM3	192	VSWNHNGSLFCSACKDKSVRI
Q9WV60	76	SFGVVYQAKLCDSGELVAIKK
P70168	436	DTTAWTVGRICELLPEAAIND
O75694	874	PLLYSTDDAICSKANELLQRS

Q8BGX2	155	DAQASLYQARCRYLQPRWVDF
Q14690	1473	PQAQKRGGRECRESGSEQERV
P62932	403	KSDLIKTTLQCALERELKGHV
Q8VE38	113	GVSVVGGFSICSSPQRLERDR
Q9Z2U0	70	RKICALDDNVCMAFAGLTADA
Q9CXJ4	465	TFQNVTFSYPCRPGFNVLKDF
P14780	373	SEGRGDGRLWCATTSNFDSDK
Q5VYK3	1420	TDRNSVIQKSCAFAMGHLVRT
P21333	2479	PSKVKMDCQECPEGYRVTYTP
Q9WVA4	38	QILIQWITTQCREDVGQPQPG
O00468	868	DCEQMTGLCSCKPGVAGPKCG
Q9DBC0	203	VLRSSIREFLCSEAMFHLGIP
O75153	912	AKNYFDFDLECETVDQAVETY
Q61768	302	GNCRTTIVICCSPSSYNESET
Q9JHU4	2637	ELLLKTFDHYCEYRRTPNGVV
Q9C0B1	346	QRCQLALQNVCDDVDNDDVSL
Q16658	19	AVQIQFGLINCGNKYLTAEAF
Q6P1X6	22	ALARSRGARACSGDGGVSYTQ
P00533	19	AALLALLAALCPASRALEEKK
P63318	20	DSEGGPRPLFCRKGALRQKVV
Q7TPR4	690	NYKPKIDQLECDHQLIQEALI
Q3TL44	735	HPLDEVNLASCQLDPAGLHTL
P21817	1191	IEIGDGFLPVCSLGPGQVGHL
Q99683	206	KEIICQKNTMCTGNYTFVPYM
Q8BW75	156	MTMKELLDKICWTKSTKQIAT
Q9BUJ2	331	DKFAENDVIGCFADFECGNDV
A8C756	457	LEWHIKGKYACLGCLVETLGI
Q8BUV3	154	LPGSKKGSQECFQFILPALPH
Q8WTY4	244	SCGEGKKRKACKNCTCGLAEE
Q9DAK9	70	SGELQRNGYDCECLGGGRISH
O60610	1027	MTLLHFLAELCENDYPDVLKF
Q99NB9	1204	HMSLGVYGFGCEDSLNHLLNY
Q8QZR5	405	QVFNEAPGIRCNPVQGAMYSF
Q5UIP0	596	IQLIFNNFLECGVSDERFFLS
Q64520	116	GVRSIKKTDLCPIYIFVQPPS
Q3TL44	199	PAFELLIPFSCEDLSSLGSTP
O76021	211	TVLNISKSGSCSAIRIGHVGM
Q9JHU4	3145	PTHREAIVNSCVFVHQTLHQA
P41216	464	QFYEGYGQTECTAGCCLSLPG
P80314	412	KDPRTVYGGGCSEMLMAHAVT
P50396	17	VIVLGTGLTECILSGIMSVNG
Q8CIB5	12	ALDGIRMPDGCYADGTWELSV
P56380	143	ATLQEGHQFLCSTPAXXXXXX
Q9ER72	175	RDYFQYDVFYCMNITDIDDKI
Q64737	769	EAWVIGSVVACPEDSPRVRVK
Q8C460	313	IGRPHSGIDDCKNIANIMKTL
P62829	125	SAITGPVAKECADLWPRIASN
P06213	239	TAEGLCCHSECLGNCSQPDDP
Q60770	48	DEFTTKLLSSCCKMTDLLEEG
Q6A0A9	147	WFLPPVCMAHCIRLALIRFHV
P58281	11	MWRAGRAAVACEVCQSLVKHS
Q00987	475	AKKLKKRNKPCPVCRQPIQMI

Q921G7	588	LQINAQNCVHCKTCDIKDPSQ
P12268	339	SICITQEVLACGRPQATAVYK
P58404	271	VLEQIPFLQNCEDEDSDEDDE
P06213	186	KDDNEECGDICPGTAKGKTNC
Q9JHK4	487	ALPPALAALRCLEVLQASDNV
O00468	750	RGLYVAAQGACRGPTFAPLPP
P62827	120	RVCENIPIVLCGNKVDIKDRK
Q91YP0	259	KEIRCRYVVTCAGLYSDRISE
Q91WD7	538	IPEALNKELHCHHLHLQNKEL
Q9Z1Z0	499	QTRVGLLMLLCTWLSNCPIAV
Q9CZW5	547	ISKAIEIDNKCDFAYETMGTI
Q9D7N3	25	WFTPAVRASLCQRPGYWTASA
Q9D2G2	7	XXXXMLSRSRCVSRAFSRSLS
Q3UMB9	264	QLLDGMIFQACIEQQFDSLNG
Q9ER72	110	QWSPPAGTEPCRLRLYNSLTR
Q8WX93	1058	YTIQRDLDGTCSLHTTASTLD
Q29RF7	508	AVKALNEMWKCQNMLRSHVRE
Q04206	105	CRDGFYEAELCPDRCIHSFQN
O14920	115	RKYLNQFENCCGLREGAILTL
P63254	28	TSLGKDWHRPCLKCEKCGKTL
Q7TNG8	251	PAPEATVAATCAFPSVQAAVD
O00220	204	TFRNDNSAEMCRKCSRGCPRG
Q9JI75	122	ILKGWMDRVLCRGFAFDIPGF
P62918	115	GTMPEGTIVCCLEEKPGDRGK
O00468	1576	NLEAGRFHCQCPPGRVGPTCA
Q99798	284	SISCTGMATICNMGAEIGATT
P55072	174	KVVETDPSPYCIVAPDTVIHC
Q9WUQ2	357	TDVTFLPEKGCGPKLLGPHET
P34932	167	MDATQIAGLNCLRLMNETTAV
Q9CXJ4	670	SIIVMANGQVCEAGTHEELLK
Q9UHG3	462	DRLYYLNGIECAASAMEMSAI
Q8BGD5	73	LFLFSTIQLACLLQLDPSLGL
Q8WTY4	235	PDPASLKAPSCGEGKKRKACK
O89103	397	AAANSPCAQGCINTDGSFYCS
Q64436	487	MGYRDRFPKVCEIPFNSTNKF
P45377	50	YRHIDCAYAYCNENEVGEAIQ
P13439	394	KAAVGMAEEHCEFVIGFISGS
Q8QZT1	25	LLRGLLQEVRCLERSYASKPT
Q05586	22	LFSCSVARAACDPKIVNIGAV
O14983	561	EWGTGRDTLRCLALATRDTPP
O00468	1558	CGDHPCLPNPCHGGAPCQNLE
P68366	20	GQAGVQMGNACWELYCLEHGI
P70168	826	ACAAGLIGDLCTAFGKDVLKL
P62983	121	NGKISRLRRECPSDECGAGVF
Q99K01	443	GEQLKQLVPQCGLTVIDLEVD
P83731	36	GKVFQFLNAKCESAFLSKRNP
Q08211	888	DAICTIAAATCFPEPFINEGK
Q9WTI7	679	EAFLQRYKSLCPETWPMWAGR
O70433	89	PFAAKEEQLLCTDCYSNEYSS
A8C756	71	LLEKVDKNGVCDPAIQSCLDI
Q06587	332	GASDTGGPDGCGGEGGGAGGG
P47857	550	IGADTALNTICTTCDRIKQSA

P50991	252	EKAKIGLIQFCLSAPKTDMDN
Q80ZW2	29	LDGWYLVRVPCAVLRARLLQP
Q9BXJ9	322	KFLRMNFSKGCPPVFNTLRSL
P22102	733	SEEEMARTFNCGVGAVLVVSK
Q92616	2501	VAVNVAPGRLCAGRYSSDVQE
Q8K4Q0	36	LPLAFMKKRHCEKIEGSKSLA
Q9UNM6	49	LQVLDFVQDPCFAQGDGLIKL
Q8BVZ1	284	TQELWGSWSPCLENGRSHSEV
P61922	197	RGFSKEELETCMVNQSPGCPD
P53041	442	EGYEVAHGGRCVTVFSAPNYC
Q9BXJ9	309	NFLSGEKFKECLDKFLRMNFS
P14131	127	RTLLVADPRRCESKKFGGPGA
Q3UMB9	659	HLESHELLLDCYDKEIMDILN
Q3TC72	235	TIADPHNLKICCRVNGEVVQS
Q13557	481	GSPTVPIKPPCIPNGKENFSG
Q8CFE6	228	LGYTSGLSLLCMIFFLIVVIC
Q08752	176	EVKGEKPAKLCVIAECGELKE
P35486	190	ACKYNGKDEVCLTLYGDGAAN
P58059	60	RRRQRESYETCRRIYNMEMAR
Q86VP6	592	DQEVKERAISCMGQIICNLGD
P19096	274	PSGEVQEQLICSLYQPAGLAP
Q5SSW2	1840	LVLTDLLVSPCYYAXXXXXXX
Q9JLI6	375	RTLLASVGASCHSNHEDRPSP
P22102	891	VLEEFSIDIVCLAGFMRILSG
P32067	231	ETRALEGKMGCLLKFSGDLDD
A2ASS6	18422	FLSDNLTNDSCKLTWFSPEDD
Q9JIX8	513	PLAQKSSLPECSTQKGVESER
Q8BWF0	330	KFRNAGQTCVCSNRFLVQRGI
Q6P2B1	908	FHKQVTSAEECKQVCWALRDF
O08715	700	LRSLDQQMYLCYSQPGIPTLP
Q99J09	186	SPHKDSVFLSCSEDSRILLWD
Q9D6M3	290	GPSAFLKGAYCRALVIAPLFG
P68036	17	LMKELEEIRKCGMKNFRNIQV
P62746	83	SYPDTDVILMCFSVDSPDSLE
Q99KN2	19	SRVPAHPDSRCWFLAWNPSGT
Q02257	90	ARAKRVREAMCPGVSGEDSSL
O75694	556	QACATCLILACSTAACDREVS
Q8CGY6	207	LAAVRTLSGMCSGHRARATAI
Q99L45	282	YIKEYVTCHTCRSPDTILQKD
Q9DBC0	82	LATPRPVPGACFSRARPAPLR
P14873	2138	PSGGKQQGRQCDETPPTSVSE
Q6NXE6	6	XXXXXMSERCCSRYSSGASIG
Q9D1G3	393	DIVYLWSVLNCFGLNFELWVQ
Q8C7B8	366	LLGTWDKLDVCPLEEGNYSLD
P40227	282	KKIIELKRKVCGDSDKGFVVI
Q9BSK1	118	SDAFGGYGRSCLHIKRDKTLT
Q62523	526	VALDKNFHMKCYKCEDCGKPL
O70546	900	YPSSAEVLKACRNLGKNGLSN
P54136	615	ATAFTEFYDSCYCVEKDRQTG
O95573	466	SATTQRFMNICFCCPVGQGYG
P42704	380	SVFGSFFLQHCVTMNTPVEKL
O89103	644	NQYSPTPGTDCXXXXXXXXXX

P22102	466	PLAKATSRSGCKVDLGGFAGL
A2ASS6	33458	KGVREVYDYYCRRTKKVKRRT
Q9DCZ1	205	GYPQLSAVIECADSAHGLKGH
Q99NB9	1244	LEGLRVAIGPCRMLQYCLQGL
Q8VDQ1	344	TGGNVGKQIVCISEDSSLXXX
Q08752	52	PKTAENFRALCTGEKGIGHTT
Q5UIP0	1718	LSEKTFQTLECQHKRSRRVRR
Q02566	539	MGIMSILEEECMFPKASDMTF
Q6ZQ73	1138	FIMLARLATLCPAPVLQRVDR
P98156	127	EISCGARSTQCIPVSWRCDGE
Q06203	503	GLECFEKSGHCTACLTGKYPV
Q9DCS1	120	AFLHKKRGGTCWALMRTLLVL
Q8BGQ7	711	VPVSELLDDPCGPAGSLTSVE
P00966	97	YLLGTSLARPCIARKQVEIAQ
A2ASS6	23241	PPSEPSDAITCRDDLEAPRIM
Q9D1G3	187	HGGSSFTVLRCTSFALESCAH
Q5UIP0	1865	PNENFKTVGPCLGDSKNVSQE
P51174	303	LLIAELAISACEFMFEETRNY
P07742	544	TIYYGALEASCELAKEYGPYE
O08739	140	YQRVTISGDYCAGITVEDYEQ
Q8K1X1	1070	GKLAEGVQLLCLIDKAADACR
Q9DCN1	287	WHSRYKFCPTCGSATKIEEGG
A2ASS6	14095	VEEEATAVLECEVSRENAKVK
P54729	269	LLDADRYFCECKELLDTVDNY
Q810L3	603	RITGNTVLCYCCGLRSFRELT
Q8VCT3	130	QALCVAFRQPCGAADRFELEL
Q04760	19	GGLTDEAALSCCSDADPSTKD
P62932	124	NIMKETPSEECLDTALALQDQ
Q09161	320	ERFVIEENLHCIIKSHWKERK
P19367	628	TWTKGFKATDCVGHDVVTLLR
Q921G7	501	DSDQLKPAKDCTPIEYPKPDG
A2ASS6	20504	LKVSDVTKTSCHVSWAPPEND
P85094	107	GRSQLQSVLLCGIETQACILN
Q9D1P4	5	XXXXXXMALLCYNRGCGQRFD
Q8K4Q0	196	MGSPSIFVYDCSNAGLIVKSF
O70546	1234	TVHWVQAIGWCNNIAWNVGPL
Q6ZQ73	374	SRPDLLPDFHCTLAPALIRRF
P07742	352	VETNQDWSLMCPNECPGLDEV
O08739	47	VMSLFTVPEDCPIGQKEAKER
P19253	190	QAEKNVEKKICKFTEVLKTNG
P07814	1487	KSLCIPFKPLCELQPGAKCVC
P31939	575	SAADKVVIEACDELGIILAHT
P13489	45	LDDCGLTEARCKDISSALRVN
Q9D6M3	246	GSAAAVAVNPCDVVKTRLQSL
Q01433	123	LDLRTSMDGKCKEIAEELFTR
Q7TNG8	113	GGVCAVQGGVCINLTHMDQIT
Q8CDN6	64	AVFLEVDVHQCQGTAATNNIS
P26039	2196	TAKAVAAGNSCRQEDVIATAN
Q8R3F5	206	GQRQSNFSFACLEAQEHCKSL
Q8VBZ3	454	REHRVAGIFPCPTFKDKSTYI
Q92616	36	RREILSELGKCVAGKDLPEGA
Q8K297	407	GRPLTKGELGCFLSHYNIWKE

A2ASS6	6705	KDTGGVLGTSCILECKVAGSS
Q9DB43	97	PRNTAPAGYQCPSCNGPIFPP
O00468	468	QGPCDQAPSPCLGVQCAFGAT
P50991	221	VKKLGGTIDDCELVEGLVLTQ
P70398	2390	YQKRAYQCIKCMVALFSSCPV
Q11011	390	SWIEYLCVDHCFPEYDIWTQF
Q9BY44	377	PDGEHILTATCAPRLRVNNGY
P07814	1448	DSGKIVQIPFCGEIDCEDWIK
A2ASS6	19587	PSPSSDPIKACRPIKPPGPPI
Q9CPZ8	77	YYNDPAFYEECKLEYLKEREE
P78527	90	ECREEILKFLCIFLEKMGQKI
O70468	905	GGLDGYSVEYCQEGCSEWTPA
P21980	211	PKFLKNAGRDCSRRSSPVYVG
P98156	205	AHEFQCSTSSCIPLSWVCDDD
Q8CI94	319	IRRFKSSRFGCRDPVRTCFET
Q8K1X1	363	TKTVRPFSMVCCPVNENAAAL
Q3TXS7	217	NLEKPDFINVCQCLIFLDDPQ
Q9R1V6	392	KRKLASGECKCEDTWSGCIMG
Q8K1X1	1135	KALLVLLSLGCFVSVAETLHS
Q9DCZ1	222	LKGHIISDGGCTCPGDVAKAF
Q9BXJ9	58	TLAMKGLTLNCLGKKEEAYEL
Q60759	375	EMVSMLKRNNCGKALDIARQA
Q91V76	187	RRTGELNFVSCMRQTLEEHYG
P11499	412	KVIRKNIVKKCLELFSELAED
Q9NYY3	83	IVDPTTGKRYCRGKVLGKGGF
P49327	2312	YRVAGYSYGACVAFEMCSQLQ
Q8R1S0	13	ARIGSMAGLLCVRWWSSAQLA
O09061	223	DVYTGDALRICIVTKEGIREE
A2ASS6	21629	RKSWSTVTTECSKTSFRVSNL
P00403	35	DHALMIIFLICFLVLYALFLT
Q8TCG1	280	YLTRYEHFSSCLHQVLGLLNG
Q8BIJ6	609	IWFDSGTSWSCVLQDTQQRAD
Q62523	465	YHPQCFTCVVCACPLEGTSFI
Q3TL44	713	LAGVRMTPLKCTVVASVLGSG
O70433	10	XMTERFDCHHCNESLYGKKYI
Q3UZY0	513	RQKMSQHRENCLAERMAILQA
Q8R5K2	631	IVTYDLLSVICHHGTASSGHY
P26039	1363	CTQQAPGQKECDNALRQLETV
O08529	498	TFEPHKDGDFCIRVFSEKKAD
Q32MW3	154	VTVLVDKIDMCKHSLSPEQDI
P17405	607	LSARADSPALCRHLMPDGSLP
P00492	66	EMGGHHIVALCVLKGGYKFFA
Q6PDY2	206	PPYDPEDGRDCHYYRVVEPIR
Q99N94	218	EPITRWGEYWCDVTVNGLDTV
Q96EP5	39	YFSQYGEVVDCVIMKDKTTNQ
P50462	147	GKPWHKTCFRCAICGKSLEST
Q9NYY3	347	GFTPDRLSSSCCHTVPDFHLS
P54310	396	EAPFPRALEECFFAYCWAVKH
P53811	191	LANTPDCPRMCAYKLVTIKFK
P23368	428	ALSNPTAQAECTAEEAYTLTE
Q99683	250	PNFELLLGPICLPLVDRFIQL
A2ASS6	16766	LEGLTYVFRVCAENAAGPGKF

P97494	152	CTITSFPRLGCPGFTLPEHRP
Q14139	450	AALLKLCQPFCKPRSSRLLTF
A2ASS6	20411	MRKLVVVRAGCPIRLFAIVRG
P08113	645	VVSQRLTESPCALVASQYGWS
Q8BG51	157	IMNQYTEIETCVECSAKNLKN
P97390	145	HLFSLNILGCCQGRNWDPAQL
Q6NZJ6	1516	VRARLLQKYLCDEQKELQALY
Q9NVM4	136	VTVGPEGDMPCRANILVTELF
Q9Y3C8	165	QKGVIQHKEKCNQXXXXXXXX
P26039	1392	QPINDMSYFGCLDSVMENSKV
Q8R143	117	LLGITVCCCYCCRRKKSRKPD
P17812	243	FCHVEPEQVICVHDVSSIYRV
P00492	23	DEPGYDLDLFCIPNHYAEDLE
Q00610	39	TLTMESDKFICIREKVGEQAQ
Q6ZQ73	304	DPHVPNVTSLCLQYMKHDPNY
P97311	393	GTSLRGDINVCIVGDPSTAKS
P10518	132	CLCPYTSHGHCGLLSENGAFL
A2ASS6	20821	SNITNYIVEKCDVSRGDWVTA
Q8NBI5	63	PIGNATGQADCKAQDERFSLI
P53569	595	AFVKRLLQVTCTQMPPFICGA
Q8BH61	315	SSETPVRYGQCWVFAGVFNTF
Q9DCZ1	127	LEAVPQVKFICLDVANGYSEH
P35579	1379	MKKKMEDSVGCLETAEEVKRK
P12004	148	KMPSGEFARICRDLSHIGDAV
O70325	16	LSRLLKPALLCGALAAPGLAG
Q60597	231	EFMFINDLEQCQWIRQKFETP
Q99439	175	VIGLQMGTNKCASQSGMTAYG
Q922B2	203	QAIFRLQSGICHLFRETLINK
A6H611	279	FLYPNADQLKCLEELLSSRDL
P70388	221	KYLKQNKEKACEIRDQITSKE
P54822	173	AQLTTVGKRCCLWIQDLCMDL
P33992	207	CNTDQAGRPKCPLDPYFIMPD
Q71RI9	244	ADLCVKHDTLCISDEVYEWLV
P21817	2232	EIRFPKMVTSCCRFLCYFCRI
P12814	476	YYDSPSVNARCQKICDQWDNL
O89103	140	ASKSSCIFKRCVSLILDLSLT
Q3TXS7	141	EGIVNKMFQRCLDDHKYKQAI
O60443	407	PDSAAALLGTCCKLQIIPTLC
O70305	91	RPGSRRLLGVCGPPRPFVVVL
Q8JZN5	331	KLIELTAEYACTRKQFNRNLS
P50991	414	SIHDALCVIRCLVKKRALIAG
Q99KP6	230	SIPGILALDLCPSDTNKILTG
Q9Y6I9	182	IYQEDQIHFMCPLARQGDFYV
P30153	504	NYLHRMTTLFCINVLSEVCGQ
Q9Z1Z0	344	INTVSEVIRGCQVNQDYFASV
O35643	924	GNPSFTLSLKCRAPEVSQHVY
Q9H0C8	325	TPNDRFILLACDGLFKVFTPE
P15105	99	FRKDPNKLVLCEVFKYNRKPA
Q9H0C8	367	SAADARYEAACNRLANKAVQR
Q9ET80	318	WANNKRHGYGCTVFPDGSKEE
P06213	311	CHQYVIHNNKCIPECPSGYTM
Q91VR2	13	SRASVVGLSACAVQPQWIQVR

Q811U4	509	KKFDLSYDLNCHKLCSDFQED
O00468	399	CQGRDQCPEPCRFNAVCLSRR
Q99683	643	FQIYFCTELHCKKFFEMVNTI
P49717	305	IPEMQEAFFQCQVCAHTTRVE
Q99NB1	107	LGGQLNVSVNCLDQHVQKSPE
Q9DBM2	231	QYPGRLAPETCVRSVQASVKH
P17427	710	GSEDNFARFVCKNNGVLFENQ
P33992	219	LDPYFIMPDKCKCVDFQTLKL
O15519	310	PEHRDYDSFVCVLVSRGGSQS
P23610	62	QLGRELRAQECLPYAAWCQLA
P28474	268	TDGGVDYSFECIGNVKVMRSA
O60502	215	YLGEPETFLFCPTEYCGTFCY
O70572	251	LYKAVSEFHVCCETLKTTTGC
Q8VBW6	395	SNSAFLRVVRCRSLAEEYGLD
P26039	1202	VTQALNRCVSCLPGQRDVDNA
Q16665	359	DLIFSLQQTECVLKPVESSDM
P04075	290	ASINLNAINKCPLLKPWALTF
O08529	105	TDICQGALGDCWLLAAIASLT
Q9Z1Q9	562	DPRYQHLKGKCVVHPFLSRSL
P14550	187	SVRPAVLQVECHPYLAQNELI
P10833	213	PSAPRKKDGGCPCVLLXXXXX
Q5SSW2	737	STTLIYPTEYCSVPGGFNKPP
Q9WVQ5	210	GETWEKAKTMCECYDYLFDIA
P33992	221	PYFIMPDKCKCVDFQTLKLQE
A2ASS6	1018	AFAEDSGRFTCSAVNEAGTVS
Q62261	73	WVNSHLARVSCRITDLYTDLR
Q6PDN3	791	EILLKNRVGECSCQVSLMLHN
Q9Z1Z0	383	VNERQPFVLRCAVLYCFQCFL
P78527	1767	MLLELMTEVLCREQQHVMEEL
Q6PDN3	840	DGDRHGTLRPCWPARGQGWPE
Q15149	4071	NLQKFLEGTSCIAGVFVDATK
P61962	268	PVARLNNHRACVNGIAWAPHS
Q7L8L6	689	MEMAGLCPAACMQTPRMKLAV
Q9DCS9	77	QYRRVPDITECKEGDVLCIYE
Q9JHU4	4168	TFSSIPVSRICKSPNERARLY
O43447	174	KPKLPVVISQCGEMXXXXXXX
Q9DCN1	247	AEEFKQRHENCYFLHPPMPAL
A2ASS6	30983	RLNWVIVEGECLTASYVVTRL
P68104	370	VLDCHTAHIACKFAELKEKID
Q9CZ13	7	XXXXMAASAVCRAACSGTQVL
Q7L8L6	475	FNQYPEHLPTCLLGLAFLEYF
P21333	1225	GTHTITYIPLCPGAYTVTIKY
A2ASS6	17717	LKVSDITRGSCRLSWKMPDDD
Q8CG76	362	DQAWNMVAHECPNYFRXXXXX
O09110	120	DLDINMRTVDCFYTVTFYGAL
Q9NX47	97	DLADRLISKACPFAAAGIMVG
P78527	1954	AYNCAISVICCVFNELKFYQG
P40227	366	GEEKFTFIEKCNNPRSVTLLI
P35285	194	RQPSEPKRSCCXXXXXXXXXX
Q8BFQ4	141	IRLWDLRSPNCQGLMHLQGKP
P47809	380	ERTVEVACYVCKILDQMPATP
P70388	48	NGAGKTTIIECLKYICTGDFP

P22102	564	GIAKACGKAGCALLGGETAEM
Q99KC8	163	YQLSEQSANSCLNIQKPTVPL
O00170	90	MREGEIAQFLCDIKHVVLYPL
Q7TQ48	9	XXMKALLLLCCFLASLLLSGQ
P70398	1908	YKFDDGDVTECKMDDDEEMKN
Q08211	608	DDGGEDDDANCNLICGDEYGP
O60502	181	LLFDDIDHNMCAADKEVFSSF
Q8BKT7	507	EHGIVPVTSDCQDLFPAKVVS
Q99714	58	EAQAKKLGNNCVFAPADVTSE
P40936	76	GPTIYQLLSACEVFREIIVTD
Q810L3	641	SRPDCYWGRNCRTQVKAHHAM
P32921	10	XMADMPSGESCTSPLELFNSI
Q15274	27	ALVDSWLREDCPGLNYAALVS
Q9WU78	122	ALASLGYEKSCVLFNCAALAS
Q6PAR5	275	FIGYLKQNTYCFPHSLRWIVS
Q14684	155	FLDVLMKEVLCPESQSPNGVR
Q7KZF4	228	LVTVMLSGIKCPTFRREADGS
Q60714	392	QIGEFYGATECNCSIANMDGK
Q9ER72	472	AQSEAYFENDCWVRYFLHTGH
P70398	2107	SNRFSEYLLECPSAEVRGAFA
Q62234	1013	APSTVSECFKCEEWTIAVPGP
Q8JZQ2	27	GLPPLLVPRGCLGPDRRPCLR
Q9R112	379	PIKKYDGYTSCPLVTGYNRVI
Q9JHU4	2184	TALREELKKVCQEMYLTYGDG
Q9D7I5	23	GVLLDISGVLCDSSASGATAI
Q62418	321	APVSRPAAGVCEEPAPSTLSS
Q8BWT1	92	ETGALTLNRLCGSGFQSIVSG
A2ASS6	20798	FDISEIDADACSLSWHIPLED
O70546	1164	SVNINIGPGDCEWFVVPEGYW
Q9UQM7	373	NGDFESYTKMCDPGMTAFEPE
Q7KZF4	96	LRKKLIGKEVCFTIENKTPQG
P34932	140	ESVLKKPVVDCVVSVPCFYTD
Q71FD7	185	LPEREVSTDVCGFCHKPVSPR
P98156	312	DCVDGSDEVNCKNVNQCLGPG
Q501J6	505	NNPNLMYQDECDRRLRGVKDG
O70546	367	WMDLGTLYESCNQPQDAIKCY
Q99L45	303	TRLYFLQCETCHSRCSVASIK
Q99798	451	GIVLANACGPCIGQWDRKDIK
P31327	1337	CEMASTGEVACFGEGIHTAFL
Q8R3F5	233	CQVSNYLFPDCRVISGHLEAL
Q02257	33	DSGIHSGVNTCVPSVSSKGIM
Q3UMB9	344	KKFYKSLLDICKKVPAITLTA
Q15527	127	QKQGVEYVPACLVHRRRRRED
A2ASS6	15866	EPSEPSDPVLCREKLYPPSPP
O35459	158	YQKTFTVIEKCPKPVIAAIHG
Q5JTH9	517	PVMRKCLQSLCDLRLSPHFPH
P54136	638	LKVNMWRMLLCEAVAAVMAKG
A2ASS6	6571	VEPANAGKYICQVKNDGGVRE
P11172	72	QNAGISFDTVCGVPYTALPLA
P30042	153	LSTFAVDGKDCKVNKEVERVL
Q8K4Z3	109	SKSPPTVLVICGPGNNGGDGL
P49411	387	VMFSLTWDMACRIILPPEKEL

Q8N0X7	562	VWQGLECAAKCIVNNVSAETV
Q9CXY6	271	PLALNVAYRRCLQILAAGLFL
P13489	209	CQLEALKLESCGVTSDNCRDL
A2ASS6	22710	RKSYSTVTTKCHKCTYKVTGL
Q7TNV0	226	GTTRKSKQTKCPEILSDESSS
P62835	141	GQNLARQWCNCAFLESSAKSK
Q8VDC0	577	LFYARFLSHFCHDQKMVKHRE
P26039	116	KTVTDMLMTICARIGITNHDE
P78347	745	QKVENLFNEKCGEALGLKQAV
Q9QXX4	438	PLLAEIFAGGCAGGSQVIFTN
O94979	589	TGNFESAVDLCLHDNRMADAI
O00429	446	HEEMQRIIQHCSNYSTQELLR
O00468	585	PSECMLHVHACTHQISLHVAS
O75153	732	FSPGVRFPESCQDEVRDQKQL
A2ASS6	8802	NGINVIASQRCNITTTEKSAI
P50995	428	LEEGMLAVVKCLKNTPAFFAE
P00750	91	VPVKSCSEPRCFNGGTCQQAL
P16546	2441	QNLTREQADYCVSHMKPYVDG
Q6P8J7	288	GNMKRVFERFCRGLKEVERLI
Q99KP6	351	LTKVTDETSGCSLTCAQFHPD
Q9CXT8	454	AVDAETVRRVCTKYIHDKSPA
P12382	170	GLVGSIDNDFCGTDMTIGTDS
P97372	118	KPEVWTLKEKCILVITWIQHL
O14980	623	EILNNINTIICDLQPQQVHTF
Q9WUA3	722	SDDSICVLGICKRDLLFQPVA
O43143	750	VLTTKNYIRTCTDIKPEWLVK
Q3B7Z2	747	ECKEKQDWGSCPDIFXXXXXX
P62878	42	LWAWDIVVDNCAICRNHIMDL
Q5UIP0	2169	NDSPSGMQTRCVWSPLASPST
Q8BIJ6	33	GPARLPSRLGCLGMTRRLVVR
Q14181	392	LDAKHEQVENCLLTSPFEDIF
Q8BG51	502	SEFLTEAETICDVVCLVYDVT
Q3ULD5	453	GSYGAGNYGMCGRAYSPRFLY
Q3UH68	987	RRKSISGKKLCSSCGLTLGKG
P49327	1564	AQPTCPGAQLCTVYYASLNFR
Q60597	39	AAIRTFQQIRCYSAPVAAEPF
P62932	49	HLLCGATFHMCKESEHTLLCP
P26039	243	DKACEFAGFQCQIQFGPHNEQ
P21333	1018	IVGPSGAAVPCKVEPGLGADN
Q8BP47	176	YLQCVLSDDLCQCYNGVVLST
P62932	669	VTSMSEHLKTCPFNIVERKTD
Q9D7I5	88	EEVTAPAPATCQILKERGLRP
P26039	719	CALSTSQLVACTKVVAPTISS
Q924X2	562	LPFGKGLIKKCRTSPDAFVQI
P70670	149	APHSVQKSSVCPPHPLTSPPS
P62932	58	MCKESEHTLLCPLEQVPCLNS
Q9R1V6	399	ECKCEDTWSGCIMGDTGYYLP
Q99KC8	199	QHGIERVQSNCSLSPIQYLTD
Q08J23	321	AEGGRMVYSTCSLNPIEDEAV
O89103	366	VNTLGSFHCECWVGYQPSGPK
Q6PIE5	987	MYPLKVTWWFCAFPYSLLIFI
P55060	853	AVGITKLLTECPPMMDTEYTK

P55036	58	NVGLITLANDCEVLTTLTPDT
P16675	83	VVLWLNGGPGCSSLDGLLTEH
P34914	154	SQHFDFLIESCQVGMIKPEPQ
P63167	2	XXXXXXXXXMCDRKAVIKNAD
Q99P88	195	TGSGILNDSMCGGMQLLPDPL
Q99836	280	FITVCDYTNPCTKSWFWTRLA
P02730	843	MHLFTGIQIICLAVLWVVKST
Q9ET26	111	LSKIRAHVTSCSKYQNYIMEG
P53396	845	RKPASFMTSICDERGQELIYA
Q14181	556	YFVKDVLGCVCVNPGRLTKGQ
P07237	343	ELTAERITEFCHRFLEGKIKP
Q05586	436	VNGDPVKKVICTGPNDTSPGS
P35821	32	DIRHEASDFPCKVAKLPKNKN
O95573	503	NTGRVGAPLVCCEIKLKNWEE
P14873	2079	PSEARQDVDLCLVSSCEFKHP
Q8BVE3	312	RQEYALAMIQCKVLKQLENLE
Q8BGQ7	901	TVDNEAGKITCLCQVPQNAAN
O89103	261	GPLCVSPKFGCSFNNGGCQQD
Q9EQ80	336	AASKGINVILCEHSNTERGFL
Q9ET26	95	IESIETSCHGCRKNFILSKIR
P97807	374	IMPGKVNPTQCEAMTMVAAQV
P58404	589	DPSSSGPSCLCTFPMDGEHGI
P62736	12	CEEEDSTALVCDNGSGLCKAG
Q921M4	615	AQGLQEQRDQCLSHLQQYAAA
P48643	429	VYGGGAAEISCALAVSQEADK
Q08211	973	ILINSGFPEDCLLTQVFTNTG
Q60770	501	DSKEWPYCSRCPAVWNGSGAV
P21817	565	ASSGILEVLYCVLIESPEVLN
Q9ESD7	1255	GADEFMGRCICQPSLERMPRL
Q8VCI5	296	SGPPGANGEQCLIMXXXXXXX
Q9D7P6	131	IKNTDIAKELCLPPVKLHCSM
Q6PGB6	61	FNDIAVGAVCCRVDHSQNQKR
Q9R0H0	418	PNIYVTFTPACTFEGENTVMM
Q6ZWY3	64	VLCVGCSTVLCQPTGGKARLT
P23975	520	GFRPGLYWRLCWKFVSPAFLL
Q8BWM0	205	NDQGKEVTEFCNKYWLMLDEK
Q8R3F5	223	CKSLGIENPVCQVSNYLFPDC
P98156	421	INLKGGYKCECSRGYQMDLAT
A2ASS6	30448	LTGITNQLITCKAGSTFTIDV
O54931	856	DSSYTSKLLSCKVTSEVLEAT
A2ASS6	9210	ASMADAGLYTCKATNDAGSAL
Q9UQM7	199	PYGKPVDLWACGVILYILLVG
A2ASS6	29480	PEDDGGGEITCYSIEKREASQ
Q64436	240	ESEPQTRSPECTHESPLETRN
Q9NVM4	623	LWMEYHLTPECTLSTGLLEPA
O15382	22	RKLLSVPWLLCGPRRYASSSF
P35754	26	VVFIKPTCPYCRRAQEILSQL
O35459	170	KPVIAAIHGGCIGGGVDLVSA
A2ASS6	27791	TSVTKDSMTLCWSRPETDGGS
P00750	291	LKNRRLTWEYCDVPSCSTCGL
P98156	246	HTKCPTSEIQCGSGECIHKKW
Q86VP6	940	ENIWALLLKHCECAEEGTRNV

Q9CRA7	58	DRAASEWLLRCGAKVRYCGHQ
Q91WU5	376	TGGCCGKRKNCXXXXXXXXXX
A2ASS6	7675	DGNEIISSPKCQSSFADNVCT
P32020	115	HQLIQGGLANCVLALGFEKME
Q8K298	807	SKGSVTLSEICLPLKADFVCS
P53996	133	CGEFGHIQKDCTKVKCYRCGE
Q6R5N8	777	SLLIDFDDAMCNFDLGKVYFL
Q9WVJ2	182	YYKDALRFLGCVDIKDLPVSE
A2ASS6	4884	VQISLGGKYTCLAENEAGSQT
Q9CQM9	161	FMKGTPQEPRCGFSKQMVEIL
Q64514	456	NGTSMSSPNACGGIALVLSGL
Q71FD7	213	KRQYHAQCFTCRTCRRQLAGQ
P00750	509	GGPQANLHDACQGDSGGPLVC
Q7KZF4	736	GSYAPRRGEFCIAKFVDGEWY
Q64310	81	FLNLLGQLTGCVLVLSRNFVQ
P19096	1464	RKEPGGHRIRCILLSNLSNTS
P21333	210	ALVDSCAPGLCPDWDSWDASK
Q6PIE5	241	HENPLETRNICFFSTNCVEGT
O75828	4	XXXXXXXMSSCSRVALVTGAN
P21817	1150	RPWQPGDVVGCMIDLTENTII
P14873	1305	GVTQAVVEEHCASPEEKTLEV
B1AR13	110	QETRTVALCTCKATQRPPYCD
Q9R0P3	176	YRSVSAFAPICNPVLCSWGKK
Q99615	58	YNYYTKAIDMCPKNASYYGNR
Q8K2B3	305	FHPTGIYGAGCLITEGCRGEG
Q6NSR8	73	PTDSCPLYLNCATVAALPSRV
A2AVZ9	51	NYFSEPCEQDCLLQSNVTGPS
Q9D8B4	75	AIGAMFGLTTCVSAQVREKPD
P05455	18	EKMAALEAKICHQIEYYFGDF
Q7TNV0	352	NLEEVTMKQICKEVYENYPAY
Q8BKT7	208	RKRLAEKYRECLSNKEKILKE
P97478	34	SEYGRGLIIRCHSSGMTLDNI
Q6NSR8	357	LVLADGVSYACKDLGADIIVD
Q9GZT4	6	XXXXXMCAQYCISFADVEKAH
O70546	562	SVSQPGVHTACPRQTLANGPF
Q6R5N8	340	RHGHLDMKTVCHLLGNLPKLE
O00220	274	IGSGCGGDPKCMDRVCFWRLG
Q99J09	53	LLGVSSLSGRCWVGSLWFFKD
P70168	358	AGVCLMLLSTCCEDDIVPHVL
P28474	60	YTLSGADPEGCFPVILGHEGA
Q15149	3008	NLTYLQLLERCVEDPETGLCL
Q9DBC0	142	PGTEPAAHCYCGHQFGQFAGQ
P50991	337	DIEREDIEFICKTIGTKPVAH
O00468	814	TCDPATGQCSCRPGVGGLRCD
P45952	116	CLITEELAYGCTGVQTAIEAN
P63318	66	IWGIGKQGLQCQVCSFVVHRR
P97447	101	KCATREDSPRCKGCFKAIVAG
Q13144	571	LQRGKEENISCDNLVLEINSL
P21333	574	SPFEVKVGTECGNQKVRAWGP
P06213	331	MNSSNLLCTPCLGPCPKVCHL
Q3UH68	1016	NLYFHIQCFRCGICKGQLGDA
O75828	26	GIGLAIARELCRQFSGDVVLT

Q91WD5	326	FDVPIGSRGDCYDRYLCRVEE
Q6PDY2	96	LGVFLLKSGTCIPLHDHPGMH
A2ASS6	22120	MKANHVNVPDCAFTVTDLVEG
Q9WU78	90	PKFPFSENQICLTFTWKDAFD
Q8BXG3	150	YGEQVCYVLDCLAEEALKYIG
Q9DC61	19	ARLLRGSAVLCARPRFGSPAH
A2ASS6	17839	GSPVTHYIVECLAWDPTGKKK
O00468	513	GSDGVTYGSACELEATACTLG
P60603	27	DRVKMGFVMGCAVGMAAGALF
Q12931	261	TKIIIHLKSDCKEFSSEARVR
Q62234	279	VWEKENVKLHCSVAGWPEPRL
Q9DCZ1	224	GHIISDGGCTCPGDVAKAFGA
Q6NXE6	201	EADLTCSGIRCVRHACLKHEQ
Q8BUV3	313	VGSLKARLPSCSSTYSVSEVQ
P42932	430	AKQITSYGETCPGLEQYAIKK
P40937	152	IEKFTENTRFCLICNYLSKII
Q8R5K2	850	IDNTKIAVTKCGSVMLKQGAD
P41250	231	HLQKLMSDKKCSVEKKSEMES
P16332	469	EGIPKLRIEECAARRQARIDS
Q6PAR5	690	AAENVLGSLLCLPGSGSVLLD
P14873	73	IRSWDTNLIECNLDQELKLFV
A2ASS6	21561	GKYILTLENSCGKKEYTIVVK
Q62095	54	LRNRETSKGVCDKDSSGWSCS
O94927	415	KGNSASKTRLCRSPGEVLALV
Q6IFX4	127	ADLEDKIQEACSKALPILCPD
Q8BK08	129	LYGISWQFDPCCKYQVEYDAY
Q8BW75	297	SRVPLGSVIKCMVYYKEPFWR
P45952	95	GLINAHIPESCGGLGLGTFDA
Q8C1E7	12	QSPPPDPLGDCLRNWEDLQQD
Q9D517	104	LNHNFEIDFLCGWTMCERFGV
O75369	2154	EIVPMGKNSHCVRFVPQEMGV
Q29RF7	583	LELLISPTCSCKQADICVREI
Q64277	137	YGKVGDFLSWCRQENASGLDY
Q9R1V6	521	REICSGNSSQCAPNVHKMDGY
Q29RF7	532	LHKQPTSEANCSAMFGKLMTI
Q60714	394	GEFYGATECNCSIANMDGKVG
P12268	173	IDFLKEEEHDCFLEEIMTKRE
P17426	970	TSKEPVSRHLCELLAQQFXXX
Q80UJ7	464	SLTYKLALCLCMINFYHGGLK
P53618	816	DVSGAASDRNCVVLSDIHIDI
Q9WVQ5	4	XXXXXXXMSGCQAQGDCCSRP
Q61024	13	GIWALFGSDDCLSVQCLSAMK
P06213	495	ALKTNGDQASCENELLKFSYI
Q8BFR6	240	TLERWITKEECPLYNGGNVIL
O70468	1120	TVLEHYRRTHCVVSELIIGNG
O14920	444	WHSIQTLKEDCNRLQQGQRAA
P78527	1919	NELTKTLIKLCYDAFTENMAG
O15519	393	VEFKAQKRGLCTVHREADFFW
Q3U1J4	680	NVNLKEVNYMCPLNSDGYPDS
Q9CR24	159	QTQNQGYTHFCQGGHFSYTLP
Q61151	71	ELFLKKLQQCCVIFDFMDTLS
P17751	40	ISGQWREPCVCTDLQRLEPGT

Q8BXG3	88	TNPGEQFYMFCTLAAWLINKT
O88712	350	TGRIPDSLKNCVNKDHLTAAT
Q8WX93	1022	IFEGMPVTFTCRVAGNPKPKI
P53996	112	CGKPGHLARDCDHADEQKCYS
P70388	681	TQLTDENQSCCPVCQRVFQTE
P10605	179	SGGVYNSHVGCLPYTIPPCEH
O14744	42	AVAKQGFDFLCMPVFHPRFKR
Q8K2M0	290	NFSEDTRPSPCYQLAQRTFRT
P23528	39	VKKRKKAVLFCLSEDKKNIIL
Q8QZZ7	13	LSQQLDLFPECRVTLLLFKDV
Q9CZR8	14	LRSLRFFPVACTGRSARAVLL
P49717	571	ALVLSDNGICCIDEFDKMNES
A2ASS6	8215	VKQDEYTRYECKIGGSPEIKV
Q6ZPY7	927	GKWGIKANCPCISRQSKSVLR
Q9DBL1	22	GGLLRRRFPTCLSPWKIPPRV
Q12879	870	LFSISRGIYSCIHGVHIEEKK
Q9JKX6	130	GEVAECSPAVCMDPGLSNCTT
Q15369	74	EIPSHVLSKVCMYFTYKVRYT
Q9UNE7	69	VAVYYTNRALCYLKMQQHEQA
P97823	169	SANRDISVLQCHGDCDPLVPL
Q8CGY6	84	SADIKALYRRCQALEHLGKLD
Q8K1X1	39	WGWQGLIAYGCHSLVVVIDSN
Q96I24	148	KRLLGQIVDRCRNGPGFHNDI
Q62234	1438	EAFQDLMTEVCKKIALSATDL
Q91YE3	243	ITWIEGKEPGCETIGLLMSSM
P30101	57	LMLVEFFAPWCGHCKRLAPEY
P14873	2037	TTRSPDTSAYCYETMEKITKT
P21333	1410	MSCMDNKDGSCSVEYIPYEAG
Q9QYJ0	189	VQQMQSVCSDCNGEGEVINEK
Q9ET26	57	HVFCSACLQECLKPKKPVCGV
Q9UDY4	7	XXXXMGKDYYCILGIEKGASD
Q3TXS7	104	SEYVETIIAKCIDHYTKQCVE
Q8BZA9	252	CEEARQPSIQCVCMNLQEHLN
O00468	546	RCGQCRFGALCEAETGRCVCP
P41250	461	TSYGWIEIVGCADRSCYDLSC
Q8TAT6	208	LPWPNGICTKCQPSAITLNRQ
Q9NPH0	416	TLSPEKYHALCSQTQVMEVGN
Q91YP0	38	GPASGVPGLLCGGGRRSSSTS
Q6PAR5	176	NPRRLLRRGTCAFSILFKLFS
Q6ZPY7	217	YQPEGEEVWLCGVVSRQDSVT
Q92616	1161	MMGLDLQPDLCSLLIDDVIYH
Q06203	496	MIQENGNGLECFEKSGHCTAC
P12382	89	LGGTIIGSARCKAFTTREGRL
Q6A0A9	529	SHMGTVQPIPCLLSMPTRNHM
Q99NB9	965	ISRTAVVMKTCQEEKLMGHLG
Q8VDK1	116	LGQYSQLARECGIWLSLGGFH
Q8R035	150	SRYQFRNLAECLQKIRDMIAE
Q99JY9	235	KAVKERYSYVCPDLVKEFNKY
P02463	1570	VHSQTIQIPQCPNGWSSLWIG
Q9NYY8	269	QERINECDEICLSVLSTVLEA
Q8BKY8	32	PALKIKPSSACVTYGTDSQSD
P61247	96	LITEDVQGKNCLTNFHGMDLT

A2ASS6	15693	ITKDAVTLTWCEPDDDGGSPI
Q29RF7	742	TPHQAKQAVHCIHAIFTNKEV
O08749	306	ASGGKAEVITCDVLLVCIGRR
Q62312	163	RAGETFFMCACNMEECNDYII
P04117	2	XXXXXXXXXMCDAFVGTWKLV
Q9DCL9	81	AGIKTAFTKKCGETAFIAPQC
Q8BGC4	204	KIAKCHVIGTCSSDEKAAFLK
Q99JW4	74	QMLFAPCCHQCGEFIIGRVIK
Q62188	448	ELRGAPLVVICQGKIMLEDGN
Q8CGY6	786	QLRQAATECMCNMVLNKEVQE
Q91W50	464	AEDGIIAYDDCGVKLTIAFQA
Q9JI57	54	ALSKLNTEVACVAVHNESVFV
Q8TAT6	194	ENISCKIKSGCEGHLPWPNGI
Q92879	443	FIDKQTNLSKCFGFVSYDNPV
Q8C7B8	531	WVGHPLDPIGCLCRALLEACR
P50136	261	ATLECPIIFFCRNNGYAISTP
P21981	143	GHFILLYNAWCPADDVYLDSE
Q69Z37	570	QVFNGMDNMLCICVNSAIYQQ
Q9R1V6	823	IPDTKHISDICENGRPRSNSW
Q8K0S0	267	LDIACNKFLTCSVEDGELIFR
P47934	169	PVEFLGGQPLCMNQYYQILSS
P63005	330	IKMWDVSTGMCLMTLVGHDNW
O08749	312	EVITCDVLLVCIGRRPFTQNL
Q6ZPY7	1271	RDFWDGFEIICKRLRSEDGQP
Q99L85	119	SLSWLLCHIPCVTLCQALHAL
Q8R5K2	560	KGDNMYSCEKCKKLRNGVKFC
P99028	41	VREHCEQLEKCVKARERLELC
Q01320	169	GGRNGYGAKLCNIFSTKFTVE
O89103	325	HSVPLSENYTCRCPSGYQLDS
Q60870	114	SWLPFYYMLKCGFLLWCMAPS
Q6NXE6	329	LSILVSLLADCNDHQMRDQSG
O95573	504	TGRVGAPLVCCEIKLKNWEEG
Q8R164	225	QFKQLPEGNICRHLLPLVQCP
Q8R2Y8	113	SPQVLKEWEYCGQPKVVVKAP
Q9Y272	171	EQLVGDDPQRCAYFEISAKKN
Q9ER72	608	MEEMRALVSQCNLYMAARKAE
Q69Z37	419	YDWYILVTNTCAPTQLEHLEF
Q9ER88	91	MMQVKTFGEACLMVRKPALEL
O70433	212	ARDEFPYCLTCFCDLYAKKCA
O89103	408	INTDGSFYCSCKEGYIVSGED
A2ASS6	8370	KATNDVGSDTCVGSVTMKAPP
Q9R1V6	567	GQKVTASDRYCYEKLNIEGTE
P54886	88	GSAVVTRGDECGLALGRLASI
Q13144	372	LGSGTVIGSNCFITNSVIGPG
Q00987	461	VHGKTGHLMACFTCAKKLKKR
Q08211	558	TSMFCEYFFNCPIIEVYGRTY
Q99J09	73	DPSAAPNEGFCSAGVQTEAGV
Q15149	68	RGLVRETFAWCHFYWYLTNEG
Q99873	350	TIDLDFKGQLCELSCSTDYRM
Q922B2	76	VHTSRAKGKQCFLVLRQQQFN
Q9D1P4	24	FDPEANSDDACTYHPGVPVFH
P56399	195	QLDNPARIPPCGWKCSKCDMR

O70145	40	SEVQDPHSRICFNIGCVNTIL
Q92616	1482	NQYVREAADDCAKAVMSNLSA
P62736	19	ALVCDNGSGLCKAGFAGDDAP
A2ASS6	23887	QSKGSDRWATCATVKVTEATI
A2ASS6	34726	TNSAGSVSSSCKLTIKAVKDT
Q9CPR5	48	RPRDRRRGRKCGRGHKGERQR
P48725	2370	QKELQIEASRCEALLAQEKGQ
Q8K3K7	92	GQKKLEVDGPCVIISNHQSIL
P26641	166	RVTLADITVVCTLLWLYKQVL
Q8VE38	144	HPPAVWVHNKCTLDSEVALRV
A2ASS6	9415	TTKTDSGLYRCVAFNKHGEIE
Q8JZN7	558	HRLPAPASFSCLGPAMPSTDV
P36552	270	GNTHWWFGGGCDLTPTYLNQE
Q8R5K2	500	SHPTIVKAGSCGEAYAPQGWI
Q9CPY7	129	ENIRAAVAAGCRQVQDLELPS
P26638	395	GSGAFRELVSCSNCTDYQARR
P13707	162	IASEVAEEKFCETTIGCKDPA
P46459	582	KMIGFSETAKCQAMKKIFDDA
A8C756	674	SQSPGVRQQICSLLKKLFCRI
Q80Y14	118	VYLNGEFVGGCDILLQMHQNG
Q8QZR5	349	QMAKLMSVRLCPPVPGQALMG
P35235	567	TSGDQSPLPPCTPTPPCAEMR
P50579	290	VKDATNTGIKCAGIDVRLCDV
Q99JW4	164	YHPDHFNCANCGKELTADARE
Q8NBI5	363	TGSSTLAVALCSTVPSLALTS
P17405	89	RDVFGWGNLTCPICKGLFTAI
P30101	85	KGIVPLAKVDCTANTNTCNKY
Q8C7B8	248	TAGAGTEDANCWHLDEEQIQE
Q64514	678	EGATWAEVTVCSCSSEVSAKF
O00468	210	SPCPSVVAPVCGSDASTYSNE
P10605	187	VGCLPYTIPPCEHHVNGSRPP
A2ASS6	7684	KCQSSFADNVCTLTLSSLEPS
Q61024	43	RFENVNGYTNCCFGFHRLAVV
Q6PGB6	100	TKMLNHVLNICEKDGTFDNIY
Q6P2B1	511	PVLGYLMKGLCEKPLASAAAK
P00750	179	IRLGLGNHNYCRNPDRDSKPW
Q3ULJ0	221	VGAGFCDGLRCGDNTKAAVIR
Q9CQ65	163	IETAKKLGLRCHSKGTIVTIE
P31327	697	VVRHLGIVGECNIQFALHPTS
Q71FD7	237	QKDGRPLCEPCYQDTLEKCGK
P49442	293	PAAGAGYKSLCVIQGLADIYI
Q8BVE3	235	NCIMGVLSNKCGFQLQYQMIF
P61962	61	GLDEESSEFICRNTFDHPYPT
Q8VCW8	77	SHLVNTTVGECLDATAQRFPD
P70168	228	IMQVVCEATQCPDTRVRVAAL
O00468	1624	ECPLGREGTFCQTASGQDGSG
Q9QY76	173	DTEVKKVMEECRRLQGEVQRL
O43175	295	GASTKEAQSRCGEEIAVQFVD
Q9DC61	326	LTHIMVGLESCSFLEDDFIPF
Q811U4	111	LPSGIGHTTNCFLSVEGTDGD
Q9ER60	1183	VNLFAGKFYYCINTTTSERFD
Q69Z37	830	YEKTLVIILNCMRSQNPDESA

Q8R016	78	KSSGRCWIFSCLNVMRLPFMK
Q8VBZ3	489	LSWILFPLLGCYAVYSLLYLE
P98156	140	VSWRCDGENDCDNGEDEENCG
Q9QYC0	430	SFASDGDSGTCSPLRHSFQKQ
Q8R5K2	185	CPPLTQFFLDCGGLARTDKKP
Q8K3K7	48	LSFSAAASIVCLLRHGGRTVD
Q8BKC5	944	QFGGDNYRPFCTDALPLLVRV
Q8K298	412	IQERLFKQNTCSSTTHLAQQL
Q62095	316	VQQIRDLERGCHLLVATPGRL
Q922E4	110	ETLDKHNCDFCVHGNDITLTV
Q4G0N4	3	XXXXXXXXMTCYRGFLLGSCC
Q8TEX9	269	LGNAIRIRILCCLTFLVKVKS
Q9ET26	92	ERQIESIETSCHGCRKNFILS
P30048	60	QAKLFSTSSSCHAPAVTQHAP
Q9NVM4	639	LLEPADPEGGCCWNPHCKQAV
Q61768	421	GSFTDAERRKCEEELAKLYKQ
Q8BVZ1	80	SACRLAEHCVCSVTTCALDHA
Q6ZPY7	715	CINVAPHLHKCRECRLERYRK
P06213	674	EDSELFELDYCLKGLKLPSRT
A2ASS6	18374	AISSSMVIKNCQRSHQGVYSL
P36405	158	IRDRVWQIQSCSALTGEGVQD
Q3UMB9	154	LQELSCFVTRCYEVVMNVIHQ
P23368	12	LSRLRVVSTTCTLACRHLHIK
P15105	49	GLRCKTRTLDCEPKCVEELPE
Q91W50	680	ITPLRRATVECVKDQFGFINY
P61922	166	SQLITMACGSCSNENAFKTIF
Q9NPH0	41	LAELQEADGQCPVDRSLLKLK
Q6ZQ73	953	RCESPEEGTRCVVAECIGKLV
Q9EPL8	827	FITQWLNDVDCFLGLHDRKMC
P70398	540	SAHIKILDYSCSQDRDTQKIQ
P32322	120	AFRPAPRVIRCMTNTPVVVRE
O00468	473	QAPSPCLGVQCAFGATCAVKN
Q9WU65	37	VFNSKTAELVCSHQVELTQEY
P70398	697	NAVYLCDREACFKWYSKLMGD
P53621	1191	PVEKCPLSGACYSPEFKGQIC
Q14003	308	FFILISITTFCLETHEGFIHI
Q8CHT0	350	FEYGGQKCSACSRLYVPKSLW
Q9JI91	781	GLMDHEDFRACLISMGYDLGE
Q9JHR7	178	FFLCPLLDASCKDREVNAVDS
Q8K297	278	CKQAEVQMYVCNKEVYGFLPV
Q9UQM7	64	HQKLEREARICRLLKHPNIVR
Q99NB1	445	TWWQTETGGICIAPRPSEDGA
Q14166	370	PCENLLTVKDCLASIARRAGG
P62932	88	RHKLAKHLQVCPASVVCCSME
P21266	190	DEFPNLKAFMCRFEALEKIAA
Q9JHU4	1295	FGRLKDDREKCAKAKEALELT
Q80YD1	549	FAQVDGQYFVCNMDDFKFSAE
Q9R0P3	181	AFAPICNPVLCSWGKKAFSGY
Q8N0X7	272	LNRPPGFLQVCDWLYPLVPDR
Q7TNG8	106	GTGTGVEGGVCAVQGGVCINL
A2ASS6	15589	FDITDVTNESCLLTWNPPRDD
Q3B7Z2	74	YRSKAEMRHTCRGTINLATAN

Q9P2E9	1057	QAKEESEKQLCLIEAQTMEAL
Q9R1V6	472	AECALEGAECCKKCTLTQDSQ
P48200	618	SGNKNFEGRLCDCVRANYLAS
P16546	1091	EKRKGMLEKSCKKFMLFREAN
P53621	254	TCRGHYNNVSCAVFHPRQELI
Q8R016	73	PVTNQKSSGRCWIFSCLNVMR
A8C756	1044	DVTAQMVLACCWRSMKEVALL
Q29RF7	971	VKERRAHARQCLLKNISIRRE
Q6ZQ73	455	KDRNVRTRQGCFNLFTELAGV
O43175	421	AAPGEQGFGECLLAVALAGAP
P45952	20	RVLRSVSHFECRTQHSKAAHK
O14980	595	CDTFIKIAQKCRRHFVQVQVG
O08992	167	DQVLQINGENCAGWSSDKAHK
Q8VC74	302	LLRSPGFRQLCRIPPSKSDSE
Q5U458	241	LTPRCFVTTNCALQFSSRGIR
Q8BUV3	133	TPLGMLSRPVCGIRGKTLIIN
Q9R1V6	677	LPVASFNFSTCSSSKAGTVCS
Q8BP48	202	SCCTSVNEVICHGIPDRRPLQ
Q8BKT7	608	IRAMESEVNVCYKELCGPRPS
A8C756	727	QQYKNFMSSVCNILFEALFPG
Q8BUN5	394	YRRQTVTSTPCWIELHLNGPL
Q9CXI0	170	DSLGGSLATVCDINREMLKVG
P17812	234	TSVKEKISMFCHVEPEQVICV
P23610	69	AQECLPYAAWCQLAVARCQQA
P13489	220	GVTSDNCRDLCGIVASKASLR
Q9DB43	24	LFCFEHRVNVCEHCLVANHAK
Q02257	204	QNTSDLDTARCTTSILHNLSH
Q8CC88	443	MVQSHMVKDICLIGGKGCGKT
Q9CW46	255	VYTPTFCQLACGQDGQLKGFA
Q6R5N8	867	EQGSQTTFKLCLHQRDFEPGI
O00468	183	RGMLCGFGAVCEPNAEGPGRA
Q8K298	1004	SGFGAWHRRWCVLSGNCISYW
P22314	1040	HVRALVLELCCNDESGEDVEV
Q9D051	161	SAGVAAQHSQCFAAWYGHCPG
P07814	623	TTHALPIPVICVTYEHLITKP
Q92616	1446	EGALFAFEMLCTMLGKLFEPY
O14744	278	GTNHHSEKEFCSYLQYLEYLS
A2ASS6	6827	GARELVKGNRCNIYFEDTVAE
Q8CGY6	368	LINKLYDDLRCDPERDHFRKI
Q5VYK3	1806	KKLEESKQWECLTSECRVLLI
A2ASS6	4800	ECVVANEVGKCGCVATHLLKE
O89103	30	GAAADSQAVVCEGTACYTAHW
Q9WV34	232	PHLPRQVFVKCHFDYDPARDS
P06213	335	NLLCTPCLGPCPKVCHLLEGE
O88545	140	PSDIHVHKQVCEIIESPLFLK
Q9NYY8	265	LRVTQERINECDEICLSVLST
Q8BG51	335	TFDKHDLDRDCALSPDELKDL
A2ASS6	3592	LASNEYGKAVCSAHLRISPRG
Q8VEM8	272	FVAGYIAGVFCAIVSHPADSV
P62192	399	DLSGADIKAICTEAGLMALRE
Q9R1P4	148	DDMGPHIFQTCPSANYFDCRA
Q9BQG0	890	THHLCRARRYCHDLGERAGAL

P17987	218	SMLISGYALNCVVGSQGMPKR
Q9C0B1	456	QNLRREWHARCQSRIARTLPA
Q3UZY0	1216	ARVRALRRALCXXXXXXXXXX
P16615	1010	PATKSCSFSACTDGISWPFVL
P17405	250	GAGYWGEYSKCDLPLRTLESL
O00468	1843	VPREAAYVCLCPGGFSGPHCE
O35309	89	KDGCHFSNSSCSFQVSSQILY
Q62261	624	AHMEFCYQELCQLAAERRARL
Q64514	221	DSNENGDLSKCAVLRNYKEAQ
Q810L3	545	PFCELNLGDKCLDGVLNNNNY
Q8K298	258	SSSVQQEATCCSPRDGNASVR
Q8R5A6	365	LRNIEADTYWCMSKLLDGIQD
P29474	368	MSTEIGTRNLCDPHRYNILED
P68104	31	STTTGHLIYKCGGIDKRTIEK
Q9CQT1	168	RGGKVTVLTHCNTGALATAGY
P17563	466	AHELRYPGGDCSSDIWIXXXX
Q8K1A5	163	CLCSGLGASFCYMLSYLVGRP
P43243	319	HINGASHSRRCQLLLEIYPEW
A2ASS6	5457	QAKNDAGIQRCSALLSVKEPA
Q9WU78	76	ETLLRYYDQICSIEPKFPFSE
Q8R016	269	KPLFNMEDKICFVNDPRPQHK
O00468	479	LGVQCAFGATCAVKNGQAACE
Q92616	1692	GAMVKGMGESCFEDLLPWLME
Q9DCN1	284	VLAWHSRYKFCPTCGSATKIE
Q3TCJ1	14	SISGYTFSAVCFHSANSNADH
Q3U1J4	1008	FHLGEFVNVFCHGSLVMQNLG
Q6PEB6	110	MTATEQWIFLCAAHKTPKECP
P78527	974	RTFPVLLRLACDVDQVTRQLY
O00468	537	QVARKGPCDRCGQCRFGALCE
Q9CRB2	92	AGDTLPIEVYCHLPVLCEDQN
Q4G0N4	311	EKQKSSGLNLCTGTGSKAWSF
P35579	740	PKGFMDGKQACVLMIKALELD
P41091	434	LGKIVLTNPVCTEVGEKIALS
O43175	200	QLPLEEIWPLCDFITVHTPLL
Q00987	362	QAEEGFDVPDCKKTIVNDSRE
P35821	426	ATLLATGAYLCYRVCFHXXXX
Q64337	128	PRNMVHPNVICDGCNGPVVGT
A2ASS6	5022	SVVGGACRLDCKIAGSLPMRV
Q8VBW6	34	GQEALESAHVCLINATATGTE
Q61792	32	DKYWHKACFHCETCKMTLNMK
P01009	17	WGILLLAGLCCLVPVSLAEDP
P29218	125	KKIEFGVVYSCVEGKMYTARK
P35247	365	FTNGKWNDRACGEKRLVVCEF
Q62433	72	YHDIGMNHKTCYNPLFNSEDM
Q8C5H8	279	GESLSSRMPYCWAVAVDNLRR
Q99NB1	326	FDYQPGDVFGCVADIGWITGH
Q99NB1	345	GHSYVVYGPLCNGATTVLFES
Q9C0B1	249	RSAVAVYSYSCEGPEEESEDD
P30556	149	RRTMLVAKVTCIIIWLLAGLA
Q9BXJ9	448	DTADRFINSKCAKYMLKANLI
Q14103	252	KKYHNVGLSKCEIKVAMSKEQ
Q8R349	245	VSLAERHYYNCDFKMCYKLTS

P80314	535	PRKRVPDHHPCXXXXXXXXXX
Q92769	417	SIRASDKRIACDEEFSDSEDE
P70310	322	LPYHARRLMYCYIPDDGWTDE
Q9D289	69	KDELDIMKFICKDFWTTVFKK
P21981	285	GQCWVFAAVACTVLRCLGIPT
Q9WTI7	585	CSSMNPIMAQCFDKSELSDKK
P31948	282	KGDYNKCRELCEKAIEVGREN
Q8C460	116	LLSPCGVPELCSISTRKLAAH
Q3UKJ7	416	LPKNPEHFVVCNRSNTVVIMN
Q5VYK3	1239	KTLSKVCVKMCDPAKGAAGQR
P48200	320	VSLTLPEVVGCELTGSSNPFV
Q3TL44	452	TYFSEEDVRGCLEAGIKTEEE
Q9WV60	178	SLAYIHSFGICHRDIKPQNLL
A2ASS6	13260	DQAGEVLYQACNAITTAILTV
P37268	374	STIRTQNLPNCQLISRSHYSP
Q9JIK5	517	SGHQGRTIIFCETKKDAQELS
O14980	164	VGASRTSESLCQNNMVILKLL
Q91YR7	913	NAEPRHGELWCAVSKDITNWQ
O88544	232	RLEALKHALHCTILASAGQQR
P08397	261	RAFLRHLEGGCSVPVAVHTAM
P50579	263	GTHISGRIIDCAFTVTFNPKY
Q8NBI5	47	FKNEDYFKDLCGPDAGPIGNA
P60900	115	YGYEIPVDMLCKRIADISQVY
Q14139	929	YLNLGDEENFCATVPKDGRSY
P00750	127	KCCEIDTRATCYEDQGISYRG
P00533	775	VMASVDNPHVCRLLGICLTST
P97314	33	VQCDGRSFHRCCFLCMVCRKN
P51787	214	IVVVASMVVLCVGSKGQVFAT
P98156	45	SSQFQCTNGRCITLLWKCDGD
P27635	105	HVIRINKMLSCAGADRLQTGM
Q1XH17	14	APGLLRQELSCPLCLQLFDAP
Q06587	84	VTALRSGNKECPTCRKKLVSK
Q8R1S0	120	GAWDHICNMRCKAFRRMQVWD
Q5SSW2	537	NDLTEIEKELCSATAEFEDFV
Q922B2	349	FLEPTLRLEYCEALAMLREAG
Q3B7Z2	288	GSAGSGKDQCCSGKGDMSDED
P23528	80	TFVKMLPDKDCRYALYDATYE
A2ASS6	14323	AQLEDAGSYNCRLPSSRTDSK
O00468	969	LQISIQSLGPCQEAVAPSTHP
Q9CR21	9	XXMASRVLCACVRRLPAAFAP
O94927	533	LLLQDQRSLWCWDLLHMKTSL
Q9D6Y9	81	RGYESFGIHRCSDGGIYCKEW
Q7TPD0	957	LQALQHVQASCDEAHKMKFSD
Q8BGX2	79	YVGLLGGAAACCALAPSEAAF
P13707	265	SCGVADLITTCYGGRNRKVAE
Q3UQ84	120	YDLDRPLETDCHLRFLTFDSP
O55222	108	EHGNVPLHYACFWGQDQVAED
P06213	280	PPYYHFQDWRCVNFSFCQDLH
Q8K2B3	467	LLDLVVFGRACALSIAESCRP
Q62407	94	LLPPPAPEPSCLWLRSCGAQD
P10518	119	RKTFPSLLVACDVCLCPYTSH
Q8R2L5	38	TQAVSVIWRRCFSQFEQVTSN

P10637	227	AAPQAGSGSVCGETASVPGLP
Q16658	226	FRSGKVAFRDCEGRYLAPSGP
Q91YR7	901	EEQQEEVRKRCENAEPRHGEL
P67775	55	VQEVRCPVTVCGDVHGQFHDL
Q62523	439	YTDTLEKCNTCGQPITDRMLR
Q9JHK4	256	CCLHVSREEACLSVCFSRPLI
Q9ER60	501	ADGDPTHSKDCNGSLDTSGEK
Q8BIJ6	987	VIVVPTAREKCPRCWKHTSET
Q60759	115	VLGPTIKGYGCAGVSSVAYGL
Q9JJX8	252	VERVHYSSTWCEGMVSLLKKL
A2ASS6	20896	NARVTKVNKDCIFVAWDRPDS
P48039	100	NNGWNLGYLHCQVSGFLMGLS
Q5JTH9	630	WQMWTLLPGFCTRPTDVAISF
Q8R0N6	144	AVGGGSTMDTCKAANLYASSP
O00468	687	GEDGDCEQELCRQRGGIWDED
Q3UMB9	1031	TLNFVEHSISCKEKLNKKNKL
Q99JX3	192	SAWGGEGSLGCGIGYGYLHRI
P62746	192	RYGSQNGCINCCKVLXXXXXX
Q9D7B6	114	SVIFEALATGCTSTTAYISIH
P13439	222	NGLPPPEKKACKELSFGARAE
Q9WU79	522	IGLHPADGQVCFGQLLGMCDQ
Q64514	1138	KQKSTLIDALCRKGCALADHL
Q8BH86	529	VSNWGGYALACALYILNSCQV
P61202	276	DESGSPRRTTCLKYLVLANML
Q3UKJ7	448	GKREGGDFVCCALSPRGEWIY
Q9R1V6	182	ENEKSQESSHCHSVYKSRQFE
P35505	288	KQDPKPLPYLCHSQPYTFDIN
Q8BGY2	152	SEEYAVAIKPCKXXXXXXXXX
Q7TQ48	863	LSQQCSYMGGCFLEKIERAIT
O15523	173	DIPVEATGSNCPPHIENFSDI
Q7L8L6	191	LLGSTSFALLCQLSVKKIQLF
P49327	1141	RAALQEELQLCKGLVQALQTK
Q13155	291	LWSVLQQIGGCSVTVPANVQR
Q8CIB5	638	FADEVRLSFICTEVDCKVVHE
O54931	64	QYLDEVLEANCCDSSVDGTYN
Q14003	355	EPFLTYVEGVCVVWFTFEFLM
P26638	60	ADNLNKLKNLCSKTIGEKMKK
Q4VXU2	508	TPGRPLLPCKCSSAAHSTYRV
Q99JX4	207	ASQARVDAHRCIVRALKDPNA
Q61656	234	TPGRLIDFLECGKTNLRRTTY
Q8BP47	557	VCLYPRFLQRCRPXXXXXXXX
Q8BTM8	1260	EPAVDTSGVQCYGPGIEGQGV
Q9WU78	127	GYEKSCVLFNCAALASQIAAE
Q6PDN3	1361	DKPDPPAGTPCASDIRSSSLT
P43897	64	RRKTGYSFVNCKKALETCGGD
Q6NXE6	5	XXXXXXMSERCCSRYSSGASI
Q8CC88	1100	EARFLSFTEECTSWKFPLDEV
P17405	588	HPPSEPCGTPCRLATLCAQLS
A2ASS6	27465	IAGIGKCSKACEPVPARDPCD
P68366	305	CFEPANQMVKCDPRHGKYMAC
Q9R0H0	396	NAGIEECRMACGGHGYSHSSG
Q99LD8	285	LCLVLSTRPHCXXXXXXXXXX

P19096	1010	YDYGPQFQGICEATLEGEQGK
P70388	990	LNGVAVQLNECEKHREKINKD
A2ASS6	5446	IEVKHDGKYVCQAKNDAGIQR
Q8BFZ1	189	PVVHLAFFCHCIHYIRLLLET
P17844	354	TIVFVETKRRCDELTRKMRRD
P68366	200	TTHTTLEHSDCAFMVDNEAIY
O70572	83	FRSGMIGSGLCVFSKHPIQEI
P35557	213	VNDTVATMISCYYEDHQCEVG
Q62407	810	TATNELGQATCASSLAVRPGG
Q01320	426	FKAQIQLNKKCSAVKHTKIKG
Q5VYK3	449	ALVQQLFEALCKEEPETRLAI
Q86VP6	356	SWKVRRAAAKCLDAVVSTRHE
Q64511	375	VKNHIWVFINCLIENPTFDSQ
A2ASS6	7650	ATLGASVVLECRVSGSAPISV
Q69Z37	1444	RSFRRQYKHMCRSRQPSTLFY
Q9EPL8	151	NSACWLGILLCLYQLVKNYEY
P97447	252	GQSWHDYCFHCKKCSVNLANK
Q8BVE3	204	SSQYVQCVAGCLQLMLRVNEY
Q8BP48	143	SEDIEGMRLVCRLAREVLDIA
Q8VBW6	294	SIEDIFNDDRCINITKQTPTF
Q9NYY3	665	FRLTTLLMSGCSSELKNRMEY
P62879	218	IKLWDVRDSMCRQTFIGHESD
P53569	560	RKMLDPGLTTCSKQAMFLNLI
P48200	467	VTDMKSDFQACLNEKVGFKGF
P06213	243	LCCHSECLGNCSQPDDPTKCV
Q5U458	38	EELKAAYRRLCMLYHPDKHRD
Q9BUJ2	337	DVIGCFADFECGNDVELSFTK
Q9Z2Z6	89	IIGVTPMFAVCFFGFGLGKKL
O14920	524	WREMEQAVELCGRENEVKLLV
Q8VDC0	278	QAHWIGDCVGCHLDFTLKVDG
P20073	363	GEGRLGTDESCFNMILATRSF
P98156	303	GSRQCNGIRDCVDGSDEVNCK
P14618	31	AMADTFLEHMCRLDIDSPPIT
Q6ZPY7	835	VRGVREMCDVCETTLFNIHWV
P50247	228	VAGYGDVGKGCAQALRGFGAR
Q9NYY8	217	MFSHPAFNQLCEHMMREAKIM
O60502	618	RSRAAKFEEMCGLVMGMFTRL
A2ASS6	18958	WRRANHTPESCPETKYKVTGL
O75153	588	PVPGEELPEECARAGFPRAHR
Q6PDN3	1099	EVKEVKNDVNCKKGQVGATGN
O94927	446	VAPQSRELLRCLEEEVRHLPH
P30416	202	VGEGESLDLPCGLEEAIQRME
Q00987	308	SLADYWKCTSCNEMNPPLPSH
Q9BQG0	968	HMPTGPQAASCLDLNLVTRVY
Q3ULJ0	331	FPLFTAVYQICYEGRPVTQML
Q04206	95	PHPHELVGKDCRDGFYEAELC
P70398	2019	YFQFMKKLLTCNGVYLNPPPG
Q99JW4	38	GELYHEQCFVCAQCFQQFPEG
P21817	4646	STGYMEPALRCLSLLHTLVAF
Q8K4Q0	86	VKTTPCARLECWIDPLSMGPQ
Q6ZWY3	40	SYFMDVKCPGCYKITTVFSHA
P28663	241	MFPAFTDSRECKLLKKLLEAH

Q4G0N4	193	TDPERSEGHLCLPVRYTHSFP
Q00839	453	PLFPHVLCHNCAVEFNFGQKE
P52825	324	TLRKVDSAVFCLCLDDFPMKD
Q3UMB9	677	ILNEHLLDKLCKEIEKDLRLS
Q9CR68	217	KPEWVILIGVCTHLGCVPIAN
P97443	335	YHEVVKLCRECLEKQEPVFAD
O14980	859	FLLLQAVNSHCFPAFLAIPPT
P42704	361	EDVALQILLACPVSKEDGPSV
Q8K1X1	999	KRSTYDHTRKCTDQLLLLGQT
Q9JLV5	314	SRVPNGLKTMCECMSCYLREQ
Q923E4	372	SCLICKYKVDCEAVRGDIFNQ
P06213	264	ACRNFYLDGRCVETCPPPYYH
O35678	208	PLVCRAGLKVCFGIQLLNAVA
Q6R5N8	74	PVAETYGFNKCTQYEFDIHHV
Q8BUV3	709	KLDPRPEYHRCILTWHHQEPL
P55769	102	ACGVSRPVIACSVTIKEGSQL
P14174	60	FGGSSEPCALCSLHSIGKIGG
Q06203	426	RVASPPIKYPCFMGINIPTKE
O70433	191	WHKECFVCTACKKQLSGQRFT
P18031	231	RSGTFCLADTCLLLMDKRKDP
Q99K43	72	RERLLKSISICQKELSTLCSE
O70546	1153	TPGHQENNNFCSVNINIGPGD
Q9QUR6	352	ACVRSNFLVLCYLHDVKNILQ
Q924M7	289	LKGDCVECMACSDNTVRAGLT
P12382	522	GRYEELCIVMCVIPATISNNV
P46471	98	QSEQPLQVARCTKIINADSED
P19367	834	GVVSRRAAQLCGAGMAAVVDK
Q60714	341	CTVVQYIGEICRYLLRQPVRD
P26639	237	LAMFKYNKFKCRILNEKVNTP
Q9JIK5	611	GRAGRTGVCICFYQNKEEYQL
Q9Z1Z0	779	SGMSEQASATCPPRDPEQVAE
Q8R127	361	VEKNKPNIRICTQVKGPEAGY
Q8BKC5	750	EYLTQMWHFMCDALIKAIGTE
Q6R5N8	406	PSLQKLNLNKCQLSFINNRTW
Q9D7H3	153	IAEKFGFTFNCDIKTRGYYPK
Q8BK08	180	NTIALAALVYCVKKVYELYAV
Q7L5N1	283	DKFKTDFYDQCNDVGLMAYLG
O75694	1208	LAECKLAIIHCAGYSDPILVQ
Q8BKC5	180	LDVIKRMLVQCMQDQEHPSIR
Q9DBF1	9	XXMWRVPRRLCVQSVKTSKLS
A2ASS6	17066	FRVIAKNKFGCGPPVEIGPIL
Q8TEX9	250	TPYLSEVLTFCLEVARNVALG
Q9Y3I1	171	TGFYPSEPMLCSESVEGQVPH
Q99832	370	FFTGCPKAKTCTFILRGGAEQ
P23242	192	SAVYTCKRDPCPHQVDCFLSR
P21817	2565	CLAVLPLITKCAPLFAGTEHR
Q8VE38	19	GLSRGSVGAVCTQAASWGLKA
P53569	443	ISPKAQYYAICFLNQMVLSHE
P70398	577	IPALKQIREICSLFGEAPQNL
Q68FD5	934	GQCDLELINVCNENSLFKSLS
Q9WTI7	441	NSFEQFCINYCNEKLQQLFIE
Q16543	203	WCIDLEVEEKCALMEQVAHQT

P39053	607	YKDYRQLELACETQEEVDSWK
O00468	578	GSDGHTYPSECMLHVHACTHQ
Q9Y277	229	FGIAAKYMLDCRTSLSAKVNN
A2ASS6	5864	VVKDSDVELECEVMGTTPFEV
P21817	3170	DVQVSCYRTLCSIYSLGTTKN
Q9R1V6	431	HDFLNSGGGACLFNKPSKLLD
Q62312	146	FTLEDAASPKCVMKEKKRAGE
Q6PDN3	683	VFPEDTGTYTCEAWNSAGEVR
Q8CIB5	520	DQITTDVNPECLVSPRYLKKY
Q9BXJ9	102	SDKKYDEAIKCYRNALKWDKD
Q99NB9	795	KKIVLKVVKQCCGTDGVEANY
Q8VCW8	49	VRALSSGMVNCTNPLPIGGLS
Q8BMP6	460	EEEEEEENVTCEEKAKKNANK
Q9CQQ7	16	VLSAAATAAPCLKNAAALGPG
Q8VDK1	255	SQCYVIAAAQCGRHHETRASY
P97443	332	EGLYHEVVKLCRECLEKQEPV
Q8R5G7	430	EQAFSLGIGICFIELQGCSVR
A2ASS6	14679	IIKDCRLEDECEYACGVEDRK
Q99683	835	TSKRLAGINPCTETFTGTLQY
Q69Z37	1408	HSYPDPYFLACLLFWPENKEL
O00220	164	YTNASNNLFACLPCTACKSDE
Q8TAT6	399	NQCMALVRDECLLPCKDAPEL
P45983	213	KENVDLWSVGCIMGEMVCHKI
P34932	380	EAVTRGCALQCAILSPAFKVR
P98170	465	DRNIAIVFVPCGHLVTCKQCA
Q9CQ48	99	LTKTKRDAANCWTSLLESEYA
O70433	275	TERDDILCPDCGKDIXXXXXX
Q9DCM0	139	TRASPGHTPGCVTFVLNDQSM
Q8VBW6	384	SISEKELKLLCSNSAFLRVVR
Q8R143	78	VRKILPPASLCKLSSARWGVC
Q60759	75	KLIRDTFRNYCQERLMSRILL
P62932	609	SQVSVLMRNICATLLQERGMV
P06151	35	VVGVGAVGMACAISILMKDLA
Q8R5K2	86	LTTLRVWCYACSKEVFLDRKL
Q8CG76	164	STPVEETLRACHQLHQEGKFV
Q92616	2469	EEELSAVLQQCLLADVSGIDW
Q91YE3	185	IVPCMNKHGICVVDDFLGRET
Q9JKS4	547	RFPASSRTPLCGHCNNVIRGP
O70305	114	LAPAATPARACPPGVRASPPR
Q9CQR2	17	EFVDLYVPRKCSASNRIIAAK
Q9D7B6	249	QPTRAVIFEDCAVPVANRIGT
P14618	165	NILWLDYKNICKVVEVGSKIY
A2ASS6	4511	LTVQDRGIYSCKASNKFGADI
P47809	172	FYGALFREGDCWICMELMSTS
Q9Y2Z0	49	EQKPDDAQYYCQRAYCHILLG
Q8K298	328	SPLKTEARKPCEKPTLSQGAQ
Q3UH68	55	RTGLENGILLCELLNAIKPGL
P50171	90	ARRLLEEVQACFSRPPSVVVS
Q8R3F5	359	GQQLGSILKCCNRQAWKSYSH
A2ASS6	7135	TDLSHSGQYSCSASNPLGTAS
Q3TL44	779	DLRDLLLHDQCQITTLRLSNN
A2ASS6	13083	VTVENANNLECSSCVKVVEII

P27659	336	VTNDFIMLKGCVVGTKKRVLT
Q8CGY6	857	TQWLEILQRLCLHDQLSVQHR
P98156	239	CGRQPVIHTKCPTSEIQCGSG
Q9D7I5	180	GGYMKALEYACGIKAEVVGKP
Q8TAT6	403	ALVRDECLLPCKDAPELGYAK
Q8VDC0	503	PLATALEWVNCSCPRCKGSAK
Q8JZN7	234	QALEDVKRVVCKNVSGGVQND
O14983	377	FIIDKVDGDICLLNEFSITGS
Q91YP0	18	VGGVCGLARYCVAGGFLRASG
Q99L47	16	VSELRAFVKMCRQDPSVLHTE
Q6NXE6	127	FCDQCKQDKACRFLAAQKGAY
Q9UBF2	516	LPSILVLLQRCMMDTDDEVRD
P27635	80	ICANKYMVKSCGKDGFHIRVR
Q8BKT7	147	VMHLQKEITKCLEFKSKHEEI
P21981	105	QQDNVLSLQLCTPANAPIGLY
P00750	430	AQESSVVRTVCLPPADLQLPD
Q6PAR5	1109	DVKKKLRLALCSADSVAFPVL
Q9CR68	222	ILIGVCTHLGCVPIANAGDFG
P11983	147	IINTDELGRDCLINAAKTSMS
Q9WU65	293	CFLLCNTGQKCVFSEHGLLTT
Q00987	449	VICQGRPKNGCIVHGKTGHLM
Q62159	190	QVRKNKRRRGCPILXXXXXXX
P30101	92	KVDCTANTNTCNKYGVSGYPT
Q62159	20	IVGDGACGKTCLLIVFSKDQF
Q9JIX8	918	DGTHDKGLKICRTVTQVVPAE
P78347	903	PYRKITINPGCVVVDGMPPGV
Q09666	567	LGSPSGKTGTCRISMSEVDLN
Q13347	120	FSTDKQMGYQCFVSFFDLRDP
Q8VDC0	505	ATALEWVNCSCPRCKGSAKRE
Q6P1X6	130	ADHGPPRLSYCGGGEALAVPF
Q9D6Y9	350	SNIRWWLEEYCFDGFRFDGVT
Q9H9A6	54	SGRNLSEVPQCVWRINVDIPE
Q99700	556	VNGGVPWPSPCPSPSSRPPSR
P53621	580	VTRVKGNNVYCLDRECRPRVL
P05063	73	LFSADDRVKKCIGGVIFFHET
P23975	131	REGAATVWKICPFFKGVGYAV
P70695	117	AQERRGKYVVCFDPLDGSSNI
P02730	479	LVFEEAFFSFCETNGLEYIVG
P98156	278	SDEVNCPSRTCRPDQFECEDG
P98156	290	PDQFECEDGSCIHGSRQCNGI
O70370	147	EVKYQGSCGACWAFSAVGALE
Q64514	28	KKETGASSFLCRYPEYDGRGV
P48507	114	SSTRSAVDMACSVLGVAQLDS
A2ASS6	4416	TQVVDCGEYTCKASNEYGSVS
P35282	124	ELRKMLGNEICLCIVGNKIDL
Q8C7B8	463	IWLGEPFPTACARVDTLRAHG
P07742	444	EYTSKDEVAVCNLASLALNMY
Q09161	616	DKMIRTQIVDCAAVANWIFSS
P15170	407	IVIIEHKSIICPGYNAVLHIH
P41216	168	RPEWVIVEQGCFSYSMVVVPL
Q16555	248	RAITIANQTNCPLYITKVMSK
P30044	7	XXXXMGLAGVCALRRSAGYIL

Q8BH61	410	PQENSDGMYRCGPASVQAVKH
Q00987	438	SSLPLNAIEPCVICQGRPKNG
P53621	127	IRVWNWQSRTCVCVLTGHNHY
Q3U5Q7	76	LGPPGRSYALCVPLAPGEGCG
Q8BRK8	200	YAGPEVDIWSCGVILYALLCG
P55072	184	CIVAPDTVIHCEGEPIKREDE
P04083	263	DLELKGDIEKCLTAIVKCATS
P35557	364	TLGLRPSTTDCDIVRRACESV
Q8K2M0	252	SNRVAEGQETCPYLPPFPARG
Q9Y277	122	GKLKASYKRDCFSVGSNVDID
Q9DBB8	86	HPQHKPAVLLCLAAGKAVLCE
Q9Z1Z0	232	NSDGGIVVEDCLILLQNLLKT
Q9DBF1	330	FAAVGTAGQRCTTVRRLFLHE
Q7TPV4	683	GHICPHLTPRCLQLILAVLSP
Q921S7	188	PLIVDSLIQLCKSQILKHPSL
Q8K0S0	227	STNLYFADFYCMYTAYHYAIL
P98192	583	KPFVESYQLLCRYLLHEEDYF
Q8C7B8	338	LWDELGALWVCVILSPHCKPD
Q9CZC8	188	ERITEGVRCICNHLSLATKLD
Q9WVQ5	212	TWEKAKTMCECYDYLFDIAVS
Q9JHR7	573	QDDKFFLPKACLNFEFFSPFA
P21817	3733	MAYADIMAKSCHLEEGGENGE
Q14315	665	IAHILPAPPDCFPDKVKAFGP
Q8BP48	194	LNYYNFPKSCCTSVNEVICHG
Q9JLJ2	173	ACWKSAPALACGNAMIFKPSP
Q9BY44	42	PRESGKNCKVCIFSKDGTLFA
Q13347	99	LLKTNSAVRTCGFDFGGNIIM
A2ASS6	6988	ASVGDSVSLQCQVAGTPEITV
O89103	428	DSTQCEDIDECSDARGNPCDS
Q6ZPY7	854	WVCRKCGFGVCLDCYRLRKSR
P21817	1268	RVDGTVDTPPCLRLTHRTWGS
Q9WU78	231	ADYFGDAFKQCQYKDTLPKEV
Q16543	57	KEELDRGCRECKRKVAECQRK
P21817	1723	LLISIHLESACRSRRSMLSEY
P97494	257	NCCLQVTFQACSISEARYLYD
P28663	164	GEESNSSANKCLLKVAAYAAQ
O75369	1375	VEGPSESKINCRDNKDGSCSA
Q99KC8	464	ALGSLKFALQCAVDNISLSWD
P23975	240	IGLPQWQLLLCLMVVVIVLYF
P00558	316	GIPAGWMGLDCGPESSKKYAE
O89103	449	LCFNTDGSFRCGCPPGWELAP
Q60932	2	XXXXXXXXXMCSFFLVLLLWQ
P13804	159	YAGNALCTVKCDEKVKVFSVR
Q15149	4574	DVGAYSKYLTCPKTKLKISYK
Q64436	822	GCITILFIELCTDIFPSVSLA
Q60597	825	ENFDINQLYDCNWIVVNCSTP
Q3UK37	76	WTDLAPEAQACVVKSALGSQG
P54819	232	SILAAFSKATCKDLVMFIXXX
Q8BHP7	52	QVRMTSEGSDCRCKCIMRPLS
P28474	195	NTAKVEPGSTCAVFGLGGVGL
Q9R1V6	492	QCSDGLCCKKCKFQPLGTVCR
Q64277	273	MCVDHSTHPDCIMNSASASMR

Q60749	238	HVFIEVFGPPCEAYALMAHAM
P22315	233	RWPTHPLLIQCFADHILKELN
Q8BG05	94	SCVEEVDAAMCARPHKVDGRV
A2A432	156	DSASPSTSSFCLGVPVATSSH
P21817	1216	DVSSLRFFAICGLQEGFEPFA
Q8BH59	130	IHHHIPFNWDCEFIRLHFGHN
Q9EPB5	113	FGGCVGGTFACMFPEMVDKLI
Q99K01	202	NQLGLPFPCLCRVPCNTMFGS
Q60605	2	XXXXXXXXXMCDFTEDQTAEF
Q8CG76	190	NYASWEVAEICTLCKSNGWIL
P98156	734	QINDHSPKYTCSCPNGYNLEE
Q6PDN3	1203	VAKNSAGQAECSCQVTVDDAQ
Q13868	191	RQKTHFHDLPCGASVILGNNG
P40227	499	AAEVGVWDNYCVKKQLLHSCT
Q92769	312	GGYTIRNVARCWTYETAVALD
P07744	490	GSGLGLGSGFCSGSGSGSGFG
P07356	223	WISIMTERSVCHLQKVFERYK
Q8R5G7	225	ASDRRDGRGVCQERAEHRQDL
Q8NBI5	480	FFHPFLVYRECRTWKESPSAI
Q9DAW6	430	HIATGSGDNTCKVWDLRQRRC
Q9BXP5	421	ECKPRPLHKTCSLFMRNIAPN
P53996	123	DHADEQKCYSCGEFGHIQKDC
P63318	85	RRCHEFVTFECPGAGKGPQTD
Q14690	361	QPGRPLTRLSCQNLGAVLDDV
P31327	1256	VKGNDVLVIECNLRASRSFPF
O35643	391	AIKVEQSAERCVSTLLDLIQT
A2ASS6	30687	VKENKVPCLECNYKVTGLVEG
Q6IFX4	333	AQHRMRESQECILTETEARYT
Q91ZA3	286	GNALWLNERECSIQRRNQKVV
A2ASS6	24000	NNSLLTIKEACREDVGHYTVK
P61620	148	GDPSEMGAGICLLITIQLFVA
P70398	1323	QTFIIDLLLHCHSKTVRQVAQ
Q99683	666	EKGRSTEEGDCESDLLEYDYE
P63318	114	RLHSYSSPTFCDHCGSLLYGL
Q921G7	585	GFRLQINAQNCVHCKTCDIKD
P16546	158	AQKLVQYLRECEDVMDWINDK
P54729	51	LAKKYSERLECCENEVENIIE
Q9EPL8	113	PELIRVQLTTCIHHIIKHDYP
O00468	502	QACSSLYDPVCGSDGVTYGSA
Q5SFM8	21	SWLAKLLEPICDADPSALANY
Q9BSK1	163	AGGKPHECSVCGRAFSRKAQL
Q5SSW2	631	RVAGRMVADMCRAAVKCCPEE
P01111	80	QYMRTGEGFLCVFAINNSKSF
Q61733	21	PRPFSGLPLSCGNRDVSVAVL
Q3UH68	182	RSIRDSGYIDCWDSERSDSLS
P35557	220	MISCYYEDHQCEVGMIVGTGC
Q9WU65	234	EIPMSILPNVCSSSEIYGLMT
Q9WTP6	92	LIEKNLETPSCKNGFLLDGFP
P16546	1454	CLELQLFHRDCEQAENWMAAR
Q00610	1565	QWFLQEEKRECFGACLFTCYD
Q9ER72	418	ASKPGEPSWPCPWGKGRPGWH
Q71FD7	245	EPCYQDTLEKCGKKCGEVVQEH

Q810L3	61	PSNKLVSGDHCKLTVDEISGE
Q6A0A9	14	QGFQDYIEKHCPSAVVPVELQ
Q60714	326	KFSASRFWDDCVKYNCTVVQY
Q9JLJ2	381	YYMTPCILTNCRDDMTCVKEE
Q8C0M9	179	KLEKGAQNADCPKNSGTVGAV
Q8K4Q0	594	IWQNFDSARWCGVRDSAHEKL
Q9ET26	33	DPLSRFTCPVCLEVFEKPVQV
Q99KN2	97	IWKKNQDDFECVTTLEGHENE
P62932	95	LQVCPASVVCCSMEWIRWPNV
O00468	1105	TPGPPVERASCYNSALGCCSD
Q8VBZ3	145	DWTSGENSDGCYEHFAELDIP
Q69Z37	755	VLWDLKQKFRCAVLKNKATDF
P20073	285	TQERVLIEILCTRTNQEIREI
P54071	12	AGYLRAVSSLCRASGSARTWA
Q6PDN3	571	ALPEDRGTYTCLAENAMGQVS
O88712	118	KSAGDLGIAVCNVPAASVEET
P53569	472	ITLYFCFFRTCIKKKDIESKM
P26039	1978	LQAGNRGTQACITAASAVSGI
Q8VDK1	288	VARCSEGPGLCLARIDLHFLQ
O08739	273	LITDGPTKTYCHRRLNFLESK
P21817	4113	SGPEIQFLLSCSEADENEMIN
Q9H4A6	84	REGYTSFWNDCISSGLRGCML
Q9ER60	182	SLIKMLARGFCIDDFTFLRDP
P78527	1399	VQVMAHLPDVCVNLMKALKMS
Q8VCI5	128	DASSQQEFTSCLKETLSGLAK
Q99KB8	9	XXMVLGRGSLCLRSLSALGAT
Q8BP47	353	LAEFTHVEAECPFLTFEDLLN
Q8K298	975	LEGHICLKISCQVNSAVEEKG
A2ASS6	32738	VRYQSNATLVCKVTGHPKPIV
P78527	285	FALHASQFSTCLLDNYVSLFE
Q9NP79	38	DKRDPVVAYYCRLYAMQTGMK
Q6IFX4	54	CALQTPQGQGCGSSPCLYRCP
Q9D6Y7	218	QYLSKNPDGYCGLGGTGVSCP
Q99683	185	FSMANNIILYCDTNSDSLQSL
P98156	367	INECLVNNGGCSHICKDLVIG
P51787	136	HFAVFLIVLVCLIFSVLSTIE
Q05586	932	IEREEGQLQLCSRHRESXXXX
O00468	602	HVASAGPCETCGDAVCAFGAV
P12004	62	LRSEGFDTYRCDRNLAMGVNL
Q61768	330	QRAKTIKNTVCVNVELTAEQW
P53569	603	VTCTQMPPFICGALYLVSEIL
A6H611	140	PAFREAAQEACRSIGTMVEKL
Q9Z2I8	370	LVNIFGGIVNCAIIANGITKA
Q8BGD5	609	ETVRSCTREACQFVRAMDNKE
P49368	279	QMEEEYIQQLCEDIIQLKPDV
A2ASS6	3709	KAQNLWGESTCAAELLVLPED
A2ASS6	31871	QKVDQREWTKCNTTPTKIREY
P70398	819	VVIHEDFIQSCFDRLKASYDT
P60981	12	ASGVQVADEVCRIFYDMKVRK
Q8BK64	207	AKPVGVKIPTCKITLKETFLT
P28271	370	ELDLKTVVPCCSGPKRPQDKV
A2ASS6	5926	IIANEGGSCACSARVALKEPP

P53041	343	EVFEWLPLAQCINGKVLIMHG
A2ASS6	26786	PSEPSVFYRACDALYPPGPPS
Q8BFR6	217	NKLTAKKLRLCHVPSGEALPL
A2ASS6	5271	EAQNEAGSDHCTGIVIVKESP
Q8BFR6	98	LRHRHQSDHDCEKLEVAKPRM
P28271	392	VSEMKKDFESCLGAKQGFKGF
P98156	200	PPTCGAHEFQCSTSSCIPLSW
Q9Z1Z2	270	SYKGHFGPIHCVRFSPDGELY
O89103	436	DECSDARGNPCDSLCFNTDGS
Q9WUM3	79	GRIDKAYPTVCGHTGPVLDID
Q9D710	59	LPTQREDGNPCDFDWREVEIL
O94760	275	LEKVDGLLTCCSVLINKKVDS
Q8CG76	193	SWEVAEICTLCKSNGWILPTV
P10242	347	RPHGDSAPVSCLGEHHSTPSL
P14866	521	EVTEENFFEICDELGVKRPSS
Q9CQS4	326	ELIANFAASLCSDVILYPLET
O94927	192	PVVLRDVRTACTLRAQFLQNL
P21817	2310	SYLAGCGLQSCPMLVAKGYPD
P99029	11	MLQLGLRVLGCKASSVLRAST
Q92600	252	NPRAREALRQCLPDQLKDTTF
Q8N0X7	123	LYPEFPPKDMCEKLPEPQSFS
Q61792	5	XXXXXXMNPNCARCGKIVYPT
Q99798	332	FKDHLVPDPGCHYDQLIEINL
Q9CXJ4	347	ERYQAELESCCCKAEELGRGI
Q91YE3	33	LCGKMENLLRCGRCRSSFYCC
Q5VYK3	1347	SSPMMETINMCLQYLDVSVLG
Q71FD7	274	FHPPCFTCVTCARCISDESFA
Q99L13	198	LECMGSNVVYCGAVGTGQSAK
Q5UIP0	2223	AGLADDIDRRCSIVRSHSSNS
Q99K43	14	SEVLADESITCLQKALTHLRE
P42704	413	HSFPLQFTLHCALLANKTDLA
Q8BG51	229	DAELNFFQRICFNTPLAPQAL
Q9BQ04	107	KFEEYGPVIECDIVKDYAFVH
Q99JX3	173	LYVYNTDTDNCREVIITPNSA
Q8WX93	703	PPPAFPELAACTPPASPEPMS
Q8BKY8	15	LPTGHQLCRLCLLRKPRPALK
Q9ES28	315	QQVLVQSLEECTKSPEAQQRV
Q60770	402	PVLLNKNHDNCDKIRAVLLYI
Q921M4	762	RVQLKEQKARCRSLSHLAAPV
A6H611	560	PLPKAMVSRLCESKKVCTAAE
Q91WD7	153	CIDEIKEEKECSTAVSYLEVY
O70325	102	ECGLRILAFPCNQFGRQEPGS
P60709	374	ESGPSIVHRKCFXXXXXXXXX
Q8BY71	117	KIRQIIPPGFCTNTNDFLSLL
Q8R5G7	502	SNPANRHCADCRASRPDWAAV
Q62407	1469	VSRRDLGPLTCSARNRHGTKA
P62911	91	NVKELEVLLMCNKSYCAEIAH
P48643	302	IKETGANLAICQWGFDDEANH
Q91ZA3	72	ILIANRGEIACRVIKTCKKMG
Q9EPL8	722	TKYLEMIYSMCKKVLTGVAGE
Q9WV35	174	AALKKLKEAGCKLRIMKPQDF
Q8BGQ7	184	NFWEMGDTGPCGPCSEIHYDR

Q8VBT1	585	CEATPAPTASCTPAEAELQSQ
Q9JIK5	363	SDITKKLSVACFYGGTPYGGQ
A2ASS6	16272	NVKVIGLPGPCKDIKASDITK
Q99JB8	42	GHRLCGDLVSCFQERARIEKA
Q5UIP0	766	IIYIITVMVDCIDFSPYNIKY
A2ASS6	32552	SWITNYVVEKCEAKEGAEWQL
P30046	57	AMALSGSTEPCAQLSISSIGV
P26639	206	SNDFSSLEALCKKIIKEKQAF
A2ASS6	2294	VPESYSGELECIISPENIEGK
P37268	258	KPENIDLAVQCLNELITNALH
P41216	487	TAGHVGAPMPCNYVKLVDVEE
Q5UIP0	917	ELLEQLSPLLCIIFLHKNKQI
P35821	339	SSHQWVSEETCGDEDSLAREE
P19096	879	ESPDHYLVDHCIDGRVIFPGT
Q9CR24	39	PAAAAVLVPLCLVRGVPALLY
Q3ULD5	13	GALRSALRPCCRAAVPPQRAY
P31948	420	FQLALKDCEECIQLEPTFIKG
Q08J23	267	KEILFYDRILCDVPCSGDGTM
Q3U1J4	18	TAQKPTAVNGCVTGHFTSAED
Q64337	219	RPPRAGDGRPCPTAESASAPP
Q9NX47	17	QQMLDRSCWVCFATDEDDRTA
Q99832	158	KVEQRKLLEKCAMTALSSKLI
Q91WD5	9	XXMAALRALRCLRGVGAPVLR
Q60973	277	LVDAHTAEVNCLSFNPYSEFI
P14780	347	VMGGNSAGELCVFPFTFLGKE
Q14166	254	YGETDPLIRKCMLLPWAPTDM
Q99832	29	IPQLVSNISACQVIAEAVRTT
Q6ZPY7	800	YILANVGDQFCQLVMSEKEAM
P19367	886	HQTVKELSPKCNVSFLLSEDG
P21817	3216	LEPQLNEYNACSVYTTKSPRE
Q9NYY3	482	ADTVARVLRGCLENMPEADCI
P06213	223	SHCQKVCPTICKSHGCTAEGL
Q8R5G7	1251	QERFFLVRGRCLLLLKEKKSS
Q8CGY6	487	KIKIRTLVGLCKLGSAGGSDY
P26039	1353	TDSINQLITMCTQQAPGQKEC
Q8BVF2	155	VKFIKAISTTCIPNYPDRNLP
Q9C0B1	338	TGTLDYILQRCQLALQNVCDD
Q9ET26	174	THSTDTKSVVCPICASMPWGD
Q3LXA3	24	ADDALAGLVACNPNLQLLQGH
Q9WUB4	128	ILTSGCIIGACCSLNTFEAIP
P61080	107	LTVSKVLLSICSLLCDPNPDD
Q8BMF3	19	ARLTSVPRIACSSLRRQAPSA
Q8K0G5	251	QYYLASCGDDCKVKFWDTRNV
P19367	133	GSQLFDHVAECLGDFMEKRKI
O70546	1334	KEEPAHYCSICEVEVFDLLFV
P56399	727	SAAADPPPEDCVTTIVSMGFS
Q91V76	226	AHIMPAEFSSCPLNSDEAVNK
P40936	116	YDWSSIVQHACELEGDRSRWQ
Q60714	103	LALVDASSGICWTFAQLDTYS
Q64514	372	NNGPCLSTVGCPGGTTSSVIG
P53621	140	VLTGHNHYVMCAQFHPTEDLV
O43447	122	MANSGPSTNGCQFFITCSKCD

Q8K4Q0	1270	DIHPQANLIACGSMNQFTAIY
Q08211	1004	LLAFGVYPNVCYHKEKRKILT
P53041	11	MAMAEGERTECAEPPRDEPPA
Q91WU5	369	PRHAPEGTGGCCGKRKNCXXX
Q6PEB6	92	NGLAVKLQSECHPDTCTQMTA
Q99KP6	298	IRIWSVPNTSCVQVVRAHESA
O89103	314	SSNPCTGGGMCHSVPLSENYT
Q99L13	74	LILYDVFPDVCKEFKEAGEQV
Q9DBB5	85	IPPVTSLPLRCSYHLMRGERR
P78527	2403	LKTLCLEVVLCRVEGMTELYF
Q15527	83	PSTKNPHQLFCKLTLRHINKC
P78527	373	IRGYGLFAGPCKVINAKDVDF
P00533	207	GSCWGAGEENCQKLTKIICAQ
A2ASS6	5353	FVAADAGEYQCRVTNEVGSST
Q91VI7	243	KLGNAGIAALCPGLLLPSCKL
Q9JIX8	676	TSYTETKDPSCGQEAAAPSGP
Q9Z2W0	267	FAPRLDNLHSCFCALQALIDS
P17812	299	ADRYDRLLETCSIALVGKYTK
A2ASS6	3670	KEDMPLYTSVCYTIIHSPDGS
Q8BMF4	33	AALKEGPGAPCGSPRIGPAAV
P62820	26	LIGDSGVGKSCLLLRFADDTY
A2ASS6	6609	GSMTVTVGETCALECKVAGTP
Q91WU5	157	SESYDIVISNCVINLVPDKQQ
Q3UMB9	477	PMTKTSVKALCRLIELLKAIE
Q8BWM0	112	TLYQYKTCPFCSKVRAFLDFH
Q96I24	460	QNTFPPRSSGCFPNMAAKVNG
O00468	1548	AATRGSGVGECGDHPCLPNPC
P63005	409	SVDQTVKVWECRXXXXXXXXX
Q6PDN3	1735	DMKNRLDCTQCLQHPWLMKDT
P21333	1198	AGSAELTIEICSEAGLPAEVY
Q12879	429	VEDIDPLTETCVRNTVPCRKF
Q9DC70	99	SLWPMTFGLACCAVEMMHMAA
Q14690	694	QAGDILHRVLCLSQSEGRVLL
P22314	23	SGPDPKPGSNCSPAQSVLSEV
O95861	59	KADRLAQMSICSSLARKFPKL
Q6PDN3	106	VREEDKGKYTCEASNGSGARQ
Q8BTM8	841	NDTFTVKYTPCGAGSYTIMVL
Q8BP48	25	SSEAKLQCPTCIKLGIQGSYF
Q9CQ65	223	ASIAMATDYDCWKEHEEAVSV
Q3TL44	259	LDFRLAGTGLCSDPEEPGPPA
Q9ET80	15	RFDFDDGGTYCGGWEEGKAHG
Q09666	5382	GDLAVSGDIKCPKVSVGAPDL
Q99K01	456	TVIDLEVDGTCVRFSPLMTAE
O00220	170	NLFACLPCTACKSDEEERSPC
Q9D883	27	NCSFYFKIGACRHGDRCSRLH
Q8K2M0	16	WRAAFFGIGRCRGFSTSAFLS
A2ASS6	5833	EARNDAGTASCSIELKVKEPP
A2ASS6	34237	TEDGGTYRAVCTNYKGEASDY
Q62261	619	IRDRVAHMEFCYQELCQLAAE
Q810L3	529	HLYWGCTRTGCFGCLAPFCEL
Q99497	106	ENRKGLIAAICAGPTALLAHE
P21981	371	PQEKSEGTYCCGPVSVRAIKE

Q9UL62	181	PHQIRCNCVECVSSSEVDSLR
P35579	118	GLIYTYSGLFCVVINPYKNLP
Q8BH95	12	AALRALLPRACSSLLSSVRCP
Q9ET26	144	RNIPNRYTFPCPYCPEKNFDQ
P18760	147	ANCYEEVKDRCTLAEKLGGSA
O95801	321	TPSWDLEQKYCPDNLEVYFED
Q9Z2X1	400	SGLESQSVSGCYGAGYSGQNS
P97822	29	VTELVLDNCLCVNGEIEGLND
Q8CI94	496	GITPRRWLLLCNPGLAEIIVE
P19838	925	IDELRDSDSVCDSGVETSFRK
P50990	36	EEAVYRNIQACKELAQTTRTA
P48444	389	NCWPSESGNGCDVNIEYELQE
P56399	199	PARIPPCGWKCSKCDMRENLW
Q64514	1260	WLPIMYPPDYCVFXXXXXXXX
Q5JTH9	763	VLDLVVALAPCADEAAISKLY
Q9Z1Z0	310	TSSCQKAMFQCGLLQQLCTIL
P50247	278	EGNIFVTTTGCVDIILGRHFE
Q13144	414	AGAQIHQSLLCDNAEVKERVT
Q9EQP2	495	GKIWKLADCDCDGMLDEEEFA
A2ASS6	27666	VAGTRTMAVNCKVLDKPGPPA
P06213	462	KLFFHYNPKLCLSEIHKMEEV
P53041	208	YKDQKKLHRKCAYQILVQVKE
Q8JZX4	334	EDLEVETKEECEKYGKVGKCV
Q99P30	72	DKLKREPGEVCFPGGKRDPVD
Q9JHK4	532	QSAALQTLASCPRLVFLNLQG
P13489	152	CRLEKLQLEYCSLSAASCEPL
Q9JHU4	1947	QAMGRIFVGLCQVGAWGCFDE
P98170	484	CAEAVDKCPMCYTVITFKQKI
Q9DCS1	159	FYWYFLGDDVCQRDSSYGWST
Q9WUM3	153	TARNVLLSAGCDNVVLIWNVG
Q92769	285	FNLTVKGHAKCVEVVKTFNLP
Q9WV60	218	VRGEPNVSYICSRYYRAPELI
Q9UL62	954	EDVFETWGEACDLLMHKWGDG
A2ASS6	17855	TGKKKEAWRQCNRRDVEELEF
P21817	746	LLAPEDVISCCLDLSVPSISF
P29474	908	RRYEEWKWFRCPTLLEVLEQF
Q3TL44	374	IAAACFLPSYCWLVCATLHFL
Q9ESD7	1645	PVFGKMFELTCTLPLEKDLKI
Q8TEX9	400	RLLPPLLQIVCKGLEDPSQVV
P02463	1551	IRPFISRCAVCEAPAMVMAVH
Q60870	55	LVFGYGASLLCNLIGFGYPAY
Q8CC88	723	SLEPELENYKCEVVAGSLKIG
Q71FD7	325	RDGKDAFKIECMGRNFHENCY
P21333	1108	GGLGLTVEGPCEAQLECLDNG
Q923E4	61	SAAVAPAAAGCEAASAAAPAA
P97447	212	AVEDQYYCVDCYKNFVAKKCA
Q91WU5	251	NKELEGVLGDCRFVSATFRLF
O95219	433	ISYAVMQISMCKKGIQVWTNA
Q9DBG3	818	KNNIDVFYFSCLIPLNVLFVE
P63037	150	RGGKKGAVECCPNCRGTGMQI
P21333	1122	LECLDNGDGTCSVSYVPTEPG
Q8R0N6	21	HLLRHLQSTACQCPTHSHTYS

O75694	844	TGALIASLINCYIRDNAAVDG
Q08211	872	PRFGKMMIMGCIFYVGDAICT
P62333	170	QRVGIIPPKGCLLYGPPGTGK
O88712	312	GPLKDAPNLICTPHAAWYSEQ
Q8VCT3	254	GPRSRVWAEPCLIEAAKEEYS
Q3UZY0	1204	LEAQRQPVGTCIARVRALRRA
Q02053	632	QDPPEKSIPICTLKNFPNAIE
Q9D1P4	162	KIGTSCKNGGCSKTYQGLQSL
O89103	164	SHLPKWHESPCGTPEAPGNSI
Q80YD1	175	HAKQIFPVLECKDDLRKISDL
Q9UBF2	440	ESKEAGLAHLCEFIEDCEHTV
Q99JW4	13	NALASATCERCKGGFAPAEKI
P54729	52	AKKYSERLECCENEVENIIEE
P62932	358	VYTFKIPVSYCGKRARLGDAM
Q99KR7	30	APLLLSATRTCSDGGARGANS
Q5SSW2	718	LTCKQGYTLSCNLLHHLLRST
Q9Z2I8	318	VNGAGLAMATCDIIFLNGGKP
P53396	1040	GVAFVDMLRNCGSFTREEADE
Q8BUN5	307	YYIGGEVFAECLSDSAIFVQS
P00533	326	YEMEEDGVRKCKKCEGPCRKV
Q9EQ20	179	LYSYRLPLGVCAGIAPFNFPA
Q3UHX9	210	TKAGHGSLVNCGMKKEVKIDK
O14920	99	NDLPLLAMEYCQGGDLRKYLN
P12268	26	DGLTAQQLFNCGDGLTYNDFL
P54886	584	IPVMGHSEGICHMYVDSEASV
Q9JHK4	340	ECVLLKGHQECWCRDSATDEQ
Q9EPL8	144	GFYLQSDNSACWLGILLCLYQ
A2ASS6	29497	EASQTNWKMVCSSVARTTFKV
Q99L04	63	SLGGRCVPVVCDSSQESEVKS
O95573	652	KGTWEELCNSCEMENEVLKVL
Q9WTI7	480	PVQYFNNKIICDLVEEKFKGI
P10768	158	HSMGGHGALICALKNPGKYKS
Q61768	858	QLVRDNADLRCELPKLEKRLR
P70398	1512	FELLVALAVGCVRNLKQIVDS
A2ASS6	13828	GVEKDEVILQCEISKADAPVK
Q9C0B1	136	KHTEAEIAAACETFLKLNDYL
Q96EK6	150	TLTLLSKKLNCYKITLECLPQ
P15880	143	YNGHVGLGVKCSKEVATAIRG
P70695	93	INMLQSSYSTCVLVSEENKEA
P05141	57	ADKQYKGIIDCVVRIPKEQGV
P31327	516	LGMGGQTALNCGVELFKRGVL
Q99700	852	DSFIENSSSNCTSGSSKPNSP
Q9Y2S7	4	XXXXXXXMAACTARRALAVGS
Q810L3	343	ERSSLCPTCRCPVERICKNHI
P23492	31	TEYRPQVAVICGSGLGGLTAH
Q8BY71	165	TFQIHKADMTCRGFREYHERL
Q9Y3I1	252	LCEGSSATLTCVPLGNLIVVN
Q9JKS4	696	WHDTCFICAVCHVNLEGQPFY
Q9D710	120	IRMGLLYLTLCIVFLMTCKPP
Q3TC72	300	VFLKKGDEVQCEIEELGVIIN
Q9CR76	92	TVVALPPGFYCYSQGLMTLSS
P26039	956	AGPQPLLVQSCKAVAEQIPLL

P26039	2408	RMVAAATNNLCEAANAAVQGH
O00170	78	FKLPVWETIVCTMREGEIAQF
Q14181	82	KRLSKARHSTCKDSGHAGARD
A2ASS6	14362	ILEGEKAEFVCTISKESFEVQ
Q62234	656	HEGIMYFVEKCDVGAENWQRV
P46060	152	FTLQELKLNNCGMGIGGGKIL
Q9UBF2	387	VVVVQAISALCQKYPRKHSVM
Q9D1E6	51	LELVVGSPASCMELELYGADD
A2ASS6	16678	AENRFGPGPPCVSKPLLAKDP
Q80UJ7	510	LASGSPDLRCCLLHQKLQMLN
P17426	397	QRAADLLYAMCDRSNAKQIVS
Q5SSW2	1715	AATTLSGLLQCNFLTMDSAMQ
Q8K0S0	17	PHSIEINNITCDSFRISWAME
Q14152	185	HDIAQQAFKFCLQYTRKAEFR
Q8BIJ6	155	SKVHFVPGWDCHGLPIETKVL
Q5JTH9	288	HPAAISTAKFCIQEIEKSGGS
P12814	180	HISWKDGLGFCALIHRHRPEL
Q14166	638	STLFLDQPGGCHVTCLVXXXX
P53618	284	APTAIKAAAQCYIDLIIKESD
P82933	353	AIRLAMAKALCSFVTEDEVEW
P00533	362	NATNIKHFKNCTSISGDLHIL
Q9WU79	435	DMELARREGWCFGAKLVRGAY
P21266	3	XXXXXXXXMSCESSMVLGYWD
P97311	721	PSLRLGFAEYCRISNLIVLHL
Q9CPZ8	53	KDSGILMVLKCRKENSALKDC
P61202	392	VADVESLLVQCILDNTIHGRI
P22315	357	IEYSQVLAQKCGAENIRRAES
Q5JTH9	125	ESNSAAHKEICAVLAAVTEVI
Q9GZT4	2	XXXXXXXXXMCAQYCISFADV
P08133	552	SLETRFMTILCTRSYPHLRRV
P97447	31	QKDGRHCCLKCFDKFCANTCV
O70572	252	YKAVSEFHVCCETLKTTTGCD
A2ASS6	6490	QNEVGSDACVCAVKLKEPPKF
Q3U5Q7	110	QQLRRGPLQRCQLSKLLGYGP
Q01433	310	PALEQHPYEHCEPSTMPGDLG
O75694	1276	FIVQFLEQQVCTLNWDVGFVI
O43175	254	ALLRALQSGQCAGAALDVFTE
P08752	112	DDARQLFALSCAAEEQGMLPE
