# Supplementary material for: iSNO-AAPair: incorporating amino acid pairwise coupling into PseAAC for predicting cysteine S-nitrosylation sites in proteins
Source: PeerJ. 2013 Oct 3;1:e171. doi: 10.7717/peerj.171 (PMC3792191; doi:10.7717/peerj.171)
Supplement: Supplemental Information S2 — The former contains 81 SNO sites and the latter 100 non-SNO sites. None of the sites and peptide fragments included here occurs in the learning dataset 𝕊L. See the text of the paper for further explanation. [file peerj-01-171-s002.pdf]

Online Supporting Information S2. The independent testing dataset $\mathbb{S}_{\mathrm{T}}$ consists of a positive dataset $\mathbb{S}_{\mathrm{T}}^{+}$ and a negative dataset $\mathbb{S}_{\mathrm{T}}^{-}$. The former contains 81 SNO sites and the latter 100 non-SNO sites. None of the sites and peptide fragments included here occurs in the learning dataset $\mathbb{S}_{\mathrm{L}}$. See the text of the paper for further explanation.

$\mathbb{S}_{\mathrm{T}}^{+}$ contains 81 SNO sites and their corresponding peptide fragments in proteins

Uniprot ID	Site	Sequences
Q08211	1029	NALIHKSSVNCPFSSQDMKYP
P97447	71	WHDNCFRCAKCLHPLASETFV
Q99NB1	41	SRSTATRLPGCVPAAAQPGSY
O08756	91	KFGRIDVAVNCAGIAVAIKTY
Q8BGK2	294	LASGSNWTELCQRAMFHGGES
Q9Z2I9	270	MVEDSDGKVLCMDAKINFDSN
O95573	450	LLGGNIRLLLCGGAPLSATTQ
P13804	53	AATRLGGEVSCLVAGTKCDKV
P46060	169	GKILAAALTECHRKSSAQGKP
Q13907	172	NPDPNEIKSYCYVSKEELKEL
O00468	405	CPEPCRFNAVCLSRRGRPRCS
O55143	998	RNYLEQPGKECVQPATKSSCS
P23297	86	VVLVAALTVACNNFFWENSXX
Q8JZX4	343	ECEKYGKVGKCVIFEIPGAPD
Q9WU79	162	EEAERKEMESCTSEAERDGSG
O60502	896	RILEFYSKLGCFEIAKMEGFP
P68366	54	GGDDSFTTFFCETGAGKHVPR
P21817	2326	KGYPDIGWNPCGGERYLDFLR
P11142	574	DEDKQKILDKCNEIINWLDKN
Q9NPA8	40	KELLRAKLIECGWKDQLKAHC
P23528	147	ANCYEEVKDRCTLAEKLGGSA
O75390	211	DSMDLIAKLPCVAAKIYRNLY
Q99439	61	QKGLKDGTILCTLMNKLQPGS
P56382	19	GLSYIRFSQICAKAVRDALKT
Q99JW4	94	KAMNNSWHPECFRCDLCQEVL
P62878	94	ISRWLKTRQVCPLDNREWEFQ
Q9UBF2	296	APAVSVLQLFCSSPKPALRYA
Q8BMS1	747	ESAYGTQFTPCQLLLDHANNS
P50914	54	QVRRQAMPFKCMQLTDFILKF
Q61425	211	CKTLGKHPVSCKDTPGFIVNR
P10518	162	EVALAYAKAGCQVVAPSDMMD
P07237	53	YLLVEFYAPWCGHCKALAPEY
P61982	112	SLLDNYLIKNCSETQYESKVF
Q9Z0X1	255	NDGSQITFEKCLIATGGTPRS
P50247	195	TKSKFDNLYGCRESLIDGIKR
P97370	249	GLNKKEVTVECHIAGTRNLKN
P63244	249	LCFSPNRYWLCAATGPSIKIW
Q9JHI5	348	RQYVYNVAKACDEGHIIPKDC
O70572	176	TSKNADVVLLCGDLNMHPKDL
Q9CQ60	33	AQLVAQRAASCLEGDRGRFAL
P51174	342	HKLAELKTHICVTRAFVDSCL

Q9WTI7	763	KFLRVKRSAICIQSWWRGTLG
P16858	154	IVSNASCTTNCLAPLAKVIHD
Q8R081	401	MNCDRVFNVFCLYGNVEKVKF
Q9D172	219	EAIKALGAKHCVKGVTEAHVD
Q80UJ7	181	HKWRRMYMGECQGPGVRTDFE
P35754	83	VFIGKDCIGGCSDLVSLQQSG
Q9R0P5	23	RIFYDMKVRKCSTPEEIKKRK
P04350	354	WIPNNVKTAVCDIPPRGLKMA
P48444	441	HDSRRNTLEWCLPVIDAKNKS
Q9CQR4	74	VDSISTMALMCTERGAPGVSV
O75369	604	IEYNDQNDGSCDVKYWPKEPG
P10415	158	VAFFEFGGVMCVESVNREMSP
A2AJB2	26	RHPGLEEFAACQSHAFMKGVF
Q924X2	86	KVDISMGLVDCIQRCLPERYG
Q9CQ48	45	PPGTRAQDIQCGLQSRHVALA
P08670	328	EYRRQVQSLTCEVDALKGTNE
P07900	597	VVSNRLVTSPCCIVTSTYGWT
P51125	408	AKAKEERQEKCGEDEDTVPAE
Q9NP79	155	ARWKATYIHNCLKNGETPQAG
Q9DBC7	18	TSEEERSLRECELYVQKHNIQ
Q91W50	129	APGQSPTGSVCYERNGEVFYL
Q06830	173	FQFTDKHGEVCPAGWKPGSDT
P53618	248	NPSERARFIRCIYNLLQSSSP
Q9D819	242	TKKTDGKGISCMNTTVSESPF
Q9D023	54	WAPIMKWGLVCAGLADMARPA
P28650	183	SKAARTGLRICDLLSDFDEFS
Q8VDG5	308	DLRSRHTAFICDKNXXXXXXX
Q9WVQ5	146	HQEMIKGIRKCTSGGYYRYDD
O09110	29	SKRKKDLRISCVSKPPVSNPT
Q8BH95	225	VEKLVEEAIQCAEKIASNSKI
P63330	20	LDQWIEQLNECKQLSESQVKS
Q8BW75	365	LTKEERLRKLCELYAKVLNSQ
P55060	344	FEDQNTLTSICEKVIVPNMEF
O95801	374	FLVCVGSSPFCKNFLRGRKVY
Q9DC70	164	EPRYVVSMGSCANGGGYYHYS
P62910	91	NVKELEVLLMCNKSYCAEIAH
Q60864	370	ALEEKNKGNECFQKGDYPQAM
P19338	543	FEDAKEALNSCNKREIEGRAI
O94874	708	THSMLHAPGRCVPQIIAFLNS
O35295	250	DSKRFFFDVGCNKYGVFLRVS

$\mathbb{S}_{T}^{-}$ contains 100 non-SNO sites and their corresponding peptide fragments in proteins

Uniprot ID	Site	Sequences
Q99KB8	20	LRSLSALGATCARRGLGQALL
Q9CXJ4	259	KLVISQGLRSCTQVIGSLVSL
Q99JW4	100	WHPECFRCDLCQEVLADIGFV
Q8K274	150	VDQFGFDVVTCCGYLPQVNDW
Q3UZY0	740	LQNWFQHWRFCSQRAAQQRFQ
O15523	466	EDFLYHEGYACTSIHGDRSQR

P70695	129	DPLDGSSNIDCLASIGTIFAI
Q92616	2558	PAKLSSLFVKCLQNPSSDIRL
Q9D6R2	351	LTKDLGGNAKCSDFTEEICRR
O75828	150	SLQCLRAFENCSEDLQERFHS
P63323	108	GKPRKVVGCSCVVVKDYGKES
O35737	267	SDRFGRDLNYCFSGMSDHRYG
Q6R5N8	735	DVYGNKLQCTCDNLWFKNWSM
Q14690	510	GDEVKCRVLLCDPEAKKLMMT
P78527	1525	LELAFAFGGLCERLVSLLLNP
Q71FD7	337	GRNFHENCYRCEDCSVLLSVE
O75369	622	EPGEYAVHIMCDDEDIKDSPY
Q8BLF1	380	LDHFEDGFHGCMIFTSWPTNF
Q6NSR8	115	PGTHRCILMVCEQTEVFASAC
O70433	221	TCFCDLYAKKCAGCTNPISGL
O00233	59	GIGMNEPLVDCEGYPRSDVDL
P48725	1299	PRPPGPRDSQCVQLDEEVELL
Q71FD7	362	GCYPLNDHLFCKPCHLKRSAA
O15519	426	SHSSPSLYLQCLSQKLRQERK
Q9BQG0	305	KMQFWPASYLCFRLLGAALPL
Q9DCW4	71	IIAVSCGPSQCQETIRTALAM
Q8BRK8	302	AVKEVCEKFECTESEVMNSLY
P15532	145	QPEELVEYKSCAQNWIYEXXX
P40227	508	YCVKKQLLHSCTVIATNILLV
Q99798	27	VRQYHVASVLCQRAKVAMSHF
Q791T5	340	LVGDLMAVNNCGLRAGLPPYS
P23368	120	IVYTPTVGLACSQYGHIFRRP
P27635	12	GRRPARCYRYCKNKPYPKSRF
O70433	51	EECGTPIGCDCKDLSYKDRHW
Q8VDC0	849	QMAVLINNKACGKIPVPQHVA
Q01433	41	LDVAEPGPSRCRSDSPAVAAV
Q99MN9	383	SVKGARFVRFCDAFNIPLITF
Q921H8	408	GRRAYGVVSMCIGTGMGAAAV
O75694	651	NPATQATNMSCVTGPEIVYSG
Q8CI94	581	IHEYKRQLLNCLHIITLYNRI
P70398	739	PSLLTENGMKCFERFFKAVNC
Q9Y6I9	92	DNPHMVPPDKCRCAVGSILSE
B1AR13	21	DFIFLFPNHICLPALSKPYQR
O00468	682	GGSGSGEDGDCEQELCRQRGG
Q86VP6	71	NGEVQNLAVKCLGPLVSKVKE
Q9CPR5	187	FYDPRSLEILCKPIPFFLRGQ
O75153	184	KKGLEMDPIDCTPPEYILPGS
P00533	781	NPHVCRLLGICLTSTVQLITQ
A2ASS6	7209	IRPGGNYTITCVGNTPHLRIL
P09528	103	DWESGLNAMECALHLEKSVNQ
P70398	831	DRLKASYDTLCVLDGDKDSIN
P56399	532	LVRAQVPFSSCLEAYGAPEQV
Q9R0Q7	75	KHKRTDRSILCCLRKGESGQS
Q9CQA3	188	DREKLDGLYECILCACCSTSC
P50462	61	AHESEIYCKVCYGRRYGPKGI
Q9JHI5	379	ATQVALDGIQCLGGNGYINDF
P41250	444	ENEMAHYACDCWDAESKTSYG
O00468	414	VCLSRRGRPRCSCDRVTCDGA

Q3TC72	21	SALLQVQKRPCQPSRNMRLVQ
Q9JHR7	966	VHVLAREMDSCPVVGEFPSQN
P16332	558	ALAVDAARARCTVGEITDALK
Q9DCL9	288	LGHCEKIKKACGNFGIPCELR
O89103	440	DARGNPCDSLCFNTDGSFRCG
Q9Y3I1	359	VRSVLSLSAVCRDLFTASNDP
Q9DBC0	21	SLAVARTRPRCVGLALPSSAP
Q8TCG1	663	QADRLIAQHRCQRTQAETEAR
O55070	29	ALHDTLALRLCSFNVRSFGAS
Q64514	197	KYSDPGPVYDCLVWHDGETWR
Q9CW46	161	LVRPFGSLERCFLVYSERTGH
P23229	978	SVNVNCVNIRCPLRGLDSKAS
Q99615	164	EKRDFRKVVFCMDRALEFAPA
Q8BGD5	558	SHFGKSFIKCCHVSSDSFIQL
O75153	899	MTPQELWKNICQEAKNYFDFD
O75369	1876	ISCIDNKDGTCTVTYLPTLPG
Q8K4Q0	1168	VQDIPTGADSCVTSLSCDSHR
Q8BIF0	190	SYISYQQKKFCFSIQQGLNAD
Q8BUV3	502	PIGHDIKRGECVLAKGTHMGP
Q9HC38	197	RALLGYADNQCKLELQGVKGG
Q08211	578	YPVQEYFLEDCIQMTHFVPPP
Q91YE3	178	LKLALEYIVPCMNKHGICVVD
Q02248	429	CAAGILSNLTCNNYKNKMMVC
Q91ZA3	708	GKMGKVKLVHCKAGDTVGEGD
Q9QUR6	25	SVQEYHGHKICDPYSWLEDPD
Q8C460	258	WDLKVMLPGQCHYLGLPVADY
Q8K2M0	143	EAVRAEWERTCGPYHKQRLAE
Q8BGK2	158	LETLIEVSIECGRMTHNHPTG
Q62234	1010	GLGAPSTVSECFKCEEWTIAV
P41216	333	LAHMFETVVECVMLCHGAKIG
P49327	609	AVLAAYWRGQCIKEAHLPPGA
Q9JJL8	455	QFAHTVNATACAVPRVLIALL
Q62407	1413	IVYVVEGQPACVTVTFNHVEA
Q9CY45	149	VADPPYLSEECLRKTSETIQF
O00429	345	QLITKFATEYCNTIEGTAKYI
Q9CW46	251	ALSVVYTPTFCQLACGQDGQL
Q8TAT6	355	GDFQNKHPNMCRLSPDGHFGS
Q5UIP0	382	DSNASPQGNSCHVATSPGLNP
Q6PAR5	1432	VQYISSFYASCLSGEESYWWM
P35285	120	SIVVAIAGNKCDLTDVREVME
P15170	261	NPKKDIHFMPCSGLTGANLKE
Q6PAR5	702	PGSGSVLLDPCTGSTISETTS
