# Supplementary material for: iSNO-AAPair: incorporating amino acid pairwise coupling into PseAAC for predicting cysteine S-nitrosylation sites in proteins
Source: PeerJ. 2013 Oct 3;1:e171. doi: 10.7717/peerj.171 (PMC3792191; doi:10.7717/peerj.171)
Supplement: Supplemental Information S3 — The SNO site is marked with red, while non-SNO site with blue. [file peerj-01-171-s003.pdf]

Online Supporting Information S3. The sequences of 14 independent proteins whose experimental SNO sites are known but none of them occurs in $\mathbb{S}_L$ used to train iSNO-AAPair. The SNO site is marked with red, while non-SNO site with blue.

>sp|P61982|1433G_MOUSE 14-3-3 protein gamma OS=Mus musculus GN=Ywhag PE=1 SV=2
MVDREQLVQKARLAEQAERYDDMAAAMKNVTELNEPLSNEERNLLSVAYKNVVGARRSSWRV
ISSIEQKTSADGNEKKIEMVRAYREKIEKELEAVCQDVLSLLDNYLIKNCSETQYESKVFYL
KMKGDYYRYLAEVATGEKRATVVESSEKAYSEAHEISKEHMQPTHPIRLGLALNYSVFYYEI
QNAPEQACHLAKTAFDDAIAELDTLNEDSYKDSTLIMQLLRDNLTLWTSDQQDDDGGEGNN
>sp|P23528|COF1_HUMAN Cofilin-1 OS=Homo sapiens GN=CFL1 PE=1 SV=3
MASGVAVSDGVIKVFNDMKVRKSSTPEEVKKRKKAVLFCLSEDKKNIILEEGKEILVGDV
GQTVDDPYATFVKMLPDKDCRYALYDATYETKESKKEDLVFIFWAPESAPLKSKMIYASS
KDAIKKKLTGIKHELQANCYEEVKDRCTLAEKLGGSAVISLEGKPL
>sp|P13804|ETFA_HUMAN Electron transfer flavoprotein subunit alpha, mitochondrial OS=Homo sapiens GN=ETFA PE=1 SV=1
MFRAAAPGQLRRAASLLRFQSTLVIAEHANDSLAPITLNTITAATRLGGEVSCLVAGTKC
DKVAQDLCKVAGIAKVLVAQHDVYKGLLPEELTPLILATQKQFNYTHICAGASAFGKNLL
PRVAAKLEVAPISDIIAIKSPDTFVRTIYAGNALCTVKCDEKVKVFSVRGTSFDAAATSG
GSASSEKASSTSPVEISEWLDQKLTKSDRPELTGAKVVVSGGRGLKSGENFKLLYDLADQ
LHAAVGASRAAVDAGFVPNDMQVGQTGKIVAPELYIAVGISGAIQHLAGMKDSKTIVAIN
KDPEAPIFQVADYGIVADLFKVVPEMTEILKKK
>sp|O35295|PURB_MOUSE Transcriptional activator protein Pur-beta OS=Mus musculus GN=Purb PE=1 SV=3
MADGDSGSERGGGGGGGGGPGGFQPAPRGGGGGGGGPGGEQETQELASKRLDIQNKRFYLDVKQN
AKGRFLKIAEVGAGGSKSRLTLSMAVAAEFRDSLGDFIEHYAQLGPSSPEQLAAGAEEGGGPRRA
LKSEFLVRENRKYYLDLKENQRGRFLRIRQTVNRGGGGFGGGPGPGGLQSGQTIALPAQGLIEFR
DALAKLIDDYGGDEDELAGGPGGGAGGPGGGLYGELPEGTSITVDSKRFFFDVGCNKYGVFLRVS
EVKPSYRNAITVPFKAWGKFGGAFCRYADEMKEIQERQRDKLYERRGGGSGGGDESEGEEVDED
>sp|Q9DC70|NDUS7_MOUSE NADH dehydrogenase [ubiquinone] iron-sulfur protein 7, mitochondrial OS=Mus musculus GN=Ndufs7 PE=1 SV=1
MAALAAPGLLSVRILGLRTAQVQLRRVHQSVATEGPSPSPSPSLSSTQSAVSKAGAGAVV
PKLSHLPRSRAEYVVTKLDDLINWARRSSLWPMTFGLACCAVEMMHMAAPRYDMDRFGVVFRASP
RQADVMIVAGTLTNKMAPALRKVYDQMPEPRYVVSMGSCANGGGYYHYSYSVVRGCDRIVPVDIY
VPGCPPTAEALLYGILQLQRKIKREQKLKIWYRR
>sp|P19338|NUCL_HUMAN Nucleolin OS=Homo sapiens GN=NCL PE=1 SV=3
MVKLAKAGKNQGDPKKMAPPPKEVEEDSEDEEMSEDEEDDSSGEEVVIPQKKGKKAAATSAKKVV
VSPTKKVAVATPAKKAAVTPGKKAAATPAKKTVTPAKAVTTPGKKGATPGKALVATPGKKGAAIP
AKGAKNGKNAKKEDSDEEEDDDSEEDEEDDEDEDEDEDEIEPAAMKAAAAAPASEDEDDEDDEDD
EDDDDDEEDDSEEEAMETTPAKGKKAAKVVPVKAKNVAEDEDEEEDDEDEDDDDDEDDEDDDDED
DEEEEEEEEEEPVKEAPGKRKKEMAKQKAAPEAKKQKVEGTEPTTAFNLFVGNLNFNKSAPELKT
GISDVFAKNDLAVVDVRIGMTRKFGYVDFESAEDLEKALELTGLKVFGNEIKLEKPKGKDSKKER
DARTLLAKNLPYKVTQDELKEVFEDAAEIRLVSKDGKSKGIAYIEFKTEADAEKTFEEKQGTEID
GRSISLYYTGEKGQNQDYRGGKNSTWSGESKTLVLSNLSYSATEETLQEVFEKATFIKVPQNQNG
KSKGYAFIEFASFEDAKEALNSCNKREIEGRAIRLELQGPRGSPNARSQPSKTLFVKGLSEDTTE
ETLKESFDGSVRARIVTDRETGSSKGFGFVDFNSEEDAKAAKEAMEDGEIDGNKVTLDWAKPKGE
GGFGGRGGGRGGFGGRGGGRGGRGGFGGRGRGGFGGRGGFRGGRGGGGDHKPQGKKTKFE
>sp|Q9NPA8|ENY2_HUMAN Enhancer of yellow 2 transcription factor homolog OS=Homo sapiens GN=ENY2 PE=1 SV=1
MVVSKMNKDAQMRAAINQKLIETGERERLKELLRAKLIECGWKDQLKAHCKEVIKEKGLEHVTVD
DLVAEITPKGRALVPDSVKKELLQRIRTFLAQHASL
>sp|Q9R0P5|DEST_MOUSE Destrin OS=Mus musculus GN=Dstn PE=1 SV=3
MASGVQVADEVCRIFYDMKVRKCSTPEEIKKRKKAVIFCLSADKKCIVVEEGKEILVGDV
GATITDPFKHFVGMLPEKDCRYALYDASFETKESRKEELMFFLWAPEQAPLKSKMIYASS
KDAIKKKFPGIKHEYQANGPEDLNRTCIAEKLGGSLIVAFEGSPV
>sp|P48444|COPD_HUMAN Coatomer subunit delta OS=Homo sapiens GN=ARCN1 PE=1 SV=1
MVLLAAAVCTKAGKAIVSRQFVEMTRTRIEGLLAAFPKLMNTGKQHTFVETESVRYVYQPMEKLY

MVLITTKNSNILEDLETLRLFSRVIPEYCRALEENEISEHCFDLIFAFDEIVALG
YRENVNLAQIRTFTEMDSHEEKVFRAVRETQEREAKAEMRRKAKELQQARRDAERQGKKAPGFGG
FGSSAVSGGSTAAMITETIIETDKPKVAPAPARPSGPSKALKLGAKGKEVDNFVDKLKSEGETIM
SSSMGKRTSEATKMHAPPINMESVHMKIEEKITLTCGRDGGLQNMELHGMIMLRISDDKYGRIRL
HVENEDKKGVQLQTHPNVDKKLFTAESLIGLKNPEKSFPVNSDVGVLKWRLQTTEESFIPLTINC
WPSESGNGCDVNIEYELQEDNLELNDVVITIPLPSGVGAP
VIGEIDGEYRHDSRRNTLEWCLPVIDAKNKSGSLEFSIAGQPNDFFPVQVSFVSKKNYCN
IQVTKVTQVDGNSPVRFSTETTFLVDKYEIL
>sp|Q8VDG5|PPCS_MOUSE Phosphopantothenate--cysteine ligase OS=Mus musculus GN=Ppcs PE=2 SV=1
MAEMDLVAELPRPAGAARWAEVMARFAARLGEQGRRVVLITSGGTKVPLEARAVRFLDNFSSGRR
GAASAEVFLAAGYGVLFLYRARSAFPYAHRFPPQAWLSALRPSGPAQSGKLSLEA
EENALPGFAAALQSYQEAAAAGTFLAVEFTTLADYLHLLQAAALALSPLGSSAMFYLAAAVSDFY
IPVSEMPEHKIHSSGGPLQITMKMVPKMLSPLVKDWAPKAFVVSFKLETDPDIII
SRARNALEVYQHQVVVANILESIKSFVIIVTKDSETELLLSEEEVAKGLVIEEKIVDDLR
SRHTAFICDKN
>sp|O09110|MP2K3_MOUSE Dual specificity mitogen-activated protein kinase kinase 3 OS=Mus musculus GN=Map2k3 PE=1 SV=2
MESPAASPPASLPQTKGKSKRKKDLRISCVSKPPVSNPTPPRNLDSRTFITIGDRNFEVE
ADDLVTISELGRGAYGVVEKVRHAQSGTIMAVKRIRATVNTQEQKRLLMDLDINMRTVDCFYTVT
FYGALFREGDVWICMELMDTSLDKFYRKVLEKNMKIPEDILGEIAVSIVRALEHL
HSKLSVIHRDVKPSNVLINKEGHVKMCDFGISGYLVDSVAKTMDAGCKPYMAPERINPEL
NQKGYNVKSDVWSLGITMIEMAILRFPYESWGTPFQQLKQVVEEPSPQLPADQFSPEFVD
FTSQCLRKNPAERMSYLELMEHPFFTLHKTKKTDIAAFVKEILGEDS
>sp|O75390|CISY_HUMAN Citrate synthase, mitochondrial OS=Homo sapiens GN=CS PE=1 SV=2
MALLTAAARLLGTKNASCLVLAARHASASSTNLKDILADLIPKEQARIKTFRQQHGKTVV
GQITVDMMYGGMRGMKGLVYETSVLDPDEGIRFRGFSIPECQKLLPKAKGGEEPLPEGLF
WLLVTGHIPTEEQVSWLSKEWAKRAALPSHVVTMLDNFPTNLHPMSQLSAAVTALNSESNFARAY
AQGISRTKYWELIYEDSMDLIAKLPCVAAKIYRNLYREGSGIGAIDSNLDWSHNFTNMLGYTDHQ
FTELTRLYLTIHSDHEGGNVSAHTSHLVGSALSDPYLSFAAAMNGLAGPLHGLANQEVLVWLTQL
QKEVGKDVSDEKLRDYIWNTLNSGRVVPGYGHAVLRKTDPRYTCQREFALKHLPNDPMFKLVAQL
YKIVPNVLLEQGKAKNPWPNVDAHSGVLLQYYGMTEMNYYTVLFGVSRALGVLAQLIWSRALGFP
LERPKSMSTEGLMKFVDSKSG
>sp|P56965|DDAH1_BOVIN N(G),N(G)-dimethylarginine dimethylaminohydrolase 1 OS=Bos taurus GN=DDAH1 PE=1 SV=3
MASLGHPATFGRATHVVVRALPESLAQQALRRTKGDEVDFARAERQHQLYVGVLGSKLGLQVVQL
PADESLPDCVFVEDVAVVCEETALITRPGAPSRRKEADMMKEALEKLQLNIVEMKDENATLDGGD
VLFTGREFFVGLSKRTNQRGAEILADTFKDYAVSTVPVVDALHLKSFCSMAGPNLIAIGSSESAQ
KALKIMQQMSDHRYDKLTVPDDTAANCIYLNIPSKGHVLLHRTPEEYPESAKVYEKLKDHMLIPV
SNSELEKVDGLLTCSSVLINKKVDS
>sp|Q9QZX7|SRR_MOUSE Serine racemase OS=Mus musculus GN=Srr PE=1 SV=1
MCAQYCISFADVEKAHINIQDSIHLTPVLTSSILNQIAGRNLFFKCELFQKTGSFKIRGA
LNAIRGLIPDTPEEKPKAVVTHSSGNHGQALTYAAKLEGIPAYIVVPQTAPNCKKLAIQA
YGASIVYCDPSDESREKVTQRIMQETEGILVHPNQEPAVIAGQGTIALEVLNQVPLVDAL
VVPVGGGGMVAGIAITIKALKPSVKVYAAEPSNADDCYQSKLKGELTPNLHPPETIADGV
KSSIGLNTWPIIRDLVDDVFTVTEDEIKYATQLVWGRMKLLIEPTAGVALAAVLSQHFQT
VSPEVKNVCIVLSGGNVDLTSLNWVGQAERPAPYQTVSV
