# Supplementary material for: iSNO-AAPair: incorporating amino acid pairwise coupling into PseAAC for predicting cysteine S-nitrosylation sites in proteins
Source: PeerJ. 2013 Oct 3;1:e171. doi: 10.7717/peerj.171 (PMC3792191; doi:10.7717/peerj.171)
Supplement: Supplemental Information S4 [file peerj-01-171-s004.pdf]

Online Supporting Information S4. Detailed results predicted by various predictors[a] on the 14 independent proteins in Online Supporting Information S3

Protein code	Observed SNO site	Predicted SNO site[b]		
		GPS-SNO[c]	iSNO-PseAAC[d]	iSNO-AAPair
P61982	112	97;112;194	194	112
P23528	147	139	39;80;147	39;139;147
P13804	53	53;159	53;109;159;194	53;60;68;155
O35295	250	None	250	285
Q9DC70	164	None	194	164;194
P19338	543	None	543	543
Q9NPA8	40	None	40;50	40;50
Q9R0P5	23	23	23;39;46	12;23;147
P48444	441	9	389;441;444	286;479
Q8VDG5	308	None	308	308
O09110	29	120	29;120	207;227
O75390	211	103;202;221;479;585;928;1105;2038	101;211;359	18;101;211
P56965	222;274	274	84;274	222;274
Q9QZX7	113	2;6;113;309	46;113;128;217	113;217

[a] The results for the method proposed in [1] and that in [2] were not listed because the former had no web-server and latter's web-server did not work.

[b] The number colored with red means the SNO site of correct prediction.

[c] The method proposed in [3] where the threshold parameter was set at "medium" to get its highest overall accuracy.

[d] The method proposed in [4].

REFERENCES

1. Li BQ, Hu LL, Niu S, Cai YD, Chou KC (2012) Predict and analyze S-nitrosylation modification sites with the mRMR and IFS approaches. J Proteomics 75: 1654-1665.
2. Li YX, Shao YH, Jing L, Deng NY (2011) An efficient support vector machine approach for identifying protein S-nitrosylation sites. Protein Pept Lett 18: 573-587.
3. Xue Y, Liu Z, Gao X, Jin C, Wen L, et al. (2010) GPS-SNO: computational prediction of protein S-nitrosylation sites with a modified GPS algorithm. PLoS One 5: e11290.
4. Xu Y, Ding J, Wu LY, Chou KC (2013) iSNO-PseAAC: Predict cysteine S-nitrosylation sites in proteins by incorporating position specific amino acid propensity into pseudo amino acid composition PLoS ONE 8: e55844.
